# Methodological and reporting quality of systematic reviews on health effects of air pollutants were higher than extreme temperatures: a comparative study

**DOI:** 10.1186/s12889-023-17256-5

**Published:** 2023-11-29

**Authors:** Xuping Song, Qiyin Luo, Liangzhen Jiang, Yan Ma, Yue Hu, Yunze Han, Rui Wang, Jing Tang, Yiting Guo, Qitao Zhang, Zhongyu Ma, Yunqi Zhang, Xinye Guo, Shumei Fan, Chengcheng Deng, Xinyu Fu, Yaolong Chen, Kehu Yang, Long Ge, Shigong Wang

**Affiliations:** 1 Evidence-Based Social Science Research Center, Department of Social Medicine and Health Management, School of Public Health, Lanzhou University, Lanzhou, China; 2 Evidence-Based Medicine Center, School of Basic Medical Sciences, Lanzhou University, Lanzhou, China; 3 Key Laboratory of Evidence Based Medicine & Knowledge Translation of Gansu Province, Lanzhou, China; 4 College of Atmospheric Sciences, Chengdu University of Information Technology, Chengdu, Sichuan China; 5 The Fifth Hospital of Xi’an, Xi’an, China; 6 School of Stomatology, Lanzhou University, Lanzhou, China; 7 Institute of Health Data Science, Lanzhou University, Lanzhou, China; 8 WHO Collaborating Centre for Guideline Implementation and Knowledge Translation, Lanzhou, China; 9 McMaster Health Forum, Department of Health Research Methods, Evidence, and Impact, McMaster University, Hamilton, L8S4L8 Canada

**Keywords:** Reporting quality, Methodological quality, Systematic review, Air pollution, Temperature

## Abstract

**Background:**

An increasing number of systematic reviews (SRs) in the environmental field have been published in recent years as a result of the global concern about the health impacts of air pollution and temperature. However, no study has assessed and compared the methodological and reporting quality of SRs on the health effects of air pollutants and extreme temperatures. This study aims to assess and compare the methodological and reporting quality of SRs on the health effects of ambient air pollutants and extreme temperatures.

**Methods:**

PubMed, Embase, the Cumulative Index to Nursing and Allied Health Literature (CINAHL), Cochrane Library, Web of Science, and Epistemonikos databases were searched. Two researchers screened the literature and extracted information independently. The methodological quality of the SRs was assessed through A Measurement Tool to Assess Systematic Reviews 2 (AMSTAR 2). The reporting quality was assessed through Preferred Reporting Items of Systematic reviews and Meta-Analyses (PRISMA).

**Results:**

We identified 405 SRs (286 for air pollution, 108 for temperature, and 11 for the synergistic effects). The methodological and reporting quality of the included SRs were suboptimal, with major deficiencies in protocol registration. The methodological quality of SRs of air pollutants was better than that of temperature, especially in terms of satisfactory explanations for any heterogeneity (69.6% v. 45.4%). The reporting quality of SRs of air pollution was better than temperature, however, adherence to the reporting of the assessment results of risk of bias in all SRs (53.5% v. 34.3%) was inadequate.

**Conclusions:**

Methodological and reporting quality of SRs on the health effect of air pollutants were higher than those of temperatures. However, deficiencies in protocol registration and the assessment of risk of bias remain an issue for both pollutants and temperatures. In addition, developing a risk-of-bias assessment tool applicable to the temperature field may improve the quality of SRs.

**Supplementary Information:**

The online version contains supplementary material available at 10.1186/s12889-023-17256-5.

## Background

The environmental consequences of climate change such as increasing air pollution and extreme temperature events are impacting human health and lives [1]. Ambient air pollution, including sulfur dioxide (SO_2_), carbon monoxide (CO), nitrogen dioxide (NO_2_), particulate matter (PM), such as PM_2.5_, PM_10_ PM_1_, black carbon (BC) and ultrafine particles (UFP), and Ozone(O_3_), is the fourth contributing factor to death worldwide [2, 3]. According to the World Health Organization (WHO), nine out of ten people around the world breathe polluted air, contributing to 7.0 million deaths each year [3]. In addition, the 2017 Global Burden of Disease study (GBD) reported 4.6 million deaths from PM and 500,000 deaths from O_3_ [4]. Compared to 2017, the burden of PM increased by 44.6% in 2019 [5]. Therefore, health effects attributable to short-term and long-term air pollutants exposure have been a major threat shared by people worldwide [1].

Extreme temperature events, such as heat waves, cold spells, extreme heat, and extreme cold, are significant contributors to climate change and major risk factors for human health, causing increasing concern among governments and the general public [6]. GBD 2019 adds three risk factors: non-optimal temperature, high temperature, and low temperature, with the non-optimal temperature being the tenth risk factor for the death of women in 2019 (940,000 deaths) [5]. Due to rapid temperature rise, vulnerable populations have been exposed to 3.7 billion more person-days of heatwaves in 2021 than annually in 1986–2005 [7]. Extreme temperature events can directly affect health, and they can also affect physical and mental well-being through less direct pathways such as the recurrence of infectious diseases [8]. Additionally, there are synergistic effects between air pollution and temperature. Some studies have shown that extreme heat significantly increases the impacts of air pollutants such as PM_10_ and O_3_ on mortality, and extreme cold increases the health impacts of PM_10_ on respiratory disease [9–11].

A large number of SRs in the environmental field have been published recently as a result of the global concern about the health impacts of air pollution and temperature. Systematic reviews are regarded as high-level evidence and can provide accurate, succinct, credible, and comprehensive summative evidence for policy making [12]. As an evidence-based practice, it is one of the key tools for guideline developers and policy-makers [13]. Methodology and reporting are two crucial steps for SRs, the quality of which is key to producing a high-quality systematic review. Methodological quality determines whether the evidence is robust while reporting quality reflects the completeness and comprehensiveness of the SRs [14, 15].

Some previous studies [16–19] have found that the methodological and reporting quality of published SRs were suboptimal. Sheehan et al. have reviewed environmental health SRs published from 1990 to 2013 and ambient air pollution SRs published from 2009 to 2015, using a questionnaire consisting of major items shared by several guidelines, such as the PRISMA checklist and Meta-Analysis of Observational Studies in Epidemiology (MOOSE) statement [20, 21]. A limitation of systematic reviews conducted by Sheehan et al. is that they merely focused on reporting quality of environmental health SRs. However, the methodological quality determines the strength of evidence. The number of SRs for air pollutants and temperatures has significantly expanded, with more than 300 SRs published in 2015 and beyond. No recent review that we are aware of has updated the research.

We don’t know how recently the quality of SR in environmental health has been. Therefore, this study aims to (i) evaluate the methodological and reporting quality of SRs in environmental health, focusing on the health effects of ambient air pollution and temperature, using AMSTAR 2 and PRISMA checklist; (ii) compare the methodological and reporting quality of SRs on health effects of ambient air pollution and temperature.

## Methods

### Literature search

Six electronic databases were searched to identify relevant literature: PubMed, Embase, CINAHL, the Cochrane Library, Web of Science, and Epistemonikos. The search was limited to English language publications and covered articles published from database inception until July 1, 2022, and then updated to October 9, 2023. Medical Subject Headings (MeSH) and free text were reviewed along with specific keywords to construct a comprehensive search strategy, based on the PEOS principles (Population, Exposure, Outcome, and Study design). The full search strategies are available in Additional file 1. Table S1.

### Literature selection

Literature screening was performed independently by two researchers. When the opinions of the two reviewers differed, differences were resolved through consultation with a third reviewer. Two reviewers screened all titles and abstracts and any full-text retrieved, to determine eligibility. Endnote X9 software was used to identify and reject duplicates. The inclusion and exclusion criteria were based on PEOS principles.

### The inclusion criteria

i Population: no restriction on disease types;
ii Exposure: temperature (e.g., extreme heat, extreme cold, heat waves, cold waves) and/or ambient air pollutants (e.g., PM_10_, PM_2.5_, PM_1_, SO_2_, NO_2_, O_3_, CO, BC, UFP) and/or greenhouse gases from climate change (e.g., CO_2_, CH_4_, N_2_O, HFCs, PFCs, SF_6_);
iii Outcome: morbidity or mortality;
iv Study design: systematic reviews and/or meta-analyses.

### The exclusion criteria

(i) Studies focusing only on indoor exposure or special site exposure; (ii) Studies that considered only seasonal effects rather than temperature impacts ; (iii) Animal studies; (iv) Articles not published in English.

### Data extraction

Data extraction was performed independently by two reviewers using a predesigned table in Microsoft Excel 2021, with disagreements resolved by consultation with a third reviewer. Extractions included authors, year of publication, corresponding author’s country, journal of publication, type of exposure, type of disease, study outcome, type of systematic reviews (narrative or meta-analysis), and the Impact Factors (IF) of the journals in Journal Citation Reports (JCR) in 2021.

### Quality assessment

The methodological and reporting quality evaluation processes were conducted independently by two trained researchers, with disagreements discussed with a third researcher to reach a consensus.

The methodological quality of the included SRs was evaluated through the AMSTAR 2 tool, which was published in the *British Medical Journal* (*BMJ*) in 2017 to evaluate the methodological quality of SRs [13]. AMSTAR 2 consists of 16 items (with 7 critical domains: items 2, 4, 7, 9, 11, 13, 15), and each item was evaluated as “Yes”, “Partial Yes”, “No”, or “Not Applicable”. The quality level was rated as “High”, “Moderate”, “Low”, and “Critically Low” [13].

The reporting quality of included studies was evaluated using the PRISMA checklist, which was disseminated in 2009 in *BMJ* as a reporting guideline for SRs [22]. Since its publication, PRISMA has gained international endorsement. PRISMA contains 7 modules with 27 items: title, abstract, introduction, methods, results, discussion, and funding. Each of the items was assigned a value of 1 if it was “total compliance”, a value of 0.5 if it was “partial compliance”, and a value of 0 if it was “no compliance”. The values of each item were added up to provide a final score, with a total maximum score of 27. An SR with a score ≤ 15 was considered to have major flaws, 15.5–21 as having minor flaws, and ≥ 21.5 as having minimal flaws [23].

### Data synthesis and analysis

Data were extracted, managed, and analyzed using Microsoft Excel 2021. The Chi-square test was performed using IBM SPSS Statistics 26.0.0.2 for differences in proportions of the evaluation results of each item (or Fisher’s exact test if a contingency table contained cells with five or fewer events).

## Results

A total of 33,292 records were identified. Of the 405 SRs that met the inclusion criteria, 286 were for air pollution, 108 for temperature, and 11 for interaction between air pollution and temperature (Additional file 1. Fig. S1). No articles on the health effects of greenhouse gases were included.

### Characteristics of included systematic reviews

Table 1 provides an overview of the key features of the included studies. (i) The studies were published between 2001 and 2023. More than half of them were published during the last 5 years (n = 250), suggesting that the major of them were proposed after the publication of AMSTAR 2 and PRISMA. (ii) Of the included studies, 68.4% were more likely to conduct meta-analyses, rather than qualitative systematic reviews. (iii). The average IF of the included 302 studies was 8.546 ± 1.480, whereas 17 papers [24–40] had no impact factor. SRs of air pollution were most frequently published in journals with an IF of 5 to 10 (44.1%), while SRs of temperature were mostly published in journals with an IF < 5 (43.5%). (iv) SRs of air pollution focused more on respiratory diseases (31.5%), cardiovascular diseases (28.7%), and all-cause mortality (18.2%), while SRs of temperature were more likely to study all-cause mortality (39.8%), infectious diseases (15.7%), and cardiovascular diseases (13.9%).

**Table 1:** The characteristics and results of the methodological and reporting quality of the included systematic reviews and meta-analyses

	Air Pollution (n = 286,%)	Temperature (n = 108,%)	Interaction (n = 11,%)	Total (n = 405,%)
**Type of article**				
SR	64 (22.4)	58 (53.7)	6 (54.5)	128 (31.6)
SR and MA	222 (77.6)	50 (46.3)	5 (45.5)	277 (68.4)
**Year of publication**				
Before 2009	12 (4.2)	1 (0.9)	0	13 (3.2)
2009–2013	18 (6.3)	12 (11.1)	0	30 (7.4)
2014–2018	71 (24.8)	39 (36.1)	2 (18.2)	112 (27.7)
2019–2023	185 (64.7)	56 (51.9)	9 (81.8)	250 (61.7)
**Journals of impact factor (2021)**				
[10,+∞)	55 (19.2)	19 (17.6)	3 (27.3)	77 (19.0)
[5–10)	126 (44.1)	33 (30.6)	3 (27.3)	162 (40.0)
(0,5)	98 (34.3)	47 (43.5)	4 (36.4)	149 (36.8)
None	7 (2.4)	9 (8.3)	1 (9.1)	17 (4.2)
**AMSTAR 2**				
High	3 (1.0)	0	0	3 (0.7)
Moderate	7 (2.4)	0	0	7 (1.7)
Low	49 (17.1)	10 (9.3)	0	59 (14.6)
Critically Low	227 (79.4)	98 (90.7)	11 (100.0)	336 (83.0)
**PRISMA**				
Minimal Flaws	148 (51.7)	33 (30.6)	4 (36.4)	185 (45.7)
Minor Flaws	88 (30.8)	27 (25.0)	2 (18.2)	117 (28.9)
Major Flaws	50 (17.5)	48 (44.4)	5 (45.5)	103 (25.4)

*Abbreviations: SR = systematic review; MA = Meta-analysis; AMSTAR 2 = A Measurement Tool to Assess Systematic Reviews 2; PRISMA = Preferred Reporting Items of Systematic reviews and Meta-Analyses

### Results of methodological quality

The methodological quality of the included studies was suboptimal, with only 0.7% of high quality. The main results of each item were as follows (Additional file 1. Table S2). (i) All the included studies were rated “Yes” for item 1 (PICO: populations, interventions, comparisons, and outcomes) and item 11 (appropriate methods). (ii) Of the included studies, 26.6% justified the study protocol and registration, 5.6% provided a list of the excluded studies and justified the exclusions, but only 3.5% explained their selection of study designs for inclusion in the review. (iii) Only 0.7% of studies reported the source of funding for the studies included in the review (The detailed results can be found in Additional file 1. Table S4).

### Results of reporting quality

The reporting quality of the included studies needs further improvement, with only 45.7% having minimal flaws. The main results for each item were as follows (Additional file 1. Table S3). (i) All the included studies reported item 3 (rationale), and item 7 (information sources of methods). All the included studies reported item 2 (abstract), with only 19% of them reporting the structured summary completely and most of the studies reporting it partially. (ii) Only 26.4% of the included studies reported item 5 (protocols and registration). (iii) Less than 70% of the included studies reported on items 12, 15, 19, and 22 (55.6%, 57.3%, 47.9%, and 50.6%, respectively), which required authors to describe methods used and conclusions reached in the assessment of risk of bias within and across studies (The detailed results can be found in Additional file 1. Table S5).

**Fig. 1:**
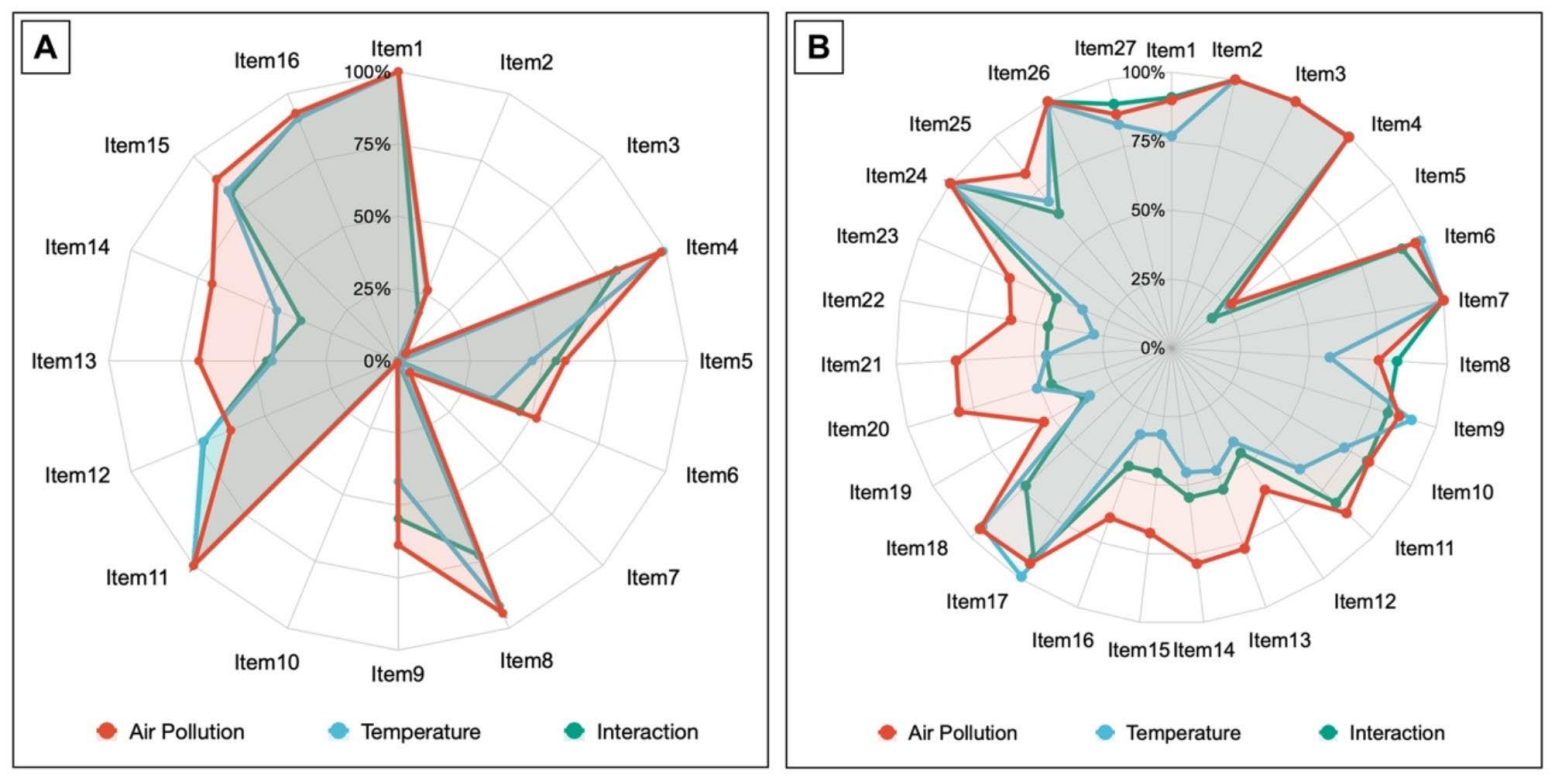
The methodological and reporting quality of the included systematic reviews and Meta-analyses (A. AMSTAR 2 score; B. PRISMA score)

### The comparison of methodologic and reporting quality of included studies

We conducted a comparison of the methodological and reporting quality of the SRs for air pollution and temperature using the compliance rate for each item (Fig. 1). The SRs of air pollution had generally higher methodological quality than that of temperature, especially in item 5 (Study selection in duplicate) (57.7% v. 46.3%) (*P* < 0.05), item 6 (Data extraction in duplicate) (51.7% v. 35.2%) (*P* < 0.01), and item 14 (Satisfactory explanation for and discussion of any heterogeneity) (69.6% v. 45.4%) (*P* < 0.001). Furthermore, the methodological quality of air pollution SRs was superior than that of temperature SRs in item 9 and 13, but lower than that of temperature SRs in item 12 (*P* < 0.05). (The detailed results can be found in Additional file 1. Table S2 and Table S3).

The SRs of air pollution also had higher reporting quality than that of temperature, especially in terms of reporting the methods of the risk of bias in individual studies (item 12) (61.5% v. 40.7%) (*P* < 0.001) and reporting the results of the risk of bias within studies (item 19) (53.5% v. 34.3%) (*P* < 0.001). Additionally, the reporting quality of air pollution SRs was higher than that of temperature SRs in item 1, 8, 10, 11, 13, 14, 15, 16, 20, 21, 22, 23, and 25, but lower than that of temperature SRs in item 17 (*P* < 0.05). (The detailed results can be found in Additional file 1. Table S2 and Table S3).

**Fig. 2:**
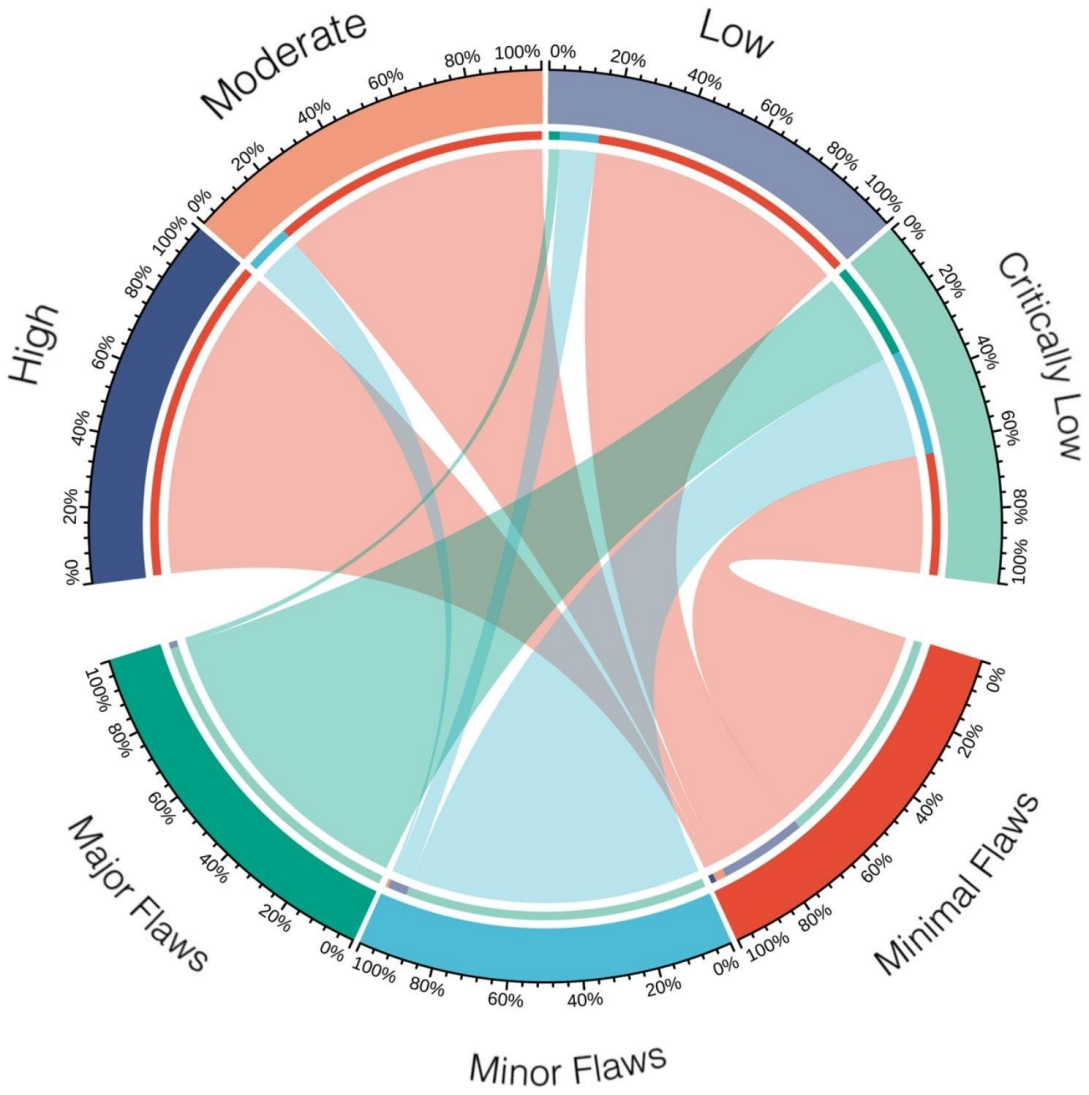
Comparison of the methodological and reporting quality of the included systematic reviews

When comparing the overall quality of methodology with that of reporting (Fig. 2), the results of AMSTAR 2 and PRISMA evaluations reached similar conclusions. The three SRs with high methodological quality had minimal flaws in reporting quality. Of the seven SRs with moderate methodological quality, six had minimal flaws in reporting quality and one had minor flaws. Of the 59 SRs with low methodological quality, 84.7% (n = 50) had minimal flaws, 11.9% (n = 7) had minor flaws, and 3.4% (n = 2) had major flaws in reporting quality. Of the 336 SRs with low methodological quality, 37.5% (n = 126) had minimal flaws, 32.4% (n = 109) had minor flaws, and 30.0% (n = 101) had major flaws in reporting quality. Correspondingly, the 103 SRs with major flaws in reporting quality were of low (n = 2, 1.9%) or very low (n = 101, 98.1%) methodological quality. Of the 117 SRs with minor flaws in reporting quality, 0.9% (n = 1) had moderate methodological quality, 6.0% (n = 7) had low methodological quality, and 93.2% (n = 109) had critically low methodological quality. Of the 185 SRs that reported minimal flaws, 1.6% (n = 3) had high methodological quality, 3.2% (n = 6) had moderate methodological quality, 27.0% (n = 50) had low methodological quality, and 68.1% (n = 126) had critically low methodological quality.

## Discussion

SRs of the health impacts of ambient air pollution and extreme temperature can provide comprehensive and credible evidence for environmental policymaking and guidelines development [1]. With the increasing burden on human health resulting from air pollution and temperature change, the number of SRs in this field has greatly increased in recent years (61.7% in the last five years). However, this study shows that the methodological and reporting quality of SRs of air pollution and temperature health effects were below an acceptable level, highlighting an urgent need to improve the design and conduct of SRs.

### Satisfactory explanation for any heterogeneity should be provided

The methodological quality determines the reliability of SRs and, therefore, determines whether the guidelines and policies developed from these SRs provide actionable recommendations [16, 41]. In this study, only 62.2% of the included studies provided a satisfactory explanation for and discussion of any heterogeneity observed in the results. Strict and uniform inclusion and exclusion criteria should be established to only include studies sufficiently homogeneous in terms of participants, interventions, and outcomes for a Meta-analysis [42]. Both air pollution and extreme temperature contribute to human health burdens, yet different kinds of air pollutants always coexist with varied temperatures [10]. Therefore, the sources of heterogeneity should be reasonably explained and their impact on the results should be thoroughly discussed [42]. In addition, subgroup analysis can be performed by dividing the studies into different subgroups according to their different characteristics such as gender and age to reduce heterogeneity. If the heterogeneity is too high to be resolved, meta-analysis can be abandoned [42].

### Protocol registration in advance should be conducted

The reporting quality reflects the transparency of the SRs, which protocol registration can help to improve [19]. The present study shows that only 26.4% of the included studies reported protocol registration information, which aligns with the previous studies [16, 19]. The importance of protocol registration has led to the development of several platforms for it. The WHO clinical trial registration platform went online in 2008. The prospective systematic review registration project at the University of York, UK, was launched in 2011. Furthermore, the international practice guideline registration platform was launched in 2014. Cochrane SRs authors are required to register on the Cochrane Collaboration Network, whereas non-Cochrane SRs authors can register their protocols through the PROSPERO platform or publish their plans in journals [42]. Conducting a protocol and registering it in advance provide not only a clear path for researchers but also a reference for readers.

### The risk of bias should be properly assessed

The cornerstones of SRs are their included studies, of which the risk of bias can affect the authenticity and the quality of SRs [41]. Reporting the assessment of the risk of bias within and across studies adds to the reliability of SRs [41]. We found the SRs of air pollution performed better than those of temperature in study selection, data extraction, and the assessment and interpretation of risk of bias *(P* < 0.05). The mainstream risk of bias assessment tool commonly used for SRs is the Cochrane Risk of Bias (RoB) [43], which is an essential tool for the quality evaluation of Randomized Controlled Trials (RCTs). In line with the previous study, we also found that there is no risk of bias tool applicable to air pollutants SRs, with some studies using The Newcastle-Ottawa Scale (NOS) and some using self-developed items for risk of bias assessment [20]. WHO Global Air Quality Guidelines Risk of Bias Assessment Working Group produced a risk of bias assessment tool for air quality and health epidemiology studies in 2020 [44], aiming to assess the risk of bias of long-term and short-term exposure to air pollution in cohort, case-control, time-series, case-crossover, and panel studies. Yet there is no risk of bias assessment tool specially designed for temperature research. Therefore, the development of a risk of bias assessment tool applicable to the temperature field is urgently needed.

### Strengths and limitations

There are some strengths in our study. To our knowledge, this is the first attempt to compare the methodological and reporting quality of SRs for air pollution with those for temperature, and highlighted items with significant issues such as protocol registration and risk of bias assessment. Additionally, this study provided a complete and scientific evaluation of the present methodological and reporting quality of SRs on ambient air pollution and extreme temperature, which is of practical value for policy makers. Well-designed epidemiological studies and SRs are required to better understand the specific health impacts associated with ambient air pollution and extreme temperature.

Some limitations must also be acknowledged. We only included SRs published in English. However, we believe the results of our studies would have remained the same even if SRs in languages other than English had been consulted. Data extraction and quality assessment were performed following the data without further investigating the potential that the authors may have undertaken certain analyses but have not presented them in the paper. It is therefore possible that the methodological quality of the studies could be underestimated.

### Recommendations for future research

In recent years, the climate-health link has been the target of a growing body of research and the focus of the general public and the health professionals. To guarantee the scientificity and transparency of the SRs, we believe that apart from following methodological specifications to develop SRs, future researchers should develop, register, or publish the study protocols on public platforms. Additionally, it is urgently needed that a risk-of-bias assessment tool be developed in the future to direct future research.

## Conclusions

The methodological and reporting quality of SRs for air pollution were better than those for temperature. However, deficiencies in protocol registration and risk of bias assessment remain issues. Future reviewers should adhere to the methodological specifications and recognize the significance of pre-study protocol planning. Developing a risk-of-bias assessment tool applicable to the temperature field may also improve the quality of SRs in this field.

### Electronic supplementary material

Below is the link to the electronic supplementary material.

Supplementary Material 1

Supplementary Material 2

## Data Availability

All data generated or analyzed during this study are included in this published article and its supplementary information files.

## Abbreviations

SR: Systematic Review
CINAHL: Cumulative Index to Nursing and Allied Health Literature
AMSTAR 2: A Measurement Tool to Assess Systematic Reviews 2
PRISMA: Preferred Reporting Items of Systematic reviews and Meta-Analyses
SO_2_: Sulfur Dioxide
CO: Carbon Monoxide
NO_2_: Nitrogen Dioxide
PM: Particulate Matter
BC: Black Carbon
UFP: Ultrafine Particles
O_3_: Ozone
WHO: World Health Organization
GBD: Global Burden of Disease
PM: Particulate Matter
MOOSE: Meta-Analysis of Observational Studies in Epidemiology
MeSH: Medical Subject Headings
IF: Impact Factors
JCR: Journal Citation Reports
BMJ: British Medical Journal
RoB: Risk of Bias
NOS: Newcastle-Ottawa Scale
RCT: Randomized Controlled Trial
