# Supplementary material for: Methodological and reporting quality of systematic reviews on health effects of air pollutants were higher than extreme temperatures: a comparative study
Source: BMC Public Health. 2023 Nov 29;23:2371. doi: 10.1186/s12889-023-17256-5 (PMC10687779; doi:10.1186/s12889-023-17256-5)
Supplement: Supplementary file 1 — Supplementary Material 1 [file 12889_2023_17256_MOESM1_ESM.docx]

**
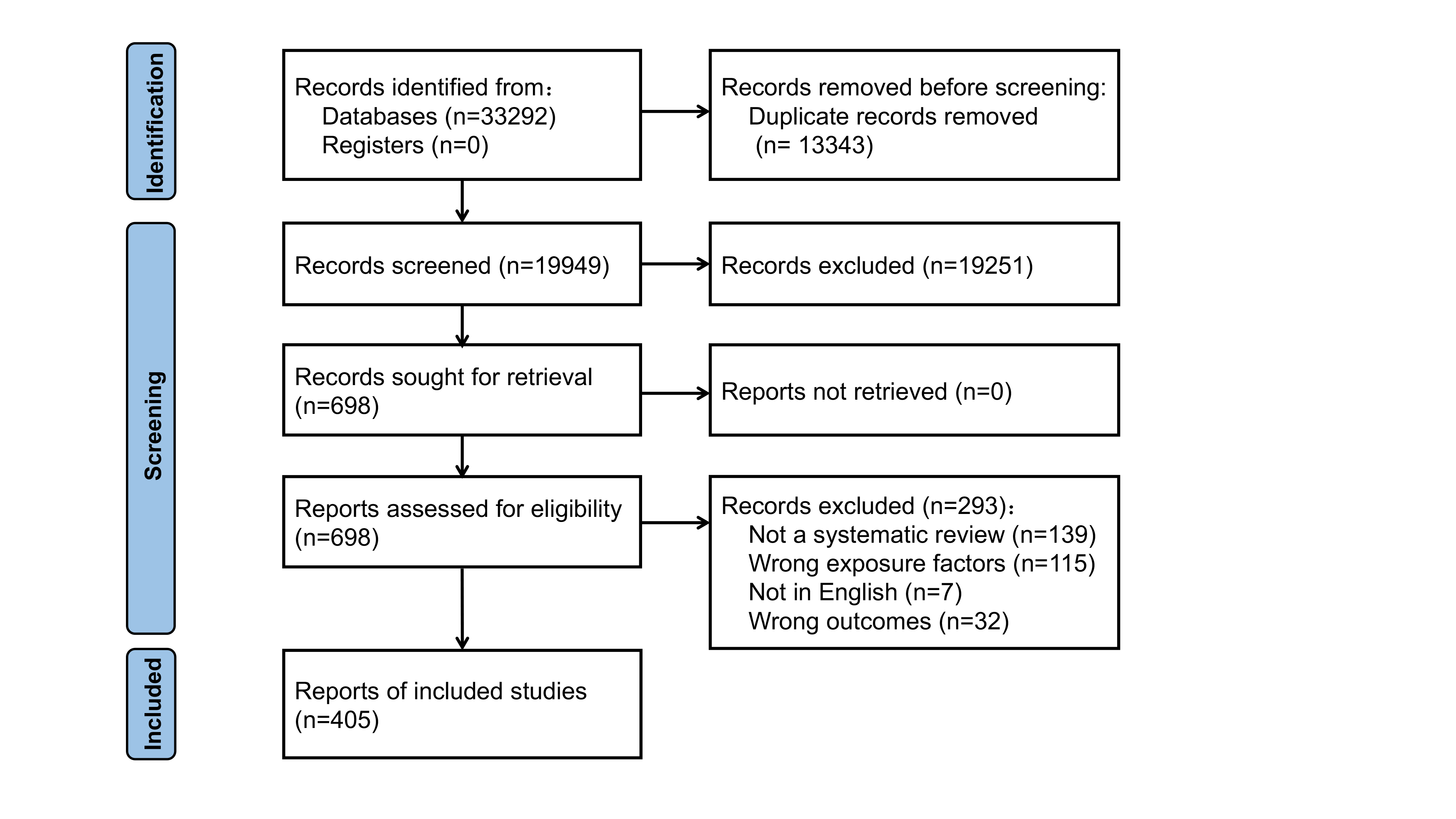
**

**Fig. S1** Flow chart of the literature screening process

**Table S1** The search strategy

**A**

| #1 | "climate change*"/or "temperature*"/or "heat wave*"/or "heatwave*"/or "extreme heat*"/or "cold spell*"/or "extreme cold*".ti,ab. |
| --- | --- |
| #2 | "Climate Change".mh./ or "Temperature".mh. |
| #3 | or/1,2 |
| #4 | "morbidit*"/or "hospitalisation*"/or "hospitalization*"/or "death*"/or "mortalit*"/or "outpatien*"/or "inpatient".ti,ab. |
| #5 | "emergency room*"/or "emergency department*"/or "emergency admi*".ti,ab. |
| #6 | "health effect*".ti,ab. |
| #7 | "Morbidity"/or "Hospitalization"/or "Death"/or "Mortality"/or "Outpatients"/or "Inpatients".mh. |
| #8 | or/4-7 |
| #9 | "systematic* review*"/or "meta analytic*"/or "meta analysis"/or "meta analyses"/or "metaanalysis"/or "metaanalyses"/or "metanalysis"/or "metanalyses"/or "meta synthesis"/or "meta syntheses"/or "meta regression"/or "metaregression".ti,ab. |
| #10 | "Meta-Analysis as Topic".mh. |
| #11 | or/9,10 |
| #12 | "search*"/or "criteri*"/or "database*".ti,ab. |
| #13 | "review*".pt.ti |
| #14 | and/12,13 |
| #15 | "evidence based review*"/or "comprehensive review*"/or "critical review*"/or "quantitative review*"/or "structured review*"/or "integrative review*"/or "realist review*".ti,ab. |
| #16 | or/11,14,15 |
| #17 | and/3,8,16 |

**B**

| #1 | "particulate matter"/or "PM10"/or "PM2.5"/or"PM1"/or "fine particle*"/or "thoracic particle*"/or "ultrafine PM"/or "UFP".ti,ab. |
| --- | --- |
| #2 | "carbon".ti,ab. |
| #3 | "Particulate Matter".mh. |
| #4 | "PM".ti,ab. |
| #5 | or/1,2,3,4 |
| #6 | "EC"/or "BC".tw. |
| #7 | and/5,6 |
| #8 | "black carbon"/or "carbon black"/or "elemental carbon"/or "element carbon".ti,ab. |
| #9 | or/7,8 |
| #10 | "air pollut*"/or "air quality".ti,ab. |
| #11 | "ozone"/or "O3".ti,ab. |
| #12 | "carbon monoxide"/or "CO".ti,ab. |
| #13 | "nitrogen dioxide"/or "NO2".ti,ab. |
| #14 | "sulphur dioxide"/or "SO2".ti,ab. |
| #15 | "Air Pollution".mh. |
| #16 | "Nitrogen Dioxide".mh. |
| #17 | "Sulfur Dioxide".mh. |
| #18 | "Carbon Monoxide".mh. |
| #19 | "Ozone".mh. |
| #20 | or/1,3,9,10,11,12,13,14,15,16,17,18,19 |
| #21 | "systematic review*"/or "meta analy*"/or "metaanaly*"/or "meta regress*"/or "metaregress*".ti,ab. |
| #22 | Systematic Reviews as Topic.mh./or "Systematic Review".pt./or Meta-Analysis as Topic.mh./or "Meta-Analysis".pt. |
| #23 | or/21,22 |
| #24 | search/or criteri*/or database*.ti,ab. |
| #25 | review.pt. |
| #26 | and/24,25 |
| #27 | or/23,26 |
| #28 | "morbidity"/or "hospitalization"/or "hospitalisation"/or "death"/or "mortality"/or "outpatient"/or "emergency room"/or "health effect"/or "emergency admission"/or "inpatient".ti,ab. |
| #29 | "Morbidity".mh. |
| #30 | "Hospitalization".mh. |
| #31 | "Death".mh. |
| #32 | "Mortality".mh. |
| #33 | "Outpatients"/or "Inpatients".mh. |
| #34 | or/29,30,31,32,33 |
| #35 | or/28,34 |
| #36 | and/20,27,35 |

**C**

| #1 | "temperature*" /or "weather*"/or "meteorolog*"/or "heat*"/or "cold*"/or "season*".ti,ab. |
| --- | --- |
| #2 | "Seasons"/or "Climate Change"/or "Weather"/or "Temperature".mh. |
| #3 | or/1,2 |
| #4 | "air pollution*"/or "air pollutant*"/or "air polluted*"/or "air quality*"/or "air contamination*"/or "atmosphere pollutant*"/or "atmosphere pollution*"/or "atmosphere contamination*"/or "atmospheric pollution*"/or "atmospheric pollutant*"/or "atmospheric contamination*"/or "ambient particulate matter*"/or "particulate matter*"/or "particle*".ti,ab. |
| #5 | sulfur dioxide"/or "SO2"/or "nitrogen dioxide"/or "NO2"/or "ozone"/or "O3"/or "carbon monoxide"/or "CO"/or "PM10"/or "PM2.5"/or "PM1".ti,ab. |
| #6 | "Sulfur Dioxide"/or "Nitrogen Dioxide"/or "Carbon Monoxide"/or "Ozone"/or "Particulate Matter"/or "Air Pollution".mh. |
| #7 | or/4,5,6 |
| #8 | "particulate matter"/or "aerosols".sh. |
| #9 | particulate matter*/or "PM10"/or "PM2.5"/or "fine particle*"/or "thoracic particle*"/or "ultrafine"/or "aerosol*"/or "carbon*"/or "soot*"/or "ultrafine PM"/or "UFP".ti,ab. |
| #10 | "PM".tw. |
| #11 | or/8,9,10 |
| #12 | "EC" /or "BC".tw. |
| #13 | and/11,12 |
| #14 | "black carbon*"/or "carbon black*"/or "elemental carbon*"/or "element carbon*".ti,ab. |
| #15 | or/13,14 |
| #16 | or/7,15 |
| #17 | "morbidit*"/or "hospitalisation*"/or "hospitalization*"/or "death*"/or "mortalit*"/or "outpatien*"/or "inpatient"/or "emergency room*"/or "emergency department*"/or "health effect*"/or "emergency admi*".ti,ab. |
| #18 | "Outpatients"/or "Mortality"/or "Death"/or "Hospitalization"/or "Morbidity"/or "Inpatients".mh. |
| #19 | or/17,18 |
| #20 | "modif*"/or "interact*".ti,ab. |
| #21 | "systematic review*" or "meta analy*" or "metaanaly*" or "meta regress*" or "metaregress*".ti,ab. |
| #22 | "Systematic Reviews as Topic".mh. or "Systematic Review".pt. or "Meta-Analysis as Topic".mh. or "Meta-Analysis".pt. |
| #23 | or 21,22 |
| #24 | "search" or "criteri*" or "database*".ti,ab. |
| #25 | review.pt. |
| #26 | and 24,25 |
| #27 | or 23,26 |
| #28 | and 3,16,19,20,27 |

**D**

| #1 | "Greenhouse Gases"[Mesh] |
| --- | --- |
| #2 | (((("Carbon Dioxide"[Mesh]) OR "Methane"[Mesh]) OR "Nitrous Oxide"[Mesh]) OR "Fluorocarbons"[Mesh]) OR "Sulfur Hexafluoride"[Mesh] |
| #3 | ((((((((((((((((greenhouse gas*[Title/Abstract]) OR (Carbon Dioxide[Title/Abstract])) OR (CO2[Title/Abstract])) OR (methane*[Title/Abstract])) OR (CH4[Title/Abstract])) OR (nitrous oxide*[Title/Abstract])) OR (N2O[Title/Abstract])) OR (hydrofluorocarbon*[Title/Abstract])) OR (perfluorochemical[Title/Abstract])) OR (HFC[Title/Abstract])) OR (PFC[Title/Abstract])) OR (perfluorinated organic compound*[Title/Abstract])) OR (Fluorocarbon*[Title/Abstract])) OR (Perfluorocarbon*[Title/Abstract])) OR (sulfur hexafluoride[Title/Abstract])) OR (SF6[Title/Abstract])) OR (GHG[Transliterated Title]) |
| #4 | #1 OR #2 OR #3 |
| #5 | (("Systematic Reviews as Topic"[Mesh]) OR "Systematic Review" [Publication Type]) OR ( "Meta-Analysis" [Publication Type] OR "Meta-Analysis as Topic"[Mesh] ) |
| #6 | ((((systematic review*[Title/Abstract]) OR (meta analy*[Title/Abstract])) OR (metaanaly*[Title/Abstract])) OR (meta regress*[Title/Abstract])) OR (metaregress*[Title/Abstract]) |
| #7 | #5 OR #6 |
| #8 | "Review" [Publication Type] |
| #9 | ((search[Title/Abstract]) OR (criteri*[Title/Abstract])) OR (database*[Title/Abstract]) |
| #10 | #8 AND #9 |
| #11 | #7 OR #10 |
| #12 | (((("Hospitalization"[Mesh]) OR "Morbidity"[Mesh]) OR "Death"[Mesh]) OR "Mortality"[Mesh]) OR "Outpatients"[Mesh] OR "Inpatients"[Mesh] |
| #13 | "morbidity"[Title/Abstract] OR "hospitalization"[Title/Abstract] OR "hospitalisation"[Title/Abstract] OR "death"[Title/Abstract] OR "mortality"[Title/Abstract] OR "outpatient"[Title/Abstract] OR "inpatient"[Title/Abstract] OR "emergency room"[Title/Abstract] OR "health effect"[Title/Abstract] OR "emergency admission"[Title/Abstract] |
| #14 | #12 OR #13 |
| #15 | #4 AND #11 AND #14 |

**Table S2** The results of the AMSTAR 2 checklist assessment

| **AMSTAR 2 checklist** | **Pollution**  **(n=286,%)** | **Temperature**  **(n=108,%)** | **P Value** | **Interaction**  **(n=11,%)** | **Total**  **(n=405,%)** |
| --- | --- | --- | --- | --- | --- |
| 1. Components of PICO question | 286 (100.0) | 108 (100.0) | N.S. | 11 (100.0) | 405 (100.0) |
| 2. Review protocol | 76 (26.6) | 28 (25.9) | N.S. | 2 (18.2) | 106 (26.2) |
| 3. Explanation of study design | 10 (3.5) | 0 | N.S. | 0 | 10 (2.5) |
| 4. Comprehensive literature search strategy | 281 (98.3) | 107 (99.1) | N.S. | 9 (81.8) | 397 (98.0) |
| 5. Study selection in duplicate | 165 (57.7) | 50 (46.3) | * | 6 (54.5) | 221 (54.6) |
| 6. Data extraction in duplicate | 148 (51.7) | 38 (35.2) | ** | 5 (45.5) | 191 (47.2) |
| 7. List of excluded studies and justify the exclusions | 16 (5.6) | 1 (0.9) | N.S. | 0 | 17 (4.2) |
| 8. Study characteristic | 270 (94.4) | 99 (91.7) | N.S. | 8 (72.7) | 377 (93.1) |
| 9. Satisfactory technique for assessing risk of bias | 182 (63.6) | 45 (41.7) | *** | 6 (54.5) | 233 (57.5) |
| 10. Sources of funding for each study | 2 (0.7) | 0 | N.S. | 0 | 2 (0.5) |
| 11. Appropriate methods | 286 (100.0) | 108 (100.0) | N.S. | 11 (100.0) | 405 (100.0) |
| 12. Assess potential impact of risk of bias on the results | 179 (62.6) | 79 (73.1) | * | 8 (72.7) | 266 (65.7) |
| 13. Account for risk of bias when interpreting/discussing | 197 (68.9) | 47 (43.5) | *** | 5 (45.5) | 249 (61.5) |
| 14. Satisfactory explanation for and discussion of any heterogeneity | 199 (69.6) | 49 (45.4) | *** | 4 (36.4) | 252 (62.2) |
| 15. Publication bias (small sample bias) assessed and discussed | 254 (88.8) | 90 (83.3) | N.S. | 9 (81.8) | 353 (87.2) |
| 16. Potential sources of conflict of interest | 265 (92.7) | 98 (90.7) | N.S. | 10 (90.9) | 373 (92.1) |

*** *Abbreviations: N.S. = not statistically significant, * P＜0.05, ** P＜0.01, *** P＜0.001.*

**The results were counted by adding up the numbers of “Yes” and “Partial Yes”.*

**Table S3** The results of the PRISMA checklist assessment

| **PRISMA checklist** | **Pollution**  **(n=286,%)** | **Temperature**  **(n=108,%)** | **P Value** | **Interaction**  **(n=11,%)** | **Total**  **(n=405,%)** |
| --- | --- | --- | --- | --- | --- |
| 1. Title | 257 (89.9) | 83 (76.9) | ** | 10 (90.9) | 350 (86.4) |
| 2. Structured summary | 286 (100.0) | 108 (100.0) | N.S. | 11 (100.0) | 405 (100.0) |
| 3. Rationale | 286 (100.0) | 108 (100.0) | N.S. | 11 (100.0) | 405 (100.0) |
| 4. Objectives | 285 (99.7) | 108 (100.0) | N.S. | 11 (100.0) | 404 (99.8) |
| 5. Protocol and registration | 77 (26.9) | 28 (25.9) | N.S. | 2 (18.2) | 107 (26.4) |
| 6. Eligibility criteria | 275 (96.2) | 6 (98.1) | N.S. | 10 (90.9) | 391 (96.5) |
| 7. Information sources | 286 (100.0) | 108 (100.0) | N.S. | 11 (100.0) | 405 (100.0) |
| 8. Search | 215 (75.2) | 62 (57.4) | ** | 9 (81.8) | 286 (70.6) |
| 9. Study selection | 246 (86.0) | 98 (90.7) | N.S. | 9 (81.8) | 353 (87.2) |
| 10. Data collection process | 236 (82.5) | 78 (72.2) | * | 9 (81.8) | 323 (79.8) |
| 11. Data items | 249 (87.1) | 69 (63.9) | *** | 9 (81.8) | 327 (80.7) |
| 12. Risk of bias in individual studies | 176 (61.5) | 44 (40.7) | *** | 5 (45.5) | 225 (55.6) |
| 13. Summary measures | 221 (77.3) | 51 (47.2) | *** | 6 (54.5) | 278 (68.6) |
| 14. Synthesis of results | 225 (78.7) | 49 (45.4) | *** | 6 (54.5) | 280 (69.1) |
| 15. Risk of bias across studies | 193 (67.5) | 34 (31.5) | *** | 5 (45.5) | 232 (57.3) |
| 16. Additional analyses | 187 (65.4) | 36 (33.3) | *** | 5 (45.5) | 228 (56.3) |
| 17. Study selection | 267 (93.4) | 107 (99.1) | * | 10 (90.9) | 384 (94.8) |
| 18. Study characteristics | 273 (95.5) | 102 (94.4) | N.S. | 8 (72.7) | 383 (94.6) |
| 19. Risk of bias within studies | 153 (53.5) | 37 (34.3) | *** | 4 (36.4) | 194 (47.9) |
| 20. Results of individual studies | 230 (80.4) | 55 (50.9) | *** | 5 (45.5) | 290 (71.6) |
| 21. Synthesis results | 224 (78.3) | 49 (45.4) | *** | 5 (45.5) | 278 (68.6) |
| 22. Risk of bias across studies | 169 (59.1) | 31 (28.7) | *** | 5 (45.5) | 205 (50.6) |
| 23. Additional analysis | 183 (64.0) | 38 (35.2) | *** | 5 (45.5) | 226 (55.8) |
| 24. Summary of evidence | 286 (100.0) | 108 (100.0) | N.S. | 11 (100.0) | 405 (100.0) |
| 25. Limitations | 236 (82.5) | 75 (69.4) | ** | 7 (63.6) | 318 (78.5) |
| 26. Conclusions | 286 (100.0) | 107 (99.1) | N.S. | 11 (100.0) | 404 (99.8) |
| 27. Funding | 249 (87.1) | 90 (83.3) | N.S. | 10 (90.9) | 349 (86.2) |

*** *Abbreviations: N.S. = not statistically significant, * P＜0.05, ** P＜0.01, *** P＜0.001.*

**The results were counted by adding up the numbers of “fully reported” and “partially reported”*

**Table S4** The detailed results of the AMSTAR 2 checklist assessment of all the included studies

|  | Yes |
| --- | --- |
|  | Not Applicable |
|  | Partial Yes |
|  | No |

|  | **1** | **2** | **3** | **4** | **5** | **6** | **7** | **8** | **9** | **10** | **11** | **12** | **13** | **14** | **15** | **16** | **Level** |
| --- | --- | --- | --- | --- | --- | --- | --- | --- | --- | --- | --- | --- | --- | --- | --- | --- | --- |
| Requia et al. (2018) |  |  |  |  |  |  |  |  |  |  |  |  |  |  |  |  | Critically Low |
| Stieb et al. (2020) |  |  |  |  |  |  |  |  |  |  |  |  |  |  |  |  | Low |
| Ren et al. (2017) |  |  |  |  |  |  |  |  |  |  |  |  |  |  |  |  | Critically Low |
| Cui et al. (2015) |  |  |  |  |  |  |  |  |  |  |  |  |  |  |  |  | Critically Low |
| Glinianaia et al. (2004a) |  |  |  |  |  |  |  |  |  |  |  |  |  |  |  |  | Critically Low |
| Kotsyfakis et al. (2019) |  |  |  |  |  |  |  |  |  |  |  |  |  |  |  |  | Critically Low |
| Wong et al. (2004) |  |  |  |  |  |  |  |  |  |  |  |  |  |  |  |  | Critically Low |
| Nhung et al. (2017) |  |  |  |  |  |  |  |  |  |  |  |  |  |  |  |  | Critically Low |
| Vrijheid et al. (2011) |  |  |  |  |  |  |  |  |  |  |  |  |  |  |  |  | Critically Low |
| Pedersen et al. (2014) |  |  |  |  |  |  |  |  |  |  |  |  |  |  |  |  | Critically Low |
| Atkinson et al. (2015) |  |  |  |  |  |  |  |  |  |  |  |  |  |  |  |  | Critically Low |
| Zhang et al. (2016a) |  |  |  |  |  |  |  |  |  |  |  |  |  |  |  |  | Low |
| Ravindra et al. (2020) |  |  |  |  |  |  |  |  |  |  |  |  |  |  |  |  | Low |
| Newell et al. (2017) |  |  |  |  |  |  |  |  |  |  |  |  |  |  |  |  | Moderate |
| Braithwaite et al. (2019) |  |  |  |  |  |  |  |  |  |  |  |  |  |  |  |  | Low |
| Orellano et al. (2017) |  |  |  |  |  |  |  |  |  |  |  |  |  |  |  |  | Low |
| Chen et al. (2014) |  |  |  |  |  |  |  |  |  |  |  |  |  |  |  |  | Critically Low |
| Song et al. (2014) |  |  |  |  |  |  |  |  |  |  |  |  |  |  |  |  | Critically Low |
| Yang et al. (2020) |  |  |  |  |  |  |  |  |  |  |  |  |  |  |  |  | Critically Low |
| Song et al. (2016) |  |  |  |  |  |  |  |  |  |  |  |  |  |  |  |  | Low |
| Shah et al. (2013) |  |  |  |  |  |  |  |  |  |  |  |  |  |  |  |  | Critically Low |
| Newell et al. (2018) |  |  |  |  |  |  |  |  |  |  |  |  |  |  |  |  | Low |
| Kim et al. (2018) |  |  |  |  |  |  |  |  |  |  |  |  |  |  |  |  | Critically Low |
| Kihal et al. (2020) |  |  |  |  |  |  |  |  |  |  |  |  |  |  |  |  | Critically Low |
| Zhao et al. (2017) |  |  |  |  |  |  |  |  |  |  |  |  |  |  |  |  | Critically Low |
| Scheers et al. (2015) |  |  |  |  |  |  |  |  |  |  |  |  |  |  |  |  | Critically Low |
| Siddika et al. (2016) |  |  |  |  |  |  |  |  |  |  |  |  |  |  |  |  | Critically Low |
| Liu et al. (2020) |  |  |  |  |  |  |  |  |  |  |  |  |  |  |  |  | Critically Low |
| Shah et al. (2015) |  |  |  |  |  |  |  |  |  |  |  |  |  |  |  |  | Low |
| Chang et al. (2015) |  |  |  |  |  |  |  |  |  |  |  |  |  |  |  |  | Critically Low |
| Shang et al. (2013) |  |  |  |  |  |  |  |  |  |  |  |  |  |  |  |  | Critically Low |
| Li et al. (2014) |  |  |  |  |  |  |  |  |  |  |  |  |  |  |  |  | Critically Low |
| Yang et al. (2016) |  |  |  |  |  |  |  |  |  |  |  |  |  |  |  |  | Critically Low |
| Bekkar et al. (2020) |  |  |  |  |  |  |  |  |  |  |  |  |  |  |  |  | Critically Low |
| Yang et al. (2014) |  |  |  |  |  |  |  |  |  |  |  |  |  |  |  |  | Critically Low |
| Lai et al. (2013) |  |  |  |  |  |  |  |  |  |  |  |  |  |  |  |  | Critically Low |
| Huangfu et al. (2020) |  |  |  |  |  |  |  |  |  |  |  |  |  |  |  |  | High |
| DeVries et al. (2017) |  |  |  |  |  |  |  |  |  |  |  |  |  |  |  |  | Critically Low |
| Vodonos et al. (2018) |  |  |  |  |  |  |  |  |  |  |  |  |  |  |  |  | Critically Low |
| Yu et al. (2014) |  |  |  |  |  |  |  |  |  |  |  |  |  |  |  |  | Critically Low |
| Gao et al. (2020) |  |  |  |  |  |  |  |  |  |  |  |  |  |  |  |  | Critically Low |
| Wang et al. (2019) |  |  |  |  |  |  |  |  |  |  |  |  |  |  |  |  | Critically Low |
| Wang et al. (2014a) |  |  |  |  |  |  |  |  |  |  |  |  |  |  |  |  | Critically Low |
| Chen et al. (2020) |  |  |  |  |  |  |  |  |  |  |  |  |  |  |  |  | High |
| Mills et al. (2016) |  |  |  |  |  |  |  |  |  |  |  |  |  |  |  |  | Critically Low |
| Zhu et al. (2013) |  |  |  |  |  |  |  |  |  |  |  |  |  |  |  |  | Critically Low |
| Bell et al. (2013) |  |  |  |  |  |  |  |  |  |  |  |  |  |  |  |  | Critically Low |
| Sakhvidi et al. (2020) |  |  |  |  |  |  |  |  |  |  |  |  |  |  |  |  | Critically Low |
| Achilleos et al. (2017) |  |  |  |  |  |  |  |  |  |  |  |  |  |  |  |  | Low |
| Hoek et al. (2013) |  |  |  |  |  |  |  |  |  |  |  |  |  |  |  |  | Critically Low |
| Lee et al. (2020) |  |  |  |  |  |  |  |  |  |  |  |  |  |  |  |  | Low |
| Zhu et al. (2020) |  |  |  |  |  |  |  |  |  |  |  |  |  |  |  |  | Critically Low |
| Pranata et al. (2020) |  |  |  |  |  |  |  |  |  |  |  |  |  |  |  |  | Critically Low |
| Hamra et al. (2014) |  |  |  |  |  |  |  |  |  |  |  |  |  |  |  |  | Critically Low |
| Bell et al. (2014) |  |  |  |  |  |  |  |  |  |  |  |  |  |  |  |  | Critically Low |
| Zhao et al. (2018) |  |  |  |  |  |  |  |  |  |  |  |  |  |  |  |  | Critically Low |
| Fu et al. (2019) |  |  |  |  |  |  |  |  |  |  |  |  |  |  |  |  | Critically Low |
| Yan et al. (2013) |  |  |  |  |  |  |  |  |  |  |  |  |  |  |  |  | Critically Low |
| Zheng et al. (2015) |  |  |  |  |  |  |  |  |  |  |  |  |  |  |  |  | Critically Low |
| Zhang et al. (2018) |  |  |  |  |  |  |  |  |  |  |  |  |  |  |  |  | Critically Low |
| Ito et al. (2005) |  |  |  |  |  |  |  |  |  |  |  |  |  |  |  |  | Critically Low |
| Cai et al. (2016) |  |  |  |  |  |  |  |  |  |  |  |  |  |  |  |  | Critically Low |
| Sun et al. (2017) |  |  |  |  |  |  |  |  |  |  |  |  |  |  |  |  | Critically Low |
| Park et al. (2013) |  |  |  |  |  |  |  |  |  |  |  |  |  |  |  |  | Critically Low |
| Li et al. (2016a) |  |  |  |  |  |  |  |  |  |  |  |  |  |  |  |  | Critically Low |
| Yuan et al. (2019) |  |  |  |  |  |  |  |  |  |  |  |  |  |  |  |  | Critically Low |
| Mills et al. (2015) |  |  |  |  |  |  |  |  |  |  |  |  |  |  |  |  | Critically Low |
| Huang et al. (2017) |  |  |  |  |  |  |  |  |  |  |  |  |  |  |  |  | Critically Low |
| Lu et al. (2015) |  |  |  |  |  |  |  |  |  |  |  |  |  |  |  |  | Critically Low |
| Orellano et al. (2020) |  |  |  |  |  |  |  |  |  |  |  |  |  |  |  |  | Low |
| Atkinson et al. (2014) |  |  |  |  |  |  |  |  |  |  |  |  |  |  |  |  | Critically Low |
| Yang et al. (2019a) |  |  |  |  |  |  |  |  |  |  |  |  |  |  |  |  | Critically Low |
| Liu et al. (2018) |  |  |  |  |  |  |  |  |  |  |  |  |  |  |  |  | Critically Low |
| Atkinson et al. (2018) |  |  |  |  |  |  |  |  |  |  |  |  |  |  |  |  | Critically Low |
| Glinianaia et al. (2004b) |  |  |  |  |  |  |  |  |  |  |  |  |  |  |  |  | Critically Low |
| Atkinson et al. (2016) |  |  |  |  |  |  |  |  |  |  |  |  |  |  |  |  | Critically Low |
| Faustini et al. (2014) |  |  |  |  |  |  |  |  |  |  |  |  |  |  |  |  | Critically Low |
| Stieb et al. (2002) |  |  |  |  |  |  |  |  |  |  |  |  |  |  |  |  | Critically Low |
| Zhang et al. (2019) |  |  |  |  |  |  |  |  |  |  |  |  |  |  |  |  | Critically Low |
| Ab Manan et al. (2018) |  |  |  |  |  |  |  |  |  |  |  |  |  |  |  |  | Critically Low |
| Guo et al. (2020) |  |  |  |  |  |  |  |  |  |  |  |  |  |  |  |  | Critically Low |
| Luben et al. (2017) |  |  |  |  |  |  |  |  |  |  |  |  |  |  |  |  | Critically Low |
| Koranteng et al. (2007) |  |  |  |  |  |  |  |  |  |  |  |  |  |  |  |  | Critically Low |
| Yang et al. (2019b) |  |  |  |  |  |  |  |  |  |  |  |  |  |  |  |  | Critically Low |
| Jacobs et al. (2017) |  |  |  |  |  |  |  |  |  |  |  |  |  |  |  |  | Critically Low |
| Jaganathan et al. (2019) |  |  |  |  |  |  |  |  |  |  |  |  |  |  |  |  | Critically Low |
| Wu et al. (2018) |  |  |  |  |  |  |  |  |  |  |  |  |  |  |  |  | Critically Low |
| Moore et al. (2016) |  |  |  |  |  |  |  |  |  |  |  |  |  |  |  |  | Critically Low |
| Goldberg et al. (2008) |  |  |  |  |  |  |  |  |  |  |  |  |  |  |  |  | Critically Low |
| King et al. (2018) |  |  |  |  |  |  |  |  |  |  |  |  |  |  |  |  | Moderate |
| Bazyar et al. (2019) |  |  |  |  |  |  |  |  |  |  |  |  |  |  |  |  | Critically Low |
| Yamamoto et al. (2014) |  |  |  |  |  |  |  |  |  |  |  |  |  |  |  |  | Critically Low |
| Levy et al. (2005) |  |  |  |  |  |  |  |  |  |  |  |  |  |  |  |  | Critically Low |
| Copat et al. (2020) |  |  |  |  |  |  |  |  |  |  |  |  |  |  |  |  | Critically Low |
| Rajak et al. (2020) |  |  |  |  |  |  |  |  |  |  |  |  |  |  |  |  | Critically Low |
| Teng et al. (2014) |  |  |  |  |  |  |  |  |  |  |  |  |  |  |  |  | Critically Low |
| Jilani et al. (2020) |  |  |  |  |  |  |  |  |  |  |  |  |  |  |  |  | Critically Low |
| Volk et al. (2020) |  |  |  |  |  |  |  |  |  |  |  |  |  |  |  |  | Critically Low |
| Abdo et al. (2016) |  |  |  |  |  |  |  |  |  |  |  |  |  |  |  |  | Critically Low |
| Popovic et al. (2019) |  |  |  |  |  |  |  |  |  |  |  |  |  |  |  |  | Critically Low |
| Buoli et al. (2018) |  |  |  |  |  |  |  |  |  |  |  |  |  |  |  |  | Critically Low |
| Li et al. (2019) |  |  |  |  |  |  |  |  |  |  |  |  |  |  |  |  | Critically Low |
| Asmus et al. (2016) |  |  |  |  |  |  |  |  |  |  |  |  |  |  |  |  | Critically Low |
| Liu et al. (2016) |  |  |  |  |  |  |  |  |  |  |  |  |  |  |  |  | Low |
| Latza et al. (2009) |  |  |  |  |  |  |  |  |  |  |  |  |  |  |  |  | Critically Low |
| Porpora et al. (2019) |  |  |  |  |  |  |  |  |  |  |  |  |  |  |  |  | Critically Low |
| Wyzga et al. (2015) |  |  |  |  |  |  |  |  |  |  |  |  |  |  |  |  | Critically Low |
| Wang et al. (2018) |  |  |  |  |  |  |  |  |  |  |  |  |  |  |  |  | Critically Low |
| Nasser et al. (2015) |  |  |  |  |  |  |  |  |  |  |  |  |  |  |  |  | Critically Low |
| Luben et al. (2018) |  |  |  |  |  |  |  |  |  |  |  |  |  |  |  |  | Critically Low |
| Pelucchi et al. (2009) |  |  |  |  |  |  |  |  |  |  |  |  |  |  |  |  | Critically Low |
| Maitre et al. (2006) |  |  |  |  |  |  |  |  |  |  |  |  |  |  |  |  | Critically Low |
| Mustafić et al. (2012) |  |  |  |  |  |  |  |  |  |  |  |  |  |  |  |  | Critically Low |
| Mehta et al. (2013) |  |  |  |  |  |  |  |  |  |  |  |  |  |  |  |  | Critically Low |
| Wang et al. (2014b) |  |  |  |  |  |  |  |  |  |  |  |  |  |  |  |  | Critically Low |
| Ji et al. (2011) |  |  |  |  |  |  |  |  |  |  |  |  |  |  |  |  | Critically Low |
| Li et al. (2016b) |  |  |  |  |  |  |  |  |  |  |  |  |  |  |  |  | Critically Low |
| Morris et al. (2001) |  |  |  |  |  |  |  |  |  |  |  |  |  |  |  |  | Critically Low |
| Janghorbani et al. (2014) |  |  |  |  |  |  |  |  |  |  |  |  |  |  |  |  | Critically Low |
| Atkinson et al. (2012) |  |  |  |  |  |  |  |  |  |  |  |  |  |  |  |  | Critically Low |
| Zhang et al. (2016b) |  |  |  |  |  |  |  |  |  |  |  |  |  |  |  |  | Critically Low |
| Luo et al. (2015) |  |  |  |  |  |  |  |  |  |  |  |  |  |  |  |  | Critically Low |
| Peters et al. (2019) |  |  |  |  |  |  |  |  |  |  |  |  |  |  |  |  | Critically Low |
| Franchini et al. (2016) |  |  |  |  |  |  |  |  |  |  |  |  |  |  |  |  | Critically Low |
| Akintoye et al. (2016) |  |  |  |  |  |  |  |  |  |  |  |  |  |  |  |  | Critically Low |
| Li et al. (2012) |  |  |  |  |  |  |  |  |  |  |  |  |  |  |  |  | Critically Low |
| Tsai et al. (2019) |  |  |  |  |  |  |  |  |  |  |  |  |  |  |  |  | Critically Low |
| Spirić et al. (2012) |  |  |  |  |  |  |  |  |  |  |  |  |  |  |  |  | Critically Low |
| Luong et al. (2019) |  |  |  |  |  |  |  |  |  |  |  |  |  |  |  |  | Critically Low |
| Kim et al. (2020) |  |  |  |  |  |  |  |  |  |  |  |  |  |  |  |  | Critically Low |
| Sun et al. (2020) |  |  |  |  |  |  |  |  |  |  |  |  |  |  |  |  | Critically Low |
| Chen et al. (2019) |  |  |  |  |  |  |  |  |  |  |  |  |  |  |  |  | Critically Low |
| Sharma et al. (2020) |  |  |  |  |  |  |  |  |  |  |  |  |  |  |  |  | Critically Low |
| Vieira et al. (2015) |  |  |  |  |  |  |  |  |  |  |  |  |  |  |  |  | Critically Low |
| Bell et al. (2005) |  |  |  |  |  |  |  |  |  |  |  |  |  |  |  |  | Critically Low |
| Kan et al. (2005) |  |  |  |  |  |  |  |  |  |  |  |  |  |  |  |  | Critically Low |
| Conforti et al. (2018) |  |  |  |  |  |  |  |  |  |  |  |  |  |  |  |  | Critically Low |
| Keramatinia et al. (2016) |  |  |  |  |  |  |  |  |  |  |  |  |  |  |  |  | Critically Low |
| Cheng et al. (2019) |  |  |  |  |  |  |  |  |  |  |  |  |  |  |  |  | Critically Low |
| Green et al. (2019) |  |  |  |  |  |  |  |  |  |  |  |  |  |  |  |  | Critically Low |
| van Steen et al. (2019) |  |  |  |  |  |  |  |  |  |  |  |  |  |  |  |  | Critically Low |
| Sun et al. (2018) |  |  |  |  |  |  |  |  |  |  |  |  |  |  |  |  | Critically Low |
| Campbell et al. (2018) |  |  |  |  |  |  |  |  |  |  |  |  |  |  |  |  | Critically Low |
| Odame et al. (2018) |  |  |  |  |  |  |  |  |  |  |  |  |  |  |  |  | Critically Low |
| Ghanizadeh et al. (2017) |  |  |  |  |  |  |  |  |  |  |  |  |  |  |  |  | Critically Low |
| Kuehn, L. and S. McCormick (2017) |  |  |  |  |  |  |  |  |  |  |  |  |  |  |  |  | Critically Low |
| Zhang et al. (2017) |  |  |  |  |  |  |  |  |  |  |  |  |  |  |  |  | Critically Low |
| Philipsborn et al. (2016) |  |  |  |  |  |  |  |  |  |  |  |  |  |  |  |  | Critically Low |
| Asadgol et al. (2020) |  |  |  |  |  |  |  |  |  |  |  |  |  |  |  |  | Critically Low |
| Bai et al. (2019) |  |  |  |  |  |  |  |  |  |  |  |  |  |  |  |  | Critically Low |
| Lu et al. (2018) |  |  |  |  |  |  |  |  |  |  |  |  |  |  |  |  | Critically Low |
| Coates et al. (2019) |  |  |  |  |  |  |  |  |  |  |  |  |  |  |  |  | Critically Low |
| Shi et al. (2019) |  |  |  |  |  |  |  |  |  |  |  |  |  |  |  |  | Critically Low |
| Son et al. (2019) |  |  |  |  |  |  |  |  |  |  |  |  |  |  |  |  | Critically Low |
| Gao et al. (2019) |  |  |  |  |  |  |  |  |  |  |  |  |  |  |  |  | Critically Low |
| Salve et al. (2018) |  |  |  |  |  |  |  |  |  |  |  |  |  |  |  |  | Critically Low |
| Chersich et al. (2018) |  |  |  |  |  |  |  |  |  |  |  |  |  |  |  |  | Critically Low |
| Cheng et al. (2018) |  |  |  |  |  |  |  |  |  |  |  |  |  |  |  |  | Critically Low |
| Ghazani et al. (2018) |  |  |  |  |  |  |  |  |  |  |  |  |  |  |  |  | Critically Low |
| Chan et al. (2019) |  |  |  |  |  |  |  |  |  |  |  |  |  |  |  |  | Critically Low |
| Zanobetti and O'Neill (2018) |  |  |  |  |  |  |  |  |  |  |  |  |  |  |  |  | Critically Low |
| Ma et al. (2020) |  |  |  |  |  |  |  |  |  |  |  |  |  |  |  |  | Critically Low |
| Chersich et al. (2020) |  |  |  |  |  |  |  |  |  |  |  |  |  |  |  |  | Critically Low |
| Chang et al. (2020) |  |  |  |  |  |  |  |  |  |  |  |  |  |  |  |  | Critically Low |
| Cheng et al. (2019) |  |  |  |  |  |  |  |  |  |  |  |  |  |  |  |  | Critically Low |
| Luo et al. (2019) |  |  |  |  |  |  |  |  |  |  |  |  |  |  |  |  | Critically Low |
| Bodaghkhani et al. (2019) |  |  |  |  |  |  |  |  |  |  |  |  |  |  |  |  | Critically Low |
| Thompson et al. (2018) |  |  |  |  |  |  |  |  |  |  |  |  |  |  |  |  | Critically Low |
| Schinasi et al. (2018) |  |  |  |  |  |  |  |  |  |  |  |  |  |  |  |  | Critically Low |
| Leyva et al. (2017) |  |  |  |  |  |  |  |  |  |  |  |  |  |  |  |  | Critically Low |
| Geraghty et al. (2017) |  |  |  |  |  |  |  |  |  |  |  |  |  |  |  |  | Critically Low |
| Moghadamnia et al. (2017) |  |  |  |  |  |  |  |  |  |  |  |  |  |  |  |  | Critically Low |
| Mousavi et al. (2020) |  |  |  |  |  |  |  |  |  |  |  |  |  |  |  |  | Critically Low |
| Arbuthnott et al. (2016) |  |  |  |  |  |  |  |  |  |  |  |  |  |  |  |  | Critically Low |
| Heidari et al. (2020) |  |  |  |  |  |  |  |  |  |  |  |  |  |  |  |  | Critically Low |
| Ainita et al. (2018) |  |  |  |  |  |  |  |  |  |  |  |  |  |  |  |  | Critically Low |
| Otte im Kampe et al. (2016) |  |  |  |  |  |  |  |  |  |  |  |  |  |  |  |  | Critically Low |
| Amegah et al. (2016) |  |  |  |  |  |  |  |  |  |  |  |  |  |  |  |  | Critically Low |
| Astrom et al. (2011) |  |  |  |  |  |  |  |  |  |  |  |  |  |  |  |  | Critically Low |
| Basu (2009 |  |  |  |  |  |  |  |  |  |  |  |  |  |  |  |  | Critically Low |
| Martiello and Giacchi (2010) |  |  |  |  |  |  |  |  |  |  |  |  |  |  |  |  | Critically Low |
| Witt et al. (2015) |  |  |  |  |  |  |  |  |  |  |  |  |  |  |  |  | Critically Low |
| Turner et al. (2012) |  |  |  |  |  |  |  |  |  |  |  |  |  |  |  |  | Critically Low |
| Ye et al. (2012) |  |  |  |  |  |  |  |  |  |  |  |  |  |  |  |  | Critically Low |
| Ryti et al. (2016) |  |  |  |  |  |  |  |  |  |  |  |  |  |  |  |  | Critically Low |
| Basu and Samet (2002) |  |  |  |  |  |  |  |  |  |  |  |  |  |  |  |  | Critically Low |
| Bhaskaran et al. (2009) |  |  |  |  |  |  |  |  |  |  |  |  |  |  |  |  | Critically Low |
| Carlton et al. (2016) |  |  |  |  |  |  |  |  |  |  |  |  |  |  |  |  | Critically Low |
| Beltran et al. (2013) |  |  |  |  |  |  |  |  |  |  |  |  |  |  |  |  | Critically Low |
| Fan et al. (2014) |  |  |  |  |  |  |  |  |  |  |  |  |  |  |  |  | Critically Low |
| Li et al. (2015) |  |  |  |  |  |  |  |  |  |  |  |  |  |  |  |  | Critically Low |
| Lian et al. (2015) |  |  |  |  |  |  |  |  |  |  |  |  |  |  |  |  | Critically Low |
| Bunker et al. (2016) |  |  |  |  |  |  |  |  |  |  |  |  |  |  |  |  | Critically Low |
| Xu et al. (2016) |  |  |  |  |  |  |  |  |  |  |  |  |  |  |  |  | Critically Low |
| Strand et al. (2011) |  |  |  |  |  |  |  |  |  |  |  |  |  |  |  |  | Critically Low |
| Xu et al. (2012) |  |  |  |  |  |  |  |  |  |  |  |  |  |  |  |  | Critically Low |
| Carolan-Olah and Frankowska (2014) |  |  |  |  |  |  |  |  |  |  |  |  |  |  |  |  | Critically Low |
| Phung et al. (2016). |  |  |  |  |  |  |  |  |  |  |  |  |  |  |  |  | Critically Low |
| Ramesh et al. (2013) |  |  |  |  |  |  |  |  |  |  |  |  |  |  |  |  | Critically Low |
| Yu et al. (2012) |  |  |  |  |  |  |  |  |  |  |  |  |  |  |  |  | Critically Low |
| Xu et al. (2014) |  |  |  |  |  |  |  |  |  |  |  |  |  |  |  |  | Critically Low |
| Cheng et al. (2014) |  |  |  |  |  |  |  |  |  |  |  |  |  |  |  |  | Critically Low |
| VianaI and Ignotti (2013) |  |  |  |  |  |  |  |  |  |  |  |  |  |  |  |  | Critically Low |
| Burkart et al. (2014) |  |  |  |  |  |  |  |  |  |  |  |  |  |  |  |  | Critically Low |
| Poursafa et al. (2015) |  |  |  |  |  |  |  |  |  |  |  |  |  |  |  |  | Critically Low |
| Anenberg et al. (2020) |  |  |  |  |  |  |  |  |  |  |  |  |  |  |  |  | Critically Low |
| Chen et al. (2017) |  |  |  |  |  |  |  |  |  |  |  |  |  |  |  |  | Critically Low |
| Li et al. (2017) |  |  |  |  |  |  |  |  |  |  |  |  |  |  |  |  | Critically Low |
| Zang et al. (2022) |  |  |  |  |  |  |  |  |  |  |  |  |  |  |  |  | Low |
| Yee et al. (2021) |  |  |  |  |  |  |  |  |  |  |  |  |  |  |  |  | Critically Low |
| Niu et al. (2021) |  |  |  |  |  |  |  |  |  |  |  |  |  |  |  |  | Critically Low |
| Katoto et al. (2021) |  |  |  |  |  |  |  |  |  |  |  |  |  |  |  |  | Critically Low |
| Hu et al. (2022) |  |  |  |  |  |  |  |  |  |  |  |  |  |  |  |  | Critically Low |
| Zheng et al. (2021) |  |  |  |  |  |  |  |  |  |  |  |  |  |  |  |  | Critically Low |
| Liu et al. (2021） |  |  |  |  |  |  |  |  |  |  |  |  |  |  |  |  | Critically Low |
| Walter et al. (2021) |  |  |  |  |  |  |  |  |  |  |  |  |  |  |  |  | Critically Low |
| Yu et al. (2021) |  |  |  |  |  |  |  |  |  |  |  |  |  |  |  |  | Critically Low |
| Huang et al. (2021) |  |  |  |  |  |  |  |  |  |  |  |  |  |  |  |  | Low |
| Davoudi et al. (2021) |  |  |  |  |  |  |  |  |  |  |  |  |  |  |  |  | Low |
| Zhang et al. (2021). |  |  |  |  |  |  |  |  |  |  |  |  |  |  |  |  | Critically Low |
| Ciabattini et al. (2021) |  |  |  |  |  |  |  |  |  |  |  |  |  |  |  |  | Critically Low |
| Orellano et al. (2021) |  |  |  |  |  |  |  |  |  |  |  |  |  |  |  |  | Moderate |
| Zheng et al. (2021) |  |  |  |  |  |  |  |  |  |  |  |  |  |  |  |  | Moderate |
| Maleki et al. (2021) |  |  |  |  |  |  |  |  |  |  |  |  |  |  |  |  | Critically Low |
| Zhu et al. (2021) |  |  |  |  |  |  |  |  |  |  |  |  |  |  |  |  | Critically Low |
| Park et al. (2021) |  |  |  |  |  |  |  |  |  |  |  |  |  |  |  |  | Moderate |
| Stieb et al. (2021) |  |  |  |  |  |  |  |  |  |  |  |  |  |  |  |  | Low |
| Alexeeff et al. (2021) |  |  |  |  |  |  |  |  |  |  |  |  |  |  |  |  | Critically Low |
| Prueitt et al. (2022) |  |  |  |  |  |  |  |  |  |  |  |  |  |  |  |  | Critically Low |
| MEO et al. (2021) |  |  |  |  |  |  |  |  |  |  |  |  |  |  |  |  | Critically Low |
| Zhang et al. (2021) |  |  |  |  |  |  |  |  |  |  |  |  |  |  |  |  | Moderate |
| Ning et al. (2021) |  |  |  |  |  |  |  |  |  |  |  |  |  |  |  |  | Critically Low |
| Xiang et al. (2021) |  |  |  |  |  |  |  |  |  |  |  |  |  |  |  |  | Critically Low |
| Zou et al. (2021) |  |  |  |  |  |  |  |  |  |  |  |  |  |  |  |  | Critically Low |
| Trushna et al. (2021) |  |  |  |  |  |  |  |  |  |  |  |  |  |  |  |  | Low |
| Chen et al. (2021) |  |  |  |  |  |  |  |  |  |  |  |  |  |  |  |  | Critically Low |
| Zhao et al. (2021) |  |  |  |  |  |  |  |  |  |  |  |  |  |  |  |  | Low |
| Ma et al. (2021). |  |  |  |  |  |  |  |  |  |  |  |  |  |  |  |  | Critically Low |
| Yue et al. (2021) |  |  |  |  |  |  |  |  |  |  |  |  |  |  |  |  | Critically Low |
| Noorimotlagh et al. (2021) |  |  |  |  |  |  |  |  |  |  |  |  |  |  |  |  | Critically Low |
| Ibrahim et al. (2021) |  |  |  |  |  |  |  |  |  |  |  |  |  |  |  |  | Critically Low |
| Lederer et al. (2021) |  |  |  |  |  |  |  |  |  |  |  |  |  |  |  |  | Critically Low |
| Ghosh et al. (2021) |  |  |  |  |  |  |  |  |  |  |  |  |  |  |  |  | Low |
| Shahrbaf et al. (2021) |  |  |  |  |  |  |  |  |  |  |  |  |  |  |  |  | Critically Low |
| Bernardini et al. (2020) |  |  |  |  |  |  |  |  |  |  |  |  |  |  |  |  | Critically Low |
| Xie et al. (2021) |  |  |  |  |  |  |  |  |  |  |  |  |  |  |  |  | Low |
| Lin et al. (2021) |  |  |  |  |  |  |  |  |  |  |  |  |  |  |  |  | Critically Low |
| Ma et al. (2021） |  |  |  |  |  |  |  |  |  |  |  |  |  |  |  |  | Critically Low |
| Wang et al. (2021) |  |  |  |  |  |  |  |  |  |  |  |  |  |  |  |  | Critically Low |
| Wang et al. (2021) |  |  |  |  |  |  |  |  |  |  |  |  |  |  |  |  | Critically Low |
| Yu et al. (2021) |  |  |  |  |  |  |  |  |  |  |  |  |  |  |  |  | Critically Low |
| Wu et al. (2022) |  |  |  |  |  |  |  |  |  |  |  |  |  |  |  |  | Critically Low |
| Uwak et al. (2021) |  |  |  |  |  |  |  |  |  |  |  |  |  |  |  |  | Low |
| Farhadi et al. (2020) |  |  |  |  |  |  |  |  |  |  |  |  |  |  |  |  | Low |
| Sun et al. (2020) |  |  |  |  |  |  |  |  |  |  |  |  |  |  |  |  | Critically Low |
| Bai et al. (2020) |  |  |  |  |  |  |  |  |  |  |  |  |  |  |  |  | Critically Low |
| Kim et al. (2020) |  |  |  |  |  |  |  |  |  |  |  |  |  |  |  |  | Critically Low |
| Harari et al. (2020) |  |  |  |  |  |  |  |  |  |  |  |  |  |  |  |  | Critically Low |
| Abed Al Ahad et al. (2020) |  |  |  |  |  |  |  |  |  |  |  |  |  |  |  |  | Critically Low |
| Villeneuve et al. (2020) |  |  |  |  |  |  |  |  |  |  |  |  |  |  |  |  | Critically Low |
| Wang et al. (2020). |  |  |  |  |  |  |  |  |  |  |  |  |  |  |  |  | Critically Low |
| Yang et al. (2020) |  |  |  |  |  |  |  |  |  |  |  |  |  |  |  |  | Critically Low |
| Amiri et al. (2021) |  |  |  |  |  |  |  |  |  |  |  |  |  |  |  |  | Critically Low |
| Dimitrova et al. (2021) |  |  |  |  |  |  |  |  |  |  |  |  |  |  |  |  | Critically Low |
| Kakaei et al. (2021) |  |  |  |  |  |  |  |  |  |  |  |  |  |  |  |  | Critically Low |
| Frangione et al. (2022) |  |  |  |  |  |  |  |  |  |  |  |  |  |  |  |  | Critically Low |
| Heo et al. (2021) |  |  |  |  |  |  |  |  |  |  |  |  |  |  |  |  | Critically Low |
| Liu et al. (2021) |  |  |  |  |  |  |  |  |  |  |  |  |  |  |  |  | Low |
| Ray et al. (2021) |  |  |  |  |  |  |  |  |  |  |  |  |  |  |  |  | Critically Low |
| Moon (2021) |  |  |  |  |  |  |  |  |  |  |  |  |  |  |  |  | Critically Low |
| Romero Starke et al. (2021) |  |  |  |  |  |  |  |  |  |  |  |  |  |  |  |  | Low |
| Sexton et al. (2021) |  |  |  |  |  |  |  |  |  |  |  |  |  |  |  |  | Low |
| Song et al. (2021) |  |  |  |  |  |  |  |  |  |  |  |  |  |  |  |  | Critically Low |
| Weilnhammer et al. (2021) |  |  |  |  |  |  |  |  |  |  |  |  |  |  |  |  | Critically Low |
| Zafeiratou et al. (2021) |  |  |  |  |  |  |  |  |  |  |  |  |  |  |  |  | Critically Low |
| Liang et al. (2021) |  |  |  |  |  |  |  |  |  |  |  |  |  |  |  |  | Low |
| Li et al. (2020) |  |  |  |  |  |  |  |  |  |  |  |  |  |  |  |  | Critically Low |
| Grigorieva and Lukyanets (2021) |  |  |  |  |  |  |  |  |  |  |  |  |  |  |  |  | Critically Low |
| Areal et al. (2022) |  |  |  |  |  |  |  |  |  |  |  |  |  |  |  |  | Critically Low |
| Song et al. (2022) |  |  |  |  |  |  |  |  |  |  |  |  |  |  |  |  | Low |
| Chandra et al. (2022) |  |  |  |  |  |  |  |  |  |  |  |  |  |  |  |  | Critically Low |
| Huang et al. (2022) |  |  |  |  |  |  |  |  |  |  |  |  |  |  |  |  | Critically Low |
| Smaller et al. (2022) |  |  |  |  |  |  |  |  |  |  |  |  |  |  |  |  | Critically Low |
| Yu et al. (2022) |  |  |  |  |  |  |  |  |  |  |  |  |  |  |  |  | Critically Low |
| Hu et al. (2022) |  |  |  |  |  |  |  |  |  |  |  |  |  |  |  |  | Critically Low |
| Ziou et al. (2022) |  |  |  |  |  |  |  |  |  |  |  |  |  |  |  |  | Low |
| Zhe Sun et al. (2022) |  |  |  |  |  |  |  |  |  |  |  |  |  |  |  |  | Low |
| Markozannes et al. (2022) |  |  |  |  |  |  |  |  |  |  |  |  |  |  |  |  | Critically Low |
| Liu et al. (2022) |  |  |  |  |  |  |  |  |  |  |  |  |  |  |  |  | Low |
| Perry et al. (2022) |  |  |  |  |  |  |  |  |  |  |  |  |  |  |  |  | Critically Low |
| Jia et al. (2022) |  |  |  |  |  |  |  |  |  |  |  |  |  |  |  |  | Critically Low |
| Wang et al. (2022) |  |  |  |  |  |  |  |  |  |  |  |  |  |  |  |  | Critically Low |
| Bont et al. (2022) |  |  |  |  |  |  |  |  |  |  |  |  |  |  |  |  | Low |
| Chen et al. (2022) |  |  |  |  |  |  |  |  |  |  |  |  |  |  |  |  | Critically Low |
| Li et al. (2022) |  |  |  |  |  |  |  |  |  |  |  |  |  |  |  |  | Low |
| Heo et al. (2022) |  |  |  |  |  |  |  |  |  |  |  |  |  |  |  |  | Low |
| Pritchett et al. (2022) |  |  |  |  |  |  |  |  |  |  |  |  |  |  |  |  | Low |
| Zhang et al. (2022) |  |  |  |  |  |  |  |  |  |  |  |  |  |  |  |  | Critically Low |
| Lin et al. (2022) |  |  |  |  |  |  |  |  |  |  |  |  |  |  |  |  | Critically Low |
| Guo et al. (2022) |  |  |  |  |  |  |  |  |  |  |  |  |  |  |  |  | Critically Low |
| Yang et al. (2022) |  |  |  |  |  |  |  |  |  |  |  |  |  |  |  |  | Critically Low |
| Khosravipour et al. (2022) |  |  |  |  |  |  |  |  |  |  |  |  |  |  |  |  | Critically Low |
| Zang et al. (2022) |  |  |  |  |  |  |  |  |  |  |  |  |  |  |  |  | Low |
| Rasking et al. (2022) |  |  |  |  |  |  |  |  |  |  |  |  |  |  |  |  | Low |
| Dimala et al. (2022) |  |  |  |  |  |  |  |  |  |  |  |  |  |  |  |  | Critically Low |
| Guo et al. (2022) |  |  |  |  |  |  |  |  |  |  |  |  |  |  |  |  | Low |
| Holm et al. (2021) |  |  |  |  |  |  |  |  |  |  |  |  |  |  |  |  | Critically Low |
| Lin et al. (2022) |  |  |  |  |  |  |  |  |  |  |  |  |  |  |  |  | Critically Low |
| Xu et al. (2022) |  |  |  |  |  |  |  |  |  |  |  |  |  |  |  |  | Critically Low |
| Yue et al. (2022) |  |  |  |  |  |  |  |  |  |  |  |  |  |  |  |  | Low |
| Manullang et al. (2022) |  |  |  |  |  |  |  |  |  |  |  |  |  |  |  |  | Critically Low |
| Gasana et al. (2012) |  |  |  |  |  |  |  |  |  |  |  |  |  |  |  |  | Critically Low |
| Liu et al. (2023) |  |  |  |  |  |  |  |  |  |  |  |  |  |  |  |  | Critically Low |
| Mason et al. (2022) |  |  |  |  |  |  |  |  |  |  |  |  |  |  |  |  | Critically Low |
| Arsad et al. (2022) |  |  |  |  |  |  |  |  |  |  |  |  |  |  |  |  | Critically Low |
| Faurie et al. (2022) |  |  |  |  |  |  |  |  |  |  |  |  |  |  |  |  | Low |
| Han et al. (2023) |  |  |  |  |  |  |  |  |  |  |  |  |  |  |  |  | Critically Low |
| Benmarhnia et al. (2015) |  |  |  |  |  |  |  |  |  |  |  |  |  |  |  |  | Critically Low |
| Cong et al, (2017) |  |  |  |  |  |  |  |  |  |  |  |  |  |  |  |  | Critically Low |
| Lakhoo et al. (2022) |  |  |  |  |  |  |  |  |  |  |  |  |  |  |  |  | Critically Low |
| Liu et al. (2021) |  |  |  |  |  |  |  |  |  |  |  |  |  |  |  |  | Low |
| Zheng et al. (2021) |  |  |  |  |  |  |  |  |  |  |  |  |  |  |  |  | Critically Low |
| Wang et al. (2021) |  |  |  |  |  |  |  |  |  |  |  |  |  |  |  |  | Critically Low |
| Wu et al. (2022) |  |  |  |  |  |  |  |  |  |  |  |  |  |  |  |  | Critically Low |
| Islam and Noor (2022) |  |  |  |  |  |  |  |  |  |  |  |  |  |  |  |  | Critically Low |
| Wang et al. (2022) |  |  |  |  |  |  |  |  |  |  |  |  |  |  |  |  | Critically Low |
| Manyuchi et al. (2022) |  |  |  |  |  |  |  |  |  |  |  |  |  |  |  |  | Critically Low |
| Lee et al. (2019) |  |  |  |  |  |  |  |  |  |  |  |  |  |  |  |  | Critically Low |
| Gao et al. (2022) |  |  |  |  |  |  |  |  |  |  |  |  |  |  |  |  | Critically Low |
| Krittanawong et al. (2023) |  |  |  |  |  |  |  |  |  |  |  |  |  |  |  |  | Critically Low |
| Pyo et al. (2022) |  |  |  |  |  |  |  |  |  |  |  |  |  |  |  |  | Critically Low |
| Hernandez Carballo et al, (2022) |  |  |  |  |  |  |  |  |  |  |  |  |  |  |  |  | Critically Low |
| Rezayat et al. (2022) |  |  |  |  |  |  |  |  |  |  |  |  |  |  |  |  | Critically Low |
| Gan et al. (2023) |  |  |  |  |  |  |  |  |  |  |  |  |  |  |  |  | Critically Low |
| Juneja Gandhi et al. (2022) |  |  |  |  |  |  |  |  |  |  |  |  |  |  |  |  | Critically Low |
| Badida et al. (2023) |  |  |  |  |  |  |  |  |  |  |  |  |  |  |  |  | Critically Low |
| Zhang et al. (2023) |  |  |  |  |  |  |  |  |  |  |  |  |  |  |  |  | Critically Low |
| Podury et al. (2023) |  |  |  |  |  |  |  |  |  |  |  |  |  |  |  |  | Low |
| Wang and Cao (2022) |  |  |  |  |  |  |  |  |  |  |  |  |  |  |  |  | Critically Low |
| Ju et al. (2023) |  |  |  |  |  |  |  |  |  |  |  |  |  |  |  |  | Critically Low |
| Luo et al. (2023) |  |  |  |  |  |  |  |  |  |  |  |  |  |  |  |  | Critically Low |
| Zhu et al. (2023) |  |  |  |  |  |  |  |  |  |  |  |  |  |  |  |  | Low |
| Yang et al. (2022) |  |  |  |  |  |  |  |  |  |  |  |  |  |  |  |  | Low |
| Yang et al. (2023) |  |  |  |  |  |  |  |  |  |  |  |  |  |  |  |  | Critically Low |
| Chung et al. (2022) |  |  |  |  |  |  |  |  |  |  |  |  |  |  |  |  | Critically Low |
| Husaini et al, (2022) |  |  |  |  |  |  |  |  |  |  |  |  |  |  |  |  | Critically Low |
| Ruan and Zeng (2023) |  |  |  |  |  |  |  |  |  |  |  |  |  |  |  |  | Critically Low |
| Tabaei et al, (2023) |  |  |  |  |  |  |  |  |  |  |  |  |  |  |  |  | Critically Low |
| Zhang et al. (2022) |  |  |  |  |  |  |  |  |  |  |  |  |  |  |  |  | Critically Low |
| Zhang et al. (2022) |  |  |  |  |  |  |  |  |  |  |  |  |  |  |  |  | Critically Low |
| Sui et al. (2022) |  |  |  |  |  |  |  |  |  |  |  |  |  |  |  |  | Critically Low |
| Ngoc et al. (2017) |  |  |  |  |  |  |  |  |  |  |  |  |  |  |  |  | Critically Low |
| Fu et al. (2019) |  |  |  |  |  |  |  |  |  |  |  |  |  |  |  |  | Low |
| Han et al. (2019) |  |  |  |  |  |  |  |  |  |  |  |  |  |  |  |  | Critically Low |
| Hu et al. (2019) |  |  |  |  |  |  |  |  |  |  |  |  |  |  |  |  | Critically Low |
| Ohlwein et al. (2019) |  |  |  |  |  |  |  |  |  |  |  |  |  |  |  |  | Critically Low |
| Oliveira et al. (2019) |  |  |  |  |  |  |  |  |  |  |  |  |  |  |  |  | Critically Low |
| Tsoli et al. (2019) |  |  |  |  |  |  |  |  |  |  |  |  |  |  |  |  | Critically Low |
| Fan et al. (2020) |  |  |  |  |  |  |  |  |  |  |  |  |  |  |  |  | Critically Low |
| Huang et al. (2020) |  |  |  |  |  |  |  |  |  |  |  |  |  |  |  |  | Critically Low |
| Samoli et al. (2020) |  |  |  |  |  |  |  |  |  |  |  |  |  |  |  |  | Critically Low |
| Yan et al. (2020) |  |  |  |  |  |  |  |  |  |  |  |  |  |  |  |  | Critically Low |
| Yu et al. (2020) |  |  |  |  |  |  |  |  |  |  |  |  |  |  |  |  | Critically Low |
| Huang et al. (2021) |  |  |  |  |  |  |  |  |  |  |  |  |  |  |  |  | Low |
| Huang et al. (2021) |  |  |  |  |  |  |  |  |  |  |  |  |  |  |  |  | Critically Low |
| Ju et al. (2021). |  |  |  |  |  |  |  |  |  |  |  |  |  |  |  |  | Critically Low |
| Lin et al. (2021) |  |  |  |  |  |  |  |  |  |  |  |  |  |  |  |  | Low |
| Liu et al. (2021) |  |  |  |  |  |  |  |  |  |  |  |  |  |  |  |  | Critically Low |
| McDermott-Levy et al. (2021) |  |  |  |  |  |  |  |  |  |  |  |  |  |  |  |  | Critically Low |
| Ni et al. (2021) |  |  |  |  |  |  |  |  |  |  |  |  |  |  |  |  | Critically Low |
| Zang et al. (2021) |  |  |  |  |  |  |  |  |  |  |  |  |  |  |  |  | Moderate |
| Borroni et al. (2022) |  |  |  |  |  |  |  |  |  |  |  |  |  |  |  |  | Critically Low |
| Cheng et al. (2022) |  |  |  |  |  |  |  |  |  |  |  |  |  |  |  |  | Low |
| Juarez et al. (2022) |  |  |  |  |  |  |  |  |  |  |  |  |  |  |  |  | Critically Low |
| Puthota et al. (2022) |  |  |  |  |  |  |  |  |  |  |  |  |  |  |  |  | Critically Low |
| Zhang et al. (2022) |  |  |  |  |  |  |  |  |  |  |  |  |  |  |  |  | Critically Low |
| Zong et al. (2022) |  |  |  |  |  |  |  |  |  |  |  |  |  |  |  |  | Critically Low |
| Blanc et al. (2023) |  |  |  |  |  |  |  |  |  |  |  |  |  |  |  |  | Low |
| Bouchriti et al. (2023 |  |  |  |  |  |  |  |  |  |  |  |  |  |  |  |  | Critically Low |
| Bougea et al. (2023) |  |  |  |  |  |  |  |  |  |  |  |  |  |  |  |  | Critically Low |
| Fadadu et al. (2023) |  |  |  |  |  |  |  |  |  |  |  |  |  |  |  |  | Low |
| Fan et al. (2023) |  |  |  |  |  |  |  |  |  |  |  |  |  |  |  |  | Critically Low |
| Guo et al. (2023) |  |  |  |  |  |  |  |  |  |  |  |  |  |  |  |  | Critically Low |
| Huang et al. (2023) |  |  |  |  |  |  |  |  |  |  |  |  |  |  |  |  | Low |
| Jia et al. (2023) |  |  |  |  |  |  |  |  |  |  |  |  |  |  |  |  | Critically Low |
| Karimi and Samadi (2023) |  |  |  |  |  |  |  |  |  |  |  |  |  |  |  |  | Low |
| Li et al. (2023) |  |  |  |  |  |  |  |  |  |  |  |  |  |  |  |  | Low |
| Liu et al. (2023) |  |  |  |  |  |  |  |  |  |  |  |  |  |  |  |  | Critically Low |
| Liu et al. (2023) |  |  |  |  |  |  |  |  |  |  |  |  |  |  |  |  | Low |
| Liu et al. (2023) |  |  |  |  |  |  |  |  |  |  |  |  |  |  |  |  | Low |
| Liu et al. (2023) |  |  |  |  |  |  |  |  |  |  |  |  |  |  |  |  | Critically Low |
| Luben et al. (2023) |  |  |  |  |  |  |  |  |  |  |  |  |  |  |  |  | Critically Low |
| Pan et al. (2023) |  |  |  |  |  |  |  |  |  |  |  |  |  |  |  |  | Critically Low |
| Parasin et al. (2023) |  |  |  |  |  |  |  |  |  |  |  |  |  |  |  |  | Low |
| Sheppard et al. (2023) |  |  |  |  |  |  |  |  |  |  |  |  |  |  |  |  | High |
| Tandon et al. (2023) |  |  |  |  |  |  |  |  |  |  |  |  |  |  |  |  | Critically Low |
| Thompson et al. (2023) |  |  |  |  |  |  |  |  |  |  |  |  |  |  |  |  | Low |
| Wan et al. (2023) |  |  |  |  |  |  |  |  |  |  |  |  |  |  |  |  | Low |
| Wang et al. (2023) |  |  |  |  |  |  |  |  |  |  |  |  |  |  |  |  | Critically Low |
| Wen et al. (2023) |  |  |  |  |  |  |  |  |  |  |  |  |  |  |  |  | Critically Low |
| Wu et al. (2023) |  |  |  |  |  |  |  |  |  |  |  |  |  |  |  |  | Critically Low |
| Yu et al. (2023) |  |  |  |  |  |  |  |  |  |  |  |  |  |  |  |  | Low |
| Yu et al. (2023) |  |  |  |  |  |  |  |  |  |  |  |  |  |  |  |  | Critically Low |
| Zhai et al. (2023) |  |  |  |  |  |  |  |  |  |  |  |  |  |  |  |  | Low |
| Zhang et al. (2023) |  |  |  |  |  |  |  |  |  |  |  |  |  |  |  |  | Critically Low |

**Table S5** The detailed results of the PRISMA checklist assessment of all the included studies

|  | Total Compliance |
| --- | --- |
|  | Partial Compliance |
|  | No Compliance |

|  | **1** | **2** | **3** | **4** | **5** | **6** | **7** | **8** | **9** | **10** | **11** | **12** | **13** | **14** | **15** | **16** | **17** | **18** | **19** | **20** | **21** | **22** | **23** | **24** | **25** | **26** | **27** | **Level** |
| --- | --- | --- | --- | --- | --- | --- | --- | --- | --- | --- | --- | --- | --- | --- | --- | --- | --- | --- | --- | --- | --- | --- | --- | --- | --- | --- | --- | --- |
| Requia et al. (2018) |  |  |  |  |  |  |  |  |  |  |  |  |  |  |  |  |  |  |  |  |  |  |  |  |  |  |  | Minor Flaws |
| Stieb et al. (2020) |  |  |  |  |  |  |  |  |  |  |  |  |  |  |  |  |  |  |  |  |  |  |  |  |  |  |  | Minimal Flaws |
| Ren et al. (2017) |  |  |  |  |  |  |  |  |  |  |  |  |  |  |  |  |  |  |  |  |  |  |  |  |  |  |  | Minimal Flaws |
| Cui et al. (2015) |  |  |  |  |  |  |  |  |  |  |  |  |  |  |  |  |  |  |  |  |  |  |  |  |  |  |  | Minimal Flaws |
| Glinianaia et al. (2004a) |  |  |  |  |  |  |  |  |  |  |  |  |  |  |  |  |  |  |  |  |  |  |  |  |  |  |  | Major Flaws |
| Kotsyfakis et al. (2019) |  |  |  |  |  |  |  |  |  |  |  |  |  |  |  |  |  |  |  |  |  |  |  |  |  |  |  | Major Flaws |
| Wong et al. (2004) |  |  |  |  |  |  |  |  |  |  |  |  |  |  |  |  |  |  |  |  |  |  |  |  |  |  |  | Major Flaws |
| Nhung et al. (2017) |  |  |  |  |  |  |  |  |  |  |  |  |  |  |  |  |  |  |  |  |  |  |  |  |  |  |  | Minimal Flaws |
| Vrijheid et al. (2011) |  |  |  |  |  |  |  |  |  |  |  |  |  |  |  |  |  |  |  |  |  |  |  |  |  |  |  | Minor Flaws |
| Pedersen et al. (2014) |  |  |  |  |  |  |  |  |  |  |  |  |  |  |  |  |  |  |  |  |  |  |  |  |  |  |  | Minor Flaws |
| Atkinson et al. (2015) |  |  |  |  |  |  |  |  |  |  |  |  |  |  |  |  |  |  |  |  |  |  |  |  |  |  |  | Minor Flaws |
| Zhang et al. (2016a) |  |  |  |  |  |  |  |  |  |  |  |  |  |  |  |  |  |  |  |  |  |  |  |  |  |  |  | Minimal Flaws |
| Ravindra et al. (2020) |  |  |  |  |  |  |  |  |  |  |  |  |  |  |  |  |  |  |  |  |  |  |  |  |  |  |  | Minimal Flaws |
| Newell et al. (2017) |  |  |  |  |  |  |  |  |  |  |  |  |  |  |  |  |  |  |  |  |  |  |  |  |  |  |  | Minimal Flaws |
| Braithwaite et al. (2019) |  |  |  |  |  |  |  |  |  |  |  |  |  |  |  |  |  |  |  |  |  |  |  |  |  |  |  | Minimal Flaws |
| Orellano et al. (2017) |  |  |  |  |  |  |  |  |  |  |  |  |  |  |  |  |  |  |  |  |  |  |  |  |  |  |  | Minimal Flaws |
| Chen et al. (2014) |  |  |  |  |  |  |  |  |  |  |  |  |  |  |  |  |  |  |  |  |  |  |  |  |  |  |  | Minor Flaws |
| Song et al. (2014) |  |  |  |  |  |  |  |  |  |  |  |  |  |  |  |  |  |  |  |  |  |  |  |  |  |  |  | Minor Flaws |
| Yang et al. (2020) |  |  |  |  |  |  |  |  |  |  |  |  |  |  |  |  |  |  |  |  |  |  |  |  |  |  |  | Minimal Flaws |
| Song et al. (2016) |  |  |  |  |  |  |  |  |  |  |  |  |  |  |  |  |  |  |  |  |  |  |  |  |  |  |  | Minimal Flaws |
| Shah et al. (2013) |  |  |  |  |  |  |  |  |  |  |  |  |  |  |  |  |  |  |  |  |  |  |  |  |  |  |  | Minor Flaws |
| Newell et al. (2018) |  |  |  |  |  |  |  |  |  |  |  |  |  |  |  |  |  |  |  |  |  |  |  |  |  |  |  | Minimal Flaws |
| Kim et al. (2018) |  |  |  |  |  |  |  |  |  |  |  |  |  |  |  |  |  |  |  |  |  |  |  |  |  |  |  | Minimal Flaws |
| Kihal et al. (2020) |  |  |  |  |  |  |  |  |  |  |  |  |  |  |  |  |  |  |  |  |  |  |  |  |  |  |  | Minimal Flaws |
| Zhao et al. (2017) |  |  |  |  |  |  |  |  |  |  |  |  |  |  |  |  |  |  |  |  |  |  |  |  |  |  |  | Minimal Flaws |
| Scheers et al. (2015) |  |  |  |  |  |  |  |  |  |  |  |  |  |  |  |  |  |  |  |  |  |  |  |  |  |  |  | Minimal Flaws |
| Siddika et al. (2016) |  |  |  |  |  |  |  |  |  |  |  |  |  |  |  |  |  |  |  |  |  |  |  |  |  |  |  | Minimal Flaws |
| Liu et al. (2020) |  |  |  |  |  |  |  |  |  |  |  |  |  |  |  |  |  |  |  |  |  |  |  |  |  |  |  | Minimal Flaws |
| Shah et al. (2015) |  |  |  |  |  |  |  |  |  |  |  |  |  |  |  |  |  |  |  |  |  |  |  |  |  |  |  | Minimal Flaws |
| Chang et al. (2015) |  |  |  |  |  |  |  |  |  |  |  |  |  |  |  |  |  |  |  |  |  |  |  |  |  |  |  | Minor Flaws |
| Shang et al. (2013) |  |  |  |  |  |  |  |  |  |  |  |  |  |  |  |  |  |  |  |  |  |  |  |  |  |  |  | Major Flaws |
| Li et al. (2014) |  |  |  |  |  |  |  |  |  |  |  |  |  |  |  |  |  |  |  |  |  |  |  |  |  |  |  | Minimal Flaws |
| Yang et al. (2016) |  |  |  |  |  |  |  |  |  |  |  |  |  |  |  |  |  |  |  |  |  |  |  |  |  |  |  | Minimal Flaws |
| Bekkar et al. (2020) |  |  |  |  |  |  |  |  |  |  |  |  |  |  |  |  |  |  |  |  |  |  |  |  |  |  |  | Major Flaws |
| Yang et al. (2014) |  |  |  |  |  |  |  |  |  |  |  |  |  |  |  |  |  |  |  |  |  |  |  |  |  |  |  | Minor Flaws |
| Lai et al. (2013) |  |  |  |  |  |  |  |  |  |  |  |  |  |  |  |  |  |  |  |  |  |  |  |  |  |  |  | Minor Flaws |
| Huangfu et al. (2020) |  |  |  |  |  |  |  |  |  |  |  |  |  |  |  |  |  |  |  |  |  |  |  |  |  |  |  | Minimal Flaws |
| DeVries et al. (2017) |  |  |  |  |  |  |  |  |  |  |  |  |  |  |  |  |  |  |  |  |  |  |  |  |  |  |  | Minor Flaws |
| Vodonos et al. (2018) |  |  |  |  |  |  |  |  |  |  |  |  |  |  |  |  |  |  |  |  |  |  |  |  |  |  |  | Minor Flaws |
| Yu et al. (2014) |  |  |  |  |  |  |  |  |  |  |  |  |  |  |  |  |  |  |  |  |  |  |  |  |  |  |  | Minimal Flaws |
| Gao et al. (2020) |  |  |  |  |  |  |  |  |  |  |  |  |  |  |  |  |  |  |  |  |  |  |  |  |  |  |  | Minor Flaws |
| Wang et al. (2019) |  |  |  |  |  |  |  |  |  |  |  |  |  |  |  |  |  |  |  |  |  |  |  |  |  |  |  | Minor Flaws |
| Wang et al. (2014a) |  |  |  |  |  |  |  |  |  |  |  |  |  |  |  |  |  |  |  |  |  |  |  |  |  |  |  | Minor Flaws |
| Chen et al. (2020) |  |  |  |  |  |  |  |  |  |  |  |  |  |  |  |  |  |  |  |  |  |  |  |  |  |  |  | Minimal Flaws |
| Mills et al. (2016) |  |  |  |  |  |  |  |  |  |  |  |  |  |  |  |  |  |  |  |  |  |  |  |  |  |  |  | Minor Flaws |
| Zhu et al. (2013) |  |  |  |  |  |  |  |  |  |  |  |  |  |  |  |  |  |  |  |  |  |  |  |  |  |  |  | Minor Flaws |
| Bell et al. (2013) |  |  |  |  |  |  |  |  |  |  |  |  |  |  |  |  |  |  |  |  |  |  |  |  |  |  |  | Minor Flaws |
| Sakhvidi et al. (2020) |  |  |  |  |  |  |  |  |  |  |  |  |  |  |  |  |  |  |  |  |  |  |  |  |  |  |  | Minor Flaws |
| Achilleos et al. (2017) |  |  |  |  |  |  |  |  |  |  |  |  |  |  |  |  |  |  |  |  |  |  |  |  |  |  |  | Minimal Flaws |
| Hoek et al. (2013) |  |  |  |  |  |  |  |  |  |  |  |  |  |  |  |  |  |  |  |  |  |  |  |  |  |  |  | Major Flaws |
| Lee et al. (2020) |  |  |  |  |  |  |  |  |  |  |  |  |  |  |  |  |  |  |  |  |  |  |  |  |  |  |  | Minimal Flaws |
| Zhu et al. (2020) |  |  |  |  |  |  |  |  |  |  |  |  |  |  |  |  |  |  |  |  |  |  |  |  |  |  |  | Minimal Flaws |
| Pranata et al. (2020) |  |  |  |  |  |  |  |  |  |  |  |  |  |  |  |  |  |  |  |  |  |  |  |  |  |  |  | Minor Flaws |
| Hamra et al. (2014) |  |  |  |  |  |  |  |  |  |  |  |  |  |  |  |  |  |  |  |  |  |  |  |  |  |  |  | Minor Flaws |
| Bell et al. (2014) |  |  |  |  |  |  |  |  |  |  |  |  |  |  |  |  |  |  |  |  |  |  |  |  |  |  |  | Minor Flaws |
| Zhao et al. (2018) |  |  |  |  |  |  |  |  |  |  |  |  |  |  |  |  |  |  |  |  |  |  |  |  |  |  |  | Minor Flaws |
| Fu et al. (2019) |  |  |  |  |  |  |  |  |  |  |  |  |  |  |  |  |  |  |  |  |  |  |  |  |  |  |  | Minimal Flaws |
| Yan et al. (2013) |  |  |  |  |  |  |  |  |  |  |  |  |  |  |  |  |  |  |  |  |  |  |  |  |  |  |  | Minor Flaws |
| Zheng et al. (2015) |  |  |  |  |  |  |  |  |  |  |  |  |  |  |  |  |  |  |  |  |  |  |  |  |  |  |  | Minimal Flaws |
| Zhang et al. (2018) |  |  |  |  |  |  |  |  |  |  |  |  |  |  |  |  |  |  |  |  |  |  |  |  |  |  |  | Minor Flaws |
| Ito et al. (2005) |  |  |  |  |  |  |  |  |  |  |  |  |  |  |  |  |  |  |  |  |  |  |  |  |  |  |  | Major Flaws |
| Cai et al. (2016) |  |  |  |  |  |  |  |  |  |  |  |  |  |  |  |  |  |  |  |  |  |  |  |  |  |  |  | Minor Flaws |
| Sun et al. (2017) |  |  |  |  |  |  |  |  |  |  |  |  |  |  |  |  |  |  |  |  |  |  |  |  |  |  |  | Minimal Flaws |
| Park et al. (2013) |  |  |  |  |  |  |  |  |  |  |  |  |  |  |  |  |  |  |  |  |  |  |  |  |  |  |  | Minor Flaws |
| Li et al. (2016a) |  |  |  |  |  |  |  |  |  |  |  |  |  |  |  |  |  |  |  |  |  |  |  |  |  |  |  | Minor Flaws |
| Yuan et al. (2019) |  |  |  |  |  |  |  |  |  |  |  |  |  |  |  |  |  |  |  |  |  |  |  |  |  |  |  | Minimal Flaws |
| Mills et al. (2015) |  |  |  |  |  |  |  |  |  |  |  |  |  |  |  |  |  |  |  |  |  |  |  |  |  |  |  | Minor Flaws |
| Huang et al. (2017) |  |  |  |  |  |  |  |  |  |  |  |  |  |  |  |  |  |  |  |  |  |  |  |  |  |  |  | Minimal Flaws |
| Lu et al. (2015) |  |  |  |  |  |  |  |  |  |  |  |  |  |  |  |  |  |  |  |  |  |  |  |  |  |  |  | Minor Flaws |
| Orellano et al. (2020) |  |  |  |  |  |  |  |  |  |  |  |  |  |  |  |  |  |  |  |  |  |  |  |  |  |  |  | Minimal Flaws |
| Atkinson et al. (2014) |  |  |  |  |  |  |  |  |  |  |  |  |  |  |  |  |  |  |  |  |  |  |  |  |  |  |  | Minor Flaws |
| Yang et al. (2019a) |  |  |  |  |  |  |  |  |  |  |  |  |  |  |  |  |  |  |  |  |  |  |  |  |  |  |  | Minimal Flaws |
| Liu et al. (2018) |  |  |  |  |  |  |  |  |  |  |  |  |  |  |  |  |  |  |  |  |  |  |  |  |  |  |  | Minimal Flaws |
| Atkinson et al. (2018) |  |  |  |  |  |  |  |  |  |  |  |  |  |  |  |  |  |  |  |  |  |  |  |  |  |  |  | Minor Flaws |
| Glinianaia et al. (2004b) |  |  |  |  |  |  |  |  |  |  |  |  |  |  |  |  |  |  |  |  |  |  |  |  |  |  |  | Major Flaws |
| Atkinson et al. (2016) |  |  |  |  |  |  |  |  |  |  |  |  |  |  |  |  |  |  |  |  |  |  |  |  |  |  |  | Minor Flaws |
| Faustini et al. (2014) |  |  |  |  |  |  |  |  |  |  |  |  |  |  |  |  |  |  |  |  |  |  |  |  |  |  |  | Minor Flaws |
| Stieb et al. (2002) |  |  |  |  |  |  |  |  |  |  |  |  |  |  |  |  |  |  |  |  |  |  |  |  |  |  |  | Major Flaws |
| Zhang et al. (2019) |  |  |  |  |  |  |  |  |  |  |  |  |  |  |  |  |  |  |  |  |  |  |  |  |  |  |  | Minimal Flaws |
| Ab Manan et al. (2018) |  |  |  |  |  |  |  |  |  |  |  |  |  |  |  |  |  |  |  |  |  |  |  |  |  |  |  | Major Flaws |
| Guo et al. (2020) |  |  |  |  |  |  |  |  |  |  |  |  |  |  |  |  |  |  |  |  |  |  |  |  |  |  |  | Minimal Flaws |
| Luben et al. (2017) |  |  |  |  |  |  |  |  |  |  |  |  |  |  |  |  |  |  |  |  |  |  |  |  |  |  |  | Minor Flaws |
| Koranteng et al. (2007) |  |  |  |  |  |  |  |  |  |  |  |  |  |  |  |  |  |  |  |  |  |  |  |  |  |  |  | Major Flaws |
| Yang et al. (2019b) |  |  |  |  |  |  |  |  |  |  |  |  |  |  |  |  |  |  |  |  |  |  |  |  |  |  |  | Minor Flaws |
| Jacobs et al. (2017) |  |  |  |  |  |  |  |  |  |  |  |  |  |  |  |  |  |  |  |  |  |  |  |  |  |  |  | Major Flaws |
| Jaganathan et al. (2019) |  |  |  |  |  |  |  |  |  |  |  |  |  |  |  |  |  |  |  |  |  |  |  |  |  |  |  | Minor Flaws |
| Wu et al. (2018) |  |  |  |  |  |  |  |  |  |  |  |  |  |  |  |  |  |  |  |  |  |  |  |  |  |  |  | Minor Flaws |
| Moore et al. (2016) |  |  |  |  |  |  |  |  |  |  |  |  |  |  |  |  |  |  |  |  |  |  |  |  |  |  |  | Minimal Flaws |
| Goldberg et al. (2008) |  |  |  |  |  |  |  |  |  |  |  |  |  |  |  |  |  |  |  |  |  |  |  |  |  |  |  | Minor Flaws |
| King et al. (2018) |  |  |  |  |  |  |  |  |  |  |  |  |  |  |  |  |  |  |  |  |  |  |  |  |  |  |  | Minor Flaws |
| Bazyar et al. (2019) |  |  |  |  |  |  |  |  |  |  |  |  |  |  |  |  |  |  |  |  |  |  |  |  |  |  |  | Major Flaws |
| Yamamoto et al. (2014) |  |  |  |  |  |  |  |  |  |  |  |  |  |  |  |  |  |  |  |  |  |  |  |  |  |  |  | Major Flaws |
| Levy et al. (2005) |  |  |  |  |  |  |  |  |  |  |  |  |  |  |  |  |  |  |  |  |  |  |  |  |  |  |  | Major Flaws |
| Copat et al. (2020) |  |  |  |  |  |  |  |  |  |  |  |  |  |  |  |  |  |  |  |  |  |  |  |  |  |  |  | Major Flaws |
| Rajak et al. (2020) |  |  |  |  |  |  |  |  |  |  |  |  |  |  |  |  |  |  |  |  |  |  |  |  |  |  |  | Minor Flaws |
| Teng et al. (2014) |  |  |  |  |  |  |  |  |  |  |  |  |  |  |  |  |  |  |  |  |  |  |  |  |  |  |  | Minor Flaws |
| Jilani et al. (2020) |  |  |  |  |  |  |  |  |  |  |  |  |  |  |  |  |  |  |  |  |  |  |  |  |  |  |  | Major Flaws |
| Volk et al. (2020) |  |  |  |  |  |  |  |  |  |  |  |  |  |  |  |  |  |  |  |  |  |  |  |  |  |  |  | Major Flaws |
| Abdo et al. (2016) |  |  |  |  |  |  |  |  |  |  |  |  |  |  |  |  |  |  |  |  |  |  |  |  |  |  |  | Major Flaws |
| Popovic et al. (2019) |  |  |  |  |  |  |  |  |  |  |  |  |  |  |  |  |  |  |  |  |  |  |  |  |  |  |  | Minor Flaws |
| Buoli et al. (2018) |  |  |  |  |  |  |  |  |  |  |  |  |  |  |  |  |  |  |  |  |  |  |  |  |  |  |  | Major Flaws |
| Li et al. (2019) |  |  |  |  |  |  |  |  |  |  |  |  |  |  |  |  |  |  |  |  |  |  |  |  |  |  |  | Minimal Flaws |
| Asmus et al. (2016) |  |  |  |  |  |  |  |  |  |  |  |  |  |  |  |  |  |  |  |  |  |  |  |  |  |  |  | Major Flaws |
| Liu et al. (2016) |  |  |  |  |  |  |  |  |  |  |  |  |  |  |  |  |  |  |  |  |  |  |  |  |  |  |  | Major Flaws |
| Latza et al. (2009) |  |  |  |  |  |  |  |  |  |  |  |  |  |  |  |  |  |  |  |  |  |  |  |  |  |  |  | Minor Flaws |
| Porpora et al. (2019) |  |  |  |  |  |  |  |  |  |  |  |  |  |  |  |  |  |  |  |  |  |  |  |  |  |  |  | Major Flaws |
| Wyzga et al. (2015) |  |  |  |  |  |  |  |  |  |  |  |  |  |  |  |  |  |  |  |  |  |  |  |  |  |  |  | Major Flaws |
| Wang et al. (2018) |  |  |  |  |  |  |  |  |  |  |  |  |  |  |  |  |  |  |  |  |  |  |  |  |  |  |  | Minor Flaws |
| Nasser et al. (2015) |  |  |  |  |  |  |  |  |  |  |  |  |  |  |  |  |  |  |  |  |  |  |  |  |  |  |  | Major Flaws |
| Luben et al. (2018) |  |  |  |  |  |  |  |  |  |  |  |  |  |  |  |  |  |  |  |  |  |  |  |  |  |  |  | Major Flaws |
| Pelucchi et al. (2009) |  |  |  |  |  |  |  |  |  |  |  |  |  |  |  |  |  |  |  |  |  |  |  |  |  |  |  | Major Flaws |
| Maitre et al. (2006) |  |  |  |  |  |  |  |  |  |  |  |  |  |  |  |  |  |  |  |  |  |  |  |  |  |  |  | Major Flaws |
| Mustafić et al. (2012) |  |  |  |  |  |  |  |  |  |  |  |  |  |  |  |  |  |  |  |  |  |  |  |  |  |  |  | Minimal Flaws |
| Mehta et al. (2013) |  |  |  |  |  |  |  |  |  |  |  |  |  |  |  |  |  |  |  |  |  |  |  |  |  |  |  | Minor Flaws |
| Wang et al. (2014b) |  |  |  |  |  |  |  |  |  |  |  |  |  |  |  |  |  |  |  |  |  |  |  |  |  |  |  | Minimal Flaws |
| Ji et al. (2011) |  |  |  |  |  |  |  |  |  |  |  |  |  |  |  |  |  |  |  |  |  |  |  |  |  |  |  | Minor Flaws |
| Li et al. (2016b) |  |  |  |  |  |  |  |  |  |  |  |  |  |  |  |  |  |  |  |  |  |  |  |  |  |  |  | Minimal Flaws |
| Morris et al. (2001) |  |  |  |  |  |  |  |  |  |  |  |  |  |  |  |  |  |  |  |  |  |  |  |  |  |  |  | Major Flaws |
| Janghorbani et al. (2014) |  |  |  |  |  |  |  |  |  |  |  |  |  |  |  |  |  |  |  |  |  |  |  |  |  |  |  | Minor Flaws |
| Atkinson et al. (2012) |  |  |  |  |  |  |  |  |  |  |  |  |  |  |  |  |  |  |  |  |  |  |  |  |  |  |  | Minor Flaws |
| Zhang et al. (2016b) |  |  |  |  |  |  |  |  |  |  |  |  |  |  |  |  |  |  |  |  |  |  |  |  |  |  |  | Major Flaws |
| Luo et al. (2015) |  |  |  |  |  |  |  |  |  |  |  |  |  |  |  |  |  |  |  |  |  |  |  |  |  |  |  | Minimal Flaws |
| Peters et al. (2019) |  |  |  |  |  |  |  |  |  |  |  |  |  |  |  |  |  |  |  |  |  |  |  |  |  |  |  | Minor Flaws |
| Franchini et al. (2016) |  |  |  |  |  |  |  |  |  |  |  |  |  |  |  |  |  |  |  |  |  |  |  |  |  |  |  | Major Flaws |
| Akintoye et al. (2016) |  |  |  |  |  |  |  |  |  |  |  |  |  |  |  |  |  |  |  |  |  |  |  |  |  |  |  | Minor Flaws |
| Li et al. (2012) |  |  |  |  |  |  |  |  |  |  |  |  |  |  |  |  |  |  |  |  |  |  |  |  |  |  |  | Minor Flaws |
| Tsai et al. (2019) |  |  |  |  |  |  |  |  |  |  |  |  |  |  |  |  |  |  |  |  |  |  |  |  |  |  |  | Minimal Flaws |
| Spirić et al. (2012) |  |  |  |  |  |  |  |  |  |  |  |  |  |  |  |  |  |  |  |  |  |  |  |  |  |  |  | Major Flaws |
| Luong et al. (2019) |  |  |  |  |  |  |  |  |  |  |  |  |  |  |  |  |  |  |  |  |  |  |  |  |  |  |  | Minimal Flaws |
| Kim et al. (2020) |  |  |  |  |  |  |  |  |  |  |  |  |  |  |  |  |  |  |  |  |  |  |  |  |  |  |  | Minimal Flaws |
| Sun et al. (2020) |  |  |  |  |  |  |  |  |  |  |  |  |  |  |  |  |  |  |  |  |  |  |  |  |  |  |  | Minimal Flaws |
| Chen et al. (2019) |  |  |  |  |  |  |  |  |  |  |  |  |  |  |  |  |  |  |  |  |  |  |  |  |  |  |  | Minimal Flaws |
| Sharma et al. (2020) |  |  |  |  |  |  |  |  |  |  |  |  |  |  |  |  |  |  |  |  |  |  |  |  |  |  |  | Major Flaws |
| Vieira et al. (2015) |  |  |  |  |  |  |  |  |  |  |  |  |  |  |  |  |  |  |  |  |  |  |  |  |  |  |  | Major Flaws |
| Bell et al. (2005) |  |  |  |  |  |  |  |  |  |  |  |  |  |  |  |  |  |  |  |  |  |  |  |  |  |  |  | Major Flaws |
| Kan et al. (2005) |  |  |  |  |  |  |  |  |  |  |  |  |  |  |  |  |  |  |  |  |  |  |  |  |  |  |  | Major Flaws |
| Conforti et al. (2018) |  |  |  |  |  |  |  |  |  |  |  |  |  |  |  |  |  |  |  |  |  |  |  |  |  |  |  | Major Flaws |
| Keramatinia et al. (2016) |  |  |  |  |  |  |  |  |  |  |  |  |  |  |  |  |  |  |  |  |  |  |  |  |  |  |  | Minor Flaws |
| Cheng et al. (2019) |  |  |  |  |  |  |  |  |  |  |  |  |  |  |  |  |  |  |  |  |  |  |  |  |  |  |  | Minimal Flaws |
| Green et al. (2019) |  |  |  |  |  |  |  |  |  |  |  |  |  |  |  |  |  |  |  |  |  |  |  |  |  |  |  | Major Flaws |
| van Steen et al. (2019) |  |  |  |  |  |  |  |  |  |  |  |  |  |  |  |  |  |  |  |  |  |  |  |  |  |  |  | Major Flaws |
| Sun et al. (2018) |  |  |  |  |  |  |  |  |  |  |  |  |  |  |  |  |  |  |  |  |  |  |  |  |  |  |  | Minimal Flaws |
| Campbell et al. (2018) |  |  |  |  |  |  |  |  |  |  |  |  |  |  |  |  |  |  |  |  |  |  |  |  |  |  |  | Major Flaws |
| Odame et al. (2018) |  |  |  |  |  |  |  |  |  |  |  |  |  |  |  |  |  |  |  |  |  |  |  |  |  |  |  | Minor Flaws |
| Ghanizadeh et al. (2017) |  |  |  |  |  |  |  |  |  |  |  |  |  |  |  |  |  |  |  |  |  |  |  |  |  |  |  | Major Flaws |
| Kuehn, L. and S. McCormick (2017) |  |  |  |  |  |  |  |  |  |  |  |  |  |  |  |  |  |  |  |  |  |  |  |  |  |  |  | Major Flaws |
| Zhang et al. (2017) |  |  |  |  |  |  |  |  |  |  |  |  |  |  |  |  |  |  |  |  |  |  |  |  |  |  |  | Major Flaws |
| Philipsborn et al. (2016) |  |  |  |  |  |  |  |  |  |  |  |  |  |  |  |  |  |  |  |  |  |  |  |  |  |  |  | Major Flaws |
| Asadgol et al. (2020) |  |  |  |  |  |  |  |  |  |  |  |  |  |  |  |  |  |  |  |  |  |  |  |  |  |  |  | Major Flaws |
| Bai et al. (2019) |  |  |  |  |  |  |  |  |  |  |  |  |  |  |  |  |  |  |  |  |  |  |  |  |  |  |  | Minor Flaws |
| Lu et al. (2018) |  |  |  |  |  |  |  |  |  |  |  |  |  |  |  |  |  |  |  |  |  |  |  |  |  |  |  | Minor Flaws |
| Coates et al. (2019) |  |  |  |  |  |  |  |  |  |  |  |  |  |  |  |  |  |  |  |  |  |  |  |  |  |  |  | Major Flaws |
| Shi et al. (2019) |  |  |  |  |  |  |  |  |  |  |  |  |  |  |  |  |  |  |  |  |  |  |  |  |  |  |  | Minimal Flaws |
| Son et al. (2019) |  |  |  |  |  |  |  |  |  |  |  |  |  |  |  |  |  |  |  |  |  |  |  |  |  |  |  | Major Flaws |
| Gao et al. (2019) |  |  |  |  |  |  |  |  |  |  |  |  |  |  |  |  |  |  |  |  |  |  |  |  |  |  |  | Minimal Flaws |
| Salve et al. (2018) |  |  |  |  |  |  |  |  |  |  |  |  |  |  |  |  |  |  |  |  |  |  |  |  |  |  |  | Major Flaws |
| Chersich et al. (2018) |  |  |  |  |  |  |  |  |  |  |  |  |  |  |  |  |  |  |  |  |  |  |  |  |  |  |  | Major Flaws |
| Cheng et al. (2018) |  |  |  |  |  |  |  |  |  |  |  |  |  |  |  |  |  |  |  |  |  |  |  |  |  |  |  | Minimal Flaws |
| Ghazani et al. (2018) |  |  |  |  |  |  |  |  |  |  |  |  |  |  |  |  |  |  |  |  |  |  |  |  |  |  |  | Major Flaws |
| Chan et al. (2019) |  |  |  |  |  |  |  |  |  |  |  |  |  |  |  |  |  |  |  |  |  |  |  |  |  |  |  | Major Flaws |
| Zanobetti and O'Neill (2018) |  |  |  |  |  |  |  |  |  |  |  |  |  |  |  |  |  |  |  |  |  |  |  |  |  |  |  | Major Flaws |
| Ma et al. (2020) |  |  |  |  |  |  |  |  |  |  |  |  |  |  |  |  |  |  |  |  |  |  |  |  |  |  |  | Major Flaws |
| Chersich et al. (2020) |  |  |  |  |  |  |  |  |  |  |  |  |  |  |  |  |  |  |  |  |  |  |  |  |  |  |  | Minor Flaws |
| Chang et al. (2020) |  |  |  |  |  |  |  |  |  |  |  |  |  |  |  |  |  |  |  |  |  |  |  |  |  |  |  | Major Flaws |
| Cheng et al. (2019) |  |  |  |  |  |  |  |  |  |  |  |  |  |  |  |  |  |  |  |  |  |  |  |  |  |  |  | Minor Flaws |
| Luo et al. (2019) |  |  |  |  |  |  |  |  |  |  |  |  |  |  |  |  |  |  |  |  |  |  |  |  |  |  |  | Minor Flaws |
| Bodaghkhani et al. (2019) |  |  |  |  |  |  |  |  |  |  |  |  |  |  |  |  |  |  |  |  |  |  |  |  |  |  |  | Major Flaws |
| Thompson et al. (2018) |  |  |  |  |  |  |  |  |  |  |  |  |  |  |  |  |  |  |  |  |  |  |  |  |  |  |  | Minor Flaws |
| Schinasi et al. (2018) |  |  |  |  |  |  |  |  |  |  |  |  |  |  |  |  |  |  |  |  |  |  |  |  |  |  |  | Minimal Flaws |
| Leyva et al. (2017) |  |  |  |  |  |  |  |  |  |  |  |  |  |  |  |  |  |  |  |  |  |  |  |  |  |  |  | Major Flaws |
| Geraghty et al. (2017) |  |  |  |  |  |  |  |  |  |  |  |  |  |  |  |  |  |  |  |  |  |  |  |  |  |  |  | Major Flaws |
| Moghadamnia et al. (2017) |  |  |  |  |  |  |  |  |  |  |  |  |  |  |  |  |  |  |  |  |  |  |  |  |  |  |  | Minimal Flaws |
| Mousavi et al. (2020) |  |  |  |  |  |  |  |  |  |  |  |  |  |  |  |  |  |  |  |  |  |  |  |  |  |  |  | Major Flaws |
| Arbuthnott et al. (2016) |  |  |  |  |  |  |  |  |  |  |  |  |  |  |  |  |  |  |  |  |  |  |  |  |  |  |  | Major Flaws |
| Heidari et al. (2020) |  |  |  |  |  |  |  |  |  |  |  |  |  |  |  |  |  |  |  |  |  |  |  |  |  |  |  | Major Flaws |
| Ainita et al. (2018) |  |  |  |  |  |  |  |  |  |  |  |  |  |  |  |  |  |  |  |  |  |  |  |  |  |  |  | Major Flaws |
| Otte im Kampe et al. (2016) |  |  |  |  |  |  |  |  |  |  |  |  |  |  |  |  |  |  |  |  |  |  |  |  |  |  |  | Major Flaws |
| Amegah et al. (2016) |  |  |  |  |  |  |  |  |  |  |  |  |  |  |  |  |  |  |  |  |  |  |  |  |  |  |  | Major Flaws |
| Astrom et al. (2011) |  |  |  |  |  |  |  |  |  |  |  |  |  |  |  |  |  |  |  |  |  |  |  |  |  |  |  | Major Flaws |
| Basu (2009 |  |  |  |  |  |  |  |  |  |  |  |  |  |  |  |  |  |  |  |  |  |  |  |  |  |  |  | Major Flaws |
| Martiello and Giacchi (2010) |  |  |  |  |  |  |  |  |  |  |  |  |  |  |  |  |  |  |  |  |  |  |  |  |  |  |  | Major Flaws |
| Witt et al. (2015) |  |  |  |  |  |  |  |  |  |  |  |  |  |  |  |  |  |  |  |  |  |  |  |  |  |  |  | Major Flaws |
| Turner et al. (2012) |  |  |  |  |  |  |  |  |  |  |  |  |  |  |  |  |  |  |  |  |  |  |  |  |  |  |  | Minor Flaws |
| Ye et al. (2012) |  |  |  |  |  |  |  |  |  |  |  |  |  |  |  |  |  |  |  |  |  |  |  |  |  |  |  | Major Flaws |
| Ryti et al. (2016) |  |  |  |  |  |  |  |  |  |  |  |  |  |  |  |  |  |  |  |  |  |  |  |  |  |  |  | Minor Flaws |
| Basu and Samet (2002) |  |  |  |  |  |  |  |  |  |  |  |  |  |  |  |  |  |  |  |  |  |  |  |  |  |  |  | Major Flaws |
| Bhaskaran et al. (2009) |  |  |  |  |  |  |  |  |  |  |  |  |  |  |  |  |  |  |  |  |  |  |  |  |  |  |  | Major Flaws |
| Carlton et al. (2016) |  |  |  |  |  |  |  |  |  |  |  |  |  |  |  |  |  |  |  |  |  |  |  |  |  |  |  | Minimal Flaws |
| Beltran et al. (2013) |  |  |  |  |  |  |  |  |  |  |  |  |  |  |  |  |  |  |  |  |  |  |  |  |  |  |  | Minor Flaws |
| Fan et al. (2014) |  |  |  |  |  |  |  |  |  |  |  |  |  |  |  |  |  |  |  |  |  |  |  |  |  |  |  | Minimal Flaws |
| Li et al. (2015) |  |  |  |  |  |  |  |  |  |  |  |  |  |  |  |  |  |  |  |  |  |  |  |  |  |  |  | Major Flaws |
| Lian et al. (2015) |  |  |  |  |  |  |  |  |  |  |  |  |  |  |  |  |  |  |  |  |  |  |  |  |  |  |  | Minimal Flaws |
| Bunker et al. (2016) |  |  |  |  |  |  |  |  |  |  |  |  |  |  |  |  |  |  |  |  |  |  |  |  |  |  |  | Minor Flaws |
| Xu et al. (2016) |  |  |  |  |  |  |  |  |  |  |  |  |  |  |  |  |  |  |  |  |  |  |  |  |  |  |  | Minor Flaws |
| Strand et al. (2011) |  |  |  |  |  |  |  |  |  |  |  |  |  |  |  |  |  |  |  |  |  |  |  |  |  |  |  | Major Flaws |
| Xu et al. (2012) |  |  |  |  |  |  |  |  |  |  |  |  |  |  |  |  |  |  |  |  |  |  |  |  |  |  |  | Major Flaws |
| Carolan-Olah and Frankowska (2014) |  |  |  |  |  |  |  |  |  |  |  |  |  |  |  |  |  |  |  |  |  |  |  |  |  |  |  | Major Flaws |
| Phung et al. (2016). |  |  |  |  |  |  |  |  |  |  |  |  |  |  |  |  |  |  |  |  |  |  |  |  |  |  |  | Minimal Flaws |
| Ramesh et al. (2013) |  |  |  |  |  |  |  |  |  |  |  |  |  |  |  |  |  |  |  |  |  |  |  |  |  |  |  | Major Flaws |
| Yu et al. (2012) |  |  |  |  |  |  |  |  |  |  |  |  |  |  |  |  |  |  |  |  |  |  |  |  |  |  |  | Minor Flaws |
| Xu et al. (2014) |  |  |  |  |  |  |  |  |  |  |  |  |  |  |  |  |  |  |  |  |  |  |  |  |  |  |  | Major Flaws |
| Cheng et al. (2014) |  |  |  |  |  |  |  |  |  |  |  |  |  |  |  |  |  |  |  |  |  |  |  |  |  |  |  | Major Flaws |
| VianaI and Ignotti (2013) |  |  |  |  |  |  |  |  |  |  |  |  |  |  |  |  |  |  |  |  |  |  |  |  |  |  |  | Major Flaws |
| Burkart et al. (2014) |  |  |  |  |  |  |  |  |  |  |  |  |  |  |  |  |  |  |  |  |  |  |  |  |  |  |  | Major Flaws |
| Poursafa et al. (2015) |  |  |  |  |  |  |  |  |  |  |  |  |  |  |  |  |  |  |  |  |  |  |  |  |  |  |  | Major Flaws |
| Anenberg et al. (2020) |  |  |  |  |  |  |  |  |  |  |  |  |  |  |  |  |  |  |  |  |  |  |  |  |  |  |  | Minor Flaws |
| Chen et al. (2017) |  |  |  |  |  |  |  |  |  |  |  |  |  |  |  |  |  |  |  |  |  |  |  |  |  |  |  | Minimal Flaws |
| Li et al. (2017) |  |  |  |  |  |  |  |  |  |  |  |  |  |  |  |  |  |  |  |  |  |  |  |  |  |  |  | Minimal Flaws |
| Zang et al. (2022) |  |  |  |  |  |  |  |  |  |  |  |  |  |  |  |  |  |  |  |  |  |  |  |  |  |  |  | Minimal Flaws |
| Yee et al. (2021) |  |  |  |  |  |  |  |  |  |  |  |  |  |  |  |  |  |  |  |  |  |  |  |  |  |  |  | Minimal Flaws |
| Niu et al. (2021) |  |  |  |  |  |  |  |  |  |  |  |  |  |  |  |  |  |  |  |  |  |  |  |  |  |  |  | Minimal Flaws |
| Katoto et al. (2021) |  |  |  |  |  |  |  |  |  |  |  |  |  |  |  |  |  |  |  |  |  |  |  |  |  |  |  | Minimal Flaws |
| Hu et al. (2022) |  |  |  |  |  |  |  |  |  |  |  |  |  |  |  |  |  |  |  |  |  |  |  |  |  |  |  | Minimal Flaws |
| Zheng et al. (2021) |  |  |  |  |  |  |  |  |  |  |  |  |  |  |  |  |  |  |  |  |  |  |  |  |  |  |  | Minor Flaws |
| Liu et al. (2021） |  |  |  |  |  |  |  |  |  |  |  |  |  |  |  |  |  |  |  |  |  |  |  |  |  |  |  | Minimal Flaws |
| Walter et al. (2021) |  |  |  |  |  |  |  |  |  |  |  |  |  |  |  |  |  |  |  |  |  |  |  |  |  |  |  | Minor Flaws |
| Yu et al. (2021) |  |  |  |  |  |  |  |  |  |  |  |  |  |  |  |  |  |  |  |  |  |  |  |  |  |  |  | Minor Flaws |
| Huang et al. (2021) |  |  |  |  |  |  |  |  |  |  |  |  |  |  |  |  |  |  |  |  |  |  |  |  |  |  |  | Minimal Flaws |
| Davoudi et al. (2021) |  |  |  |  |  |  |  |  |  |  |  |  |  |  |  |  |  |  |  |  |  |  |  |  |  |  |  | Minimal Flaws |
| Zhang et al. (2021). |  |  |  |  |  |  |  |  |  |  |  |  |  |  |  |  |  |  |  |  |  |  |  |  |  |  |  | Minimal Flaws |
| Ciabattini et al. (2021) |  |  |  |  |  |  |  |  |  |  |  |  |  |  |  |  |  |  |  |  |  |  |  |  |  |  |  | Minimal Flaws |
| Orellano et al. (2021) |  |  |  |  |  |  |  |  |  |  |  |  |  |  |  |  |  |  |  |  |  |  |  |  |  |  |  | Minimal Flaws |
| Zheng et al. (2021) |  |  |  |  |  |  |  |  |  |  |  |  |  |  |  |  |  |  |  |  |  |  |  |  |  |  |  | Minimal Flaws |
| Maleki et al. (2021) |  |  |  |  |  |  |  |  |  |  |  |  |  |  |  |  |  |  |  |  |  |  |  |  |  |  |  | Major Flaws |
| Zhu et al. (2021) |  |  |  |  |  |  |  |  |  |  |  |  |  |  |  |  |  |  |  |  |  |  |  |  |  |  |  | Minimal Flaws |
| Park et al. (2021) |  |  |  |  |  |  |  |  |  |  |  |  |  |  |  |  |  |  |  |  |  |  |  |  |  |  |  | Minimal Flaws |
| Stieb et al. (2021) |  |  |  |  |  |  |  |  |  |  |  |  |  |  |  |  |  |  |  |  |  |  |  |  |  |  |  | Minimal Flaws |
| Alexeeff et al. (2021) |  |  |  |  |  |  |  |  |  |  |  |  |  |  |  |  |  |  |  |  |  |  |  |  |  |  |  | Minor Flaws |
| Prueitt et al. (2022) |  |  |  |  |  |  |  |  |  |  |  |  |  |  |  |  |  |  |  |  |  |  |  |  |  |  |  | Major Flaws |
| MEO et al. (2021) |  |  |  |  |  |  |  |  |  |  |  |  |  |  |  |  |  |  |  |  |  |  |  |  |  |  |  | Major Flaws |
| Zhang et al. (2021) |  |  |  |  |  |  |  |  |  |  |  |  |  |  |  |  |  |  |  |  |  |  |  |  |  |  |  | Minimal Flaws |
| Ning et al. (2021) |  |  |  |  |  |  |  |  |  |  |  |  |  |  |  |  |  |  |  |  |  |  |  |  |  |  |  | Minimal Flaws |
| Xiang et al. (2021) |  |  |  |  |  |  |  |  |  |  |  |  |  |  |  |  |  |  |  |  |  |  |  |  |  |  |  | Minimal Flaws |
| Zou et al. (2021) |  |  |  |  |  |  |  |  |  |  |  |  |  |  |  |  |  |  |  |  |  |  |  |  |  |  |  | Minimal Flaws |
| Trushna et al. (2021) |  |  |  |  |  |  |  |  |  |  |  |  |  |  |  |  |  |  |  |  |  |  |  |  |  |  |  | Minimal Flaws |
| Chen et al. (2021) |  |  |  |  |  |  |  |  |  |  |  |  |  |  |  |  |  |  |  |  |  |  |  |  |  |  |  | Minor Flaws |
| Zhao et al. (2021) |  |  |  |  |  |  |  |  |  |  |  |  |  |  |  |  |  |  |  |  |  |  |  |  |  |  |  | Minimal Flaws |
| Ma et al. (2021). |  |  |  |  |  |  |  |  |  |  |  |  |  |  |  |  |  |  |  |  |  |  |  |  |  |  |  | Minimal Flaws |
| Yue et al. (2021) |  |  |  |  |  |  |  |  |  |  |  |  |  |  |  |  |  |  |  |  |  |  |  |  |  |  |  | Minimal Flaws |
| Noorimotlagh et al. (2021) |  |  |  |  |  |  |  |  |  |  |  |  |  |  |  |  |  |  |  |  |  |  |  |  |  |  |  | Minor Flaws |
| Ibrahim et al. (2021) |  |  |  |  |  |  |  |  |  |  |  |  |  |  |  |  |  |  |  |  |  |  |  |  |  |  |  | Minor Flaws |
| Lederer et al. (2021) |  |  |  |  |  |  |  |  |  |  |  |  |  |  |  |  |  |  |  |  |  |  |  |  |  |  |  | Major Flaws |
| Ghosh et al. (2021) |  |  |  |  |  |  |  |  |  |  |  |  |  |  |  |  |  |  |  |  |  |  |  |  |  |  |  | Minimal Flaws |
| Shahrbaf et al. (2021) |  |  |  |  |  |  |  |  |  |  |  |  |  |  |  |  |  |  |  |  |  |  |  |  |  |  |  | Major Flaws |
| Bernardini et al. (2020) |  |  |  |  |  |  |  |  |  |  |  |  |  |  |  |  |  |  |  |  |  |  |  |  |  |  |  | Major Flaws |
| Xie et al. (2021) |  |  |  |  |  |  |  |  |  |  |  |  |  |  |  |  |  |  |  |  |  |  |  |  |  |  |  | Minimal Flaws |
| Lin et al. (2021) |  |  |  |  |  |  |  |  |  |  |  |  |  |  |  |  |  |  |  |  |  |  |  |  |  |  |  | Minimal Flaws |
| Ma et al. (2021） |  |  |  |  |  |  |  |  |  |  |  |  |  |  |  |  |  |  |  |  |  |  |  |  |  |  |  | Minimal Flaws |
| Wang et al. (2021) |  |  |  |  |  |  |  |  |  |  |  |  |  |  |  |  |  |  |  |  |  |  |  |  |  |  |  | Minimal Flaws |
| Wang et al. (2021) |  |  |  |  |  |  |  |  |  |  |  |  |  |  |  |  |  |  |  |  |  |  |  |  |  |  |  | Minor Flaws |
| Yu et al. (2021) |  |  |  |  |  |  |  |  |  |  |  |  |  |  |  |  |  |  |  |  |  |  |  |  |  |  |  | Minimal Flaws |
| Wu et al. (2022) |  |  |  |  |  |  |  |  |  |  |  |  |  |  |  |  |  |  |  |  |  |  |  |  |  |  |  | Minimal Flaws |
| Uwak et al. (2021) |  |  |  |  |  |  |  |  |  |  |  |  |  |  |  |  |  |  |  |  |  |  |  |  |  |  |  | Minor Flaws |
| Farhadi et al. (2020) |  |  |  |  |  |  |  |  |  |  |  |  |  |  |  |  |  |  |  |  |  |  |  |  |  |  |  | Minimal Flaws |
| Sun et al. (2020) |  |  |  |  |  |  |  |  |  |  |  |  |  |  |  |  |  |  |  |  |  |  |  |  |  |  |  | Minimal Flaws |
| Bai et al. (2020) |  |  |  |  |  |  |  |  |  |  |  |  |  |  |  |  |  |  |  |  |  |  |  |  |  |  |  | Minor Flaws |
| Kim et al. (2020) |  |  |  |  |  |  |  |  |  |  |  |  |  |  |  |  |  |  |  |  |  |  |  |  |  |  |  | Minimal Flaws |
| Harari et al. (2020) |  |  |  |  |  |  |  |  |  |  |  |  |  |  |  |  |  |  |  |  |  |  |  |  |  |  |  | Major Flaws |
| Abed Al Ahad et al. (2020) |  |  |  |  |  |  |  |  |  |  |  |  |  |  |  |  |  |  |  |  |  |  |  |  |  |  |  | Major Flaws |
| Villeneuve et al. (2020) |  |  |  |  |  |  |  |  |  |  |  |  |  |  |  |  |  |  |  |  |  |  |  |  |  |  |  | Major Flaws |
| Wang et al. (2020). |  |  |  |  |  |  |  |  |  |  |  |  |  |  |  |  |  |  |  |  |  |  |  |  |  |  |  | Minimal Flaws |
| Yang et al. (2020) |  |  |  |  |  |  |  |  |  |  |  |  |  |  |  |  |  |  |  |  |  |  |  |  |  |  |  | Minimal Flaws |
| Amiri et al. (2021) |  |  |  |  |  |  |  |  |  |  |  |  |  |  |  |  |  |  |  |  |  |  |  |  |  |  |  | Major Flaws |
| Dimitrova et al. (2021) |  |  |  |  |  |  |  |  |  |  |  |  |  |  |  |  |  |  |  |  |  |  |  |  |  |  |  | Minimal Flaws |
| Kakaei et al. (2021) |  |  |  |  |  |  |  |  |  |  |  |  |  |  |  |  |  |  |  |  |  |  |  |  |  |  |  | Minor Flaws |
| Frangione et al. (2022) |  |  |  |  |  |  |  |  |  |  |  |  |  |  |  |  |  |  |  |  |  |  |  |  |  |  |  | Minor Flaws |
| Heo et al. (2021) |  |  |  |  |  |  |  |  |  |  |  |  |  |  |  |  |  |  |  |  |  |  |  |  |  |  |  | Major Flaws |
| Liu et al. (2021) |  |  |  |  |  |  |  |  |  |  |  |  |  |  |  |  |  |  |  |  |  |  |  |  |  |  |  | Minimal Flaws |
| Ray et al. (2021) |  |  |  |  |  |  |  |  |  |  |  |  |  |  |  |  |  |  |  |  |  |  |  |  |  |  |  | Minimal Flaws |
| Moon (2021) |  |  |  |  |  |  |  |  |  |  |  |  |  |  |  |  |  |  |  |  |  |  |  |  |  |  |  | Minimal Flaws |
| Romero Starke et al. (2021) |  |  |  |  |  |  |  |  |  |  |  |  |  |  |  |  |  |  |  |  |  |  |  |  |  |  |  | Minor Flaws |
| Sexton et al. (2021) |  |  |  |  |  |  |  |  |  |  |  |  |  |  |  |  |  |  |  |  |  |  |  |  |  |  |  | Minimal Flaws |
| Song et al. (2021) |  |  |  |  |  |  |  |  |  |  |  |  |  |  |  |  |  |  |  |  |  |  |  |  |  |  |  | Minor Flaws |
| Weilnhammer et al. (2021) |  |  |  |  |  |  |  |  |  |  |  |  |  |  |  |  |  |  |  |  |  |  |  |  |  |  |  | Minor Flaws |
| Zafeiratou et al. (2021) |  |  |  |  |  |  |  |  |  |  |  |  |  |  |  |  |  |  |  |  |  |  |  |  |  |  |  | Minimal Flaws |
| Liang et al. (2021) |  |  |  |  |  |  |  |  |  |  |  |  |  |  |  |  |  |  |  |  |  |  |  |  |  |  |  | Major Flaws |
| Li et al. (2020) |  |  |  |  |  |  |  |  |  |  |  |  |  |  |  |  |  |  |  |  |  |  |  |  |  |  |  | Minor Flaws |
| Grigorieva and Lukyanets (2021) |  |  |  |  |  |  |  |  |  |  |  |  |  |  |  |  |  |  |  |  |  |  |  |  |  |  |  | Minimal Flaws |
| Areal et al. (2022) |  |  |  |  |  |  |  |  |  |  |  |  |  |  |  |  |  |  |  |  |  |  |  |  |  |  |  | Minor Flaws |
| Song et al. (2022) |  |  |  |  |  |  |  |  |  |  |  |  |  |  |  |  |  |  |  |  |  |  |  |  |  |  |  | Minimal Flaws |
| Chandra et al. (2022) |  |  |  |  |  |  |  |  |  |  |  |  |  |  |  |  |  |  |  |  |  |  |  |  |  |  |  | Minor Flaws |
| Huang et al. (2022) |  |  |  |  |  |  |  |  |  |  |  |  |  |  |  |  |  |  |  |  |  |  |  |  |  |  |  | Minor Flaws |
| Smaller et al. (2022) |  |  |  |  |  |  |  |  |  |  |  |  |  |  |  |  |  |  |  |  |  |  |  |  |  |  |  | Minor Flaws |
| Yu et al. (2022) |  |  |  |  |  |  |  |  |  |  |  |  |  |  |  |  |  |  |  |  |  |  |  |  |  |  |  | Minor Flaws |
| Hu et al. (2022) |  |  |  |  |  |  |  |  |  |  |  |  |  |  |  |  |  |  |  |  |  |  |  |  |  |  |  | Minimal Flaws |
| Ziou et al. (2022) |  |  |  |  |  |  |  |  |  |  |  |  |  |  |  |  |  |  |  |  |  |  |  |  |  |  |  | Minimal Flaws |
| Zhe Sun et al. (2022) |  |  |  |  |  |  |  |  |  |  |  |  |  |  |  |  |  |  |  |  |  |  |  |  |  |  |  | Minimal Flaws |
| Markozannes et al. (2022) |  |  |  |  |  |  |  |  |  |  |  |  |  |  |  |  |  |  |  |  |  |  |  |  |  |  |  | Minor Flaws |
| Liu et al. (2022) |  |  |  |  |  |  |  |  |  |  |  |  |  |  |  |  |  |  |  |  |  |  |  |  |  |  |  | Minimal Flaws |
| Perry et al. (2022) |  |  |  |  |  |  |  |  |  |  |  |  |  |  |  |  |  |  |  |  |  |  |  |  |  |  |  | Minor Flaws |
| Jia et al. (2022) |  |  |  |  |  |  |  |  |  |  |  |  |  |  |  |  |  |  |  |  |  |  |  |  |  |  |  | Minimal Flaws |
| Wang et al. (2022) |  |  |  |  |  |  |  |  |  |  |  |  |  |  |  |  |  |  |  |  |  |  |  |  |  |  |  | Minimal Flaws |
| Bont et al. (2022) |  |  |  |  |  |  |  |  |  |  |  |  |  |  |  |  |  |  |  |  |  |  |  |  |  |  |  | Minor Flaws |
| Chen et al. (2022) |  |  |  |  |  |  |  |  |  |  |  |  |  |  |  |  |  |  |  |  |  |  |  |  |  |  |  | Major Flaws |
| Li et al. (2022) |  |  |  |  |  |  |  |  |  |  |  |  |  |  |  |  |  |  |  |  |  |  |  |  |  |  |  | Minimal Flaws |
| Heo et al. (2022) |  |  |  |  |  |  |  |  |  |  |  |  |  |  |  |  |  |  |  |  |  |  |  |  |  |  |  | Minimal Flaws |
| Pritchett et al. (2022) |  |  |  |  |  |  |  |  |  |  |  |  |  |  |  |  |  |  |  |  |  |  |  |  |  |  |  | Minimal Flaws |
| Zhang et al. (2022) |  |  |  |  |  |  |  |  |  |  |  |  |  |  |  |  |  |  |  |  |  |  |  |  |  |  |  | Minimal Flaws |
| Lin et al. (2022) |  |  |  |  |  |  |  |  |  |  |  |  |  |  |  |  |  |  |  |  |  |  |  |  |  |  |  | Minimal Flaws |
| Guo et al. (2022) |  |  |  |  |  |  |  |  |  |  |  |  |  |  |  |  |  |  |  |  |  |  |  |  |  |  |  | Minimal Flaws |
| Yang et al. (2022) |  |  |  |  |  |  |  |  |  |  |  |  |  |  |  |  |  |  |  |  |  |  |  |  |  |  |  | Minor Flaws |
| Khosravipour et al. (2022) |  |  |  |  |  |  |  |  |  |  |  |  |  |  |  |  |  |  |  |  |  |  |  |  |  |  |  | Minimal Flaws |
| Zang et al. (2022) |  |  |  |  |  |  |  |  |  |  |  |  |  |  |  |  |  |  |  |  |  |  |  |  |  |  |  | Minimal Flaws |
| Rasking et al. (2022) |  |  |  |  |  |  |  |  |  |  |  |  |  |  |  |  |  |  |  |  |  |  |  |  |  |  |  | Minor Flaws |
| Dimala et al. (2022) |  |  |  |  |  |  |  |  |  |  |  |  |  |  |  |  |  |  |  |  |  |  |  |  |  |  |  | Minimal Flaws |
| Guo et al. (2022) |  |  |  |  |  |  |  |  |  |  |  |  |  |  |  |  |  |  |  |  |  |  |  |  |  |  |  | Minimal Flaws |
| Holm et al. (2021) |  |  |  |  |  |  |  |  |  |  |  |  |  |  |  |  |  |  |  |  |  |  |  |  |  |  |  | Minor Flaws |
| Lin et al. (2022) |  |  |  |  |  |  |  |  |  |  |  |  |  |  |  |  |  |  |  |  |  |  |  |  |  |  |  | Minor Flaws |
| Xu et al. (2022) |  |  |  |  |  |  |  |  |  |  |  |  |  |  |  |  |  |  |  |  |  |  |  |  |  |  |  | Minimal Flaws |
| Yue et al. (2022) |  |  |  |  |  |  |  |  |  |  |  |  |  |  |  |  |  |  |  |  |  |  |  |  |  |  |  | Minimal Flaws |
| Manullang et al. (2022) |  |  |  |  |  |  |  |  |  |  |  |  |  |  |  |  |  |  |  |  |  |  |  |  |  |  |  | Minor Flaws |
| Gasana et al. (2012) |  |  |  |  |  |  |  |  |  |  |  |  |  |  |  |  |  |  |  |  |  |  |  |  |  |  |  | Minor Flaws |
| Liu et al. (2023) |  |  |  |  |  |  |  |  |  |  |  |  |  |  |  |  |  |  |  |  |  |  |  |  |  |  |  | Major Flaws |
| Mason et al. (2022) |  |  |  |  |  |  |  |  |  |  |  |  |  |  |  |  |  |  |  |  |  |  |  |  |  |  |  | Minimal Flaws |
| Arsad et al. (2022) |  |  |  |  |  |  |  |  |  |  |  |  |  |  |  |  |  |  |  |  |  |  |  |  |  |  |  | Minor Flaws |
| Faurie et al. (2022) |  |  |  |  |  |  |  |  |  |  |  |  |  |  |  |  |  |  |  |  |  |  |  |  |  |  |  | Minimal Flaws |
| Han et al. (2023) |  |  |  |  |  |  |  |  |  |  |  |  |  |  |  |  |  |  |  |  |  |  |  |  |  |  |  | Minimal Flaws |
| Benmarhnia et al. (2015) |  |  |  |  |  |  |  |  |  |  |  |  |  |  |  |  |  |  |  |  |  |  |  |  |  |  |  | Minimal Flaws |
| Cong et al, (2017) |  |  |  |  |  |  |  |  |  |  |  |  |  |  |  |  |  |  |  |  |  |  |  |  |  |  |  | Minor Flaws |
| Lakhoo et al. (2022) |  |  |  |  |  |  |  |  |  |  |  |  |  |  |  |  |  |  |  |  |  |  |  |  |  |  |  | Minor Flaws |
| Liu et al. (2021) |  |  |  |  |  |  |  |  |  |  |  |  |  |  |  |  |  |  |  |  |  |  |  |  |  |  |  | Minimal Flaws |
| Zheng et al. (2021) |  |  |  |  |  |  |  |  |  |  |  |  |  |  |  |  |  |  |  |  |  |  |  |  |  |  |  | Minor Flaws |
| Wang et al. (2021) |  |  |  |  |  |  |  |  |  |  |  |  |  |  |  |  |  |  |  |  |  |  |  |  |  |  |  | Major Flaws |
| Wu et al. (2022) |  |  |  |  |  |  |  |  |  |  |  |  |  |  |  |  |  |  |  |  |  |  |  |  |  |  |  | Minimal Flaws |
| Islam and Noor (2022) |  |  |  |  |  |  |  |  |  |  |  |  |  |  |  |  |  |  |  |  |  |  |  |  |  |  |  | Minimal Flaws |
| Wang et al. (2022) |  |  |  |  |  |  |  |  |  |  |  |  |  |  |  |  |  |  |  |  |  |  |  |  |  |  |  | Minimal Flaws |
| Manyuchi et al. (2022) |  |  |  |  |  |  |  |  |  |  |  |  |  |  |  |  |  |  |  |  |  |  |  |  |  |  |  | Major Flaws |
| Lee et al. (2019) |  |  |  |  |  |  |  |  |  |  |  |  |  |  |  |  |  |  |  |  |  |  |  |  |  |  |  | Minor Flaws |
| Gao et al. (2022) |  |  |  |  |  |  |  |  |  |  |  |  |  |  |  |  |  |  |  |  |  |  |  |  |  |  |  | Minor Flaws |
| Krittanawong et al. (2023) |  |  |  |  |  |  |  |  |  |  |  |  |  |  |  |  |  |  |  |  |  |  |  |  |  |  |  | Minor Flaws |
| Pyo et al. (2022) |  |  |  |  |  |  |  |  |  |  |  |  |  |  |  |  |  |  |  |  |  |  |  |  |  |  |  | Minor Flaws |
| Hernandez Carballo et al, (2022) |  |  |  |  |  |  |  |  |  |  |  |  |  |  |  |  |  |  |  |  |  |  |  |  |  |  |  | Minor Flaws |
| Rezayat et al. (2022) |  |  |  |  |  |  |  |  |  |  |  |  |  |  |  |  |  |  |  |  |  |  |  |  |  |  |  | Minimal Flaws |
| Gan et al. (2023) |  |  |  |  |  |  |  |  |  |  |  |  |  |  |  |  |  |  |  |  |  |  |  |  |  |  |  | Minor Flaws |
| Juneja Gandhi et al. (2022) |  |  |  |  |  |  |  |  |  |  |  |  |  |  |  |  |  |  |  |  |  |  |  |  |  |  |  | Minor Flaws |
| Badida et al. (2023) |  |  |  |  |  |  |  |  |  |  |  |  |  |  |  |  |  |  |  |  |  |  |  |  |  |  |  | Minimal Flaws |
| Zhang et al. (2023) |  |  |  |  |  |  |  |  |  |  |  |  |  |  |  |  |  |  |  |  |  |  |  |  |  |  |  | Minimal Flaws |
| Podury et al. (2023) |  |  |  |  |  |  |  |  |  |  |  |  |  |  |  |  |  |  |  |  |  |  |  |  |  |  |  | Minor Flaws |
| Wang and Cao (2022) |  |  |  |  |  |  |  |  |  |  |  |  |  |  |  |  |  |  |  |  |  |  |  |  |  |  |  | Minimal Flaws |
| Ju et al. (2023) |  |  |  |  |  |  |  |  |  |  |  |  |  |  |  |  |  |  |  |  |  |  |  |  |  |  |  | Minimal Flaws |
| Luo et al. (2023) |  |  |  |  |  |  |  |  |  |  |  |  |  |  |  |  |  |  |  |  |  |  |  |  |  |  |  | Minor Flaws |
| Zhu et al. (2023) |  |  |  |  |  |  |  |  |  |  |  |  |  |  |  |  |  |  |  |  |  |  |  |  |  |  |  | Minimal Flaws |
| Yang et al. (2022) |  |  |  |  |  |  |  |  |  |  |  |  |  |  |  |  |  |  |  |  |  |  |  |  |  |  |  | Minimal Flaws |
| Yang et al. (2023) |  |  |  |  |  |  |  |  |  |  |  |  |  |  |  |  |  |  |  |  |  |  |  |  |  |  |  | Minor Flaws |
| Chung et al. (2022) |  |  |  |  |  |  |  |  |  |  |  |  |  |  |  |  |  |  |  |  |  |  |  |  |  |  |  | Minimal Flaws |
| Husaini et al, (2022) |  |  |  |  |  |  |  |  |  |  |  |  |  |  |  |  |  |  |  |  |  |  |  |  |  |  |  | Minor Flaws |
| Ruan and Zeng (2023) |  |  |  |  |  |  |  |  |  |  |  |  |  |  |  |  |  |  |  |  |  |  |  |  |  |  |  | Minimal Flaws |
| Tabaei et al, (2023) |  |  |  |  |  |  |  |  |  |  |  |  |  |  |  |  |  |  |  |  |  |  |  |  |  |  |  | Minor Flaws |
| Zhang et al. (2022) |  |  |  |  |  |  |  |  |  |  |  |  |  |  |  |  |  |  |  |  |  |  |  |  |  |  |  | Minimal Flaws |
| Zhang et al. (2022) |  |  |  |  |  |  |  |  |  |  |  |  |  |  |  |  |  |  |  |  |  |  |  |  |  |  |  | Minimal Flaws |
| Sui et al. (2022) |  |  |  |  |  |  |  |  |  |  |  |  |  |  |  |  |  |  |  |  |  |  |  |  |  |  |  | Minimal Flaws |
| Ngoc et al. (2017) |  |  |  |  |  |  |  |  |  |  |  |  |  |  |  |  |  |  |  |  |  |  |  |  |  |  |  | Major Flaws |
| Fu et al. (2019) |  |  |  |  |  |  |  |  |  |  |  |  |  |  |  |  |  |  |  |  |  |  |  |  |  |  |  | Minimal Flaws |
| Han et al. (2019) |  |  |  |  |  |  |  |  |  |  |  |  |  |  |  |  |  |  |  |  |  |  |  |  |  |  |  | Minimal Flaws |
| Hu et al. (2019) |  |  |  |  |  |  |  |  |  |  |  |  |  |  |  |  |  |  |  |  |  |  |  |  |  |  |  | Minimal Flaws |
| Ohlwein et al. (2019) |  |  |  |  |  |  |  |  |  |  |  |  |  |  |  |  |  |  |  |  |  |  |  |  |  |  |  | Major Flaws |
| Oliveira et al. (2019) |  |  |  |  |  |  |  |  |  |  |  |  |  |  |  |  |  |  |  |  |  |  |  |  |  |  |  | Major Flaws |
| Tsoli et al. (2019) |  |  |  |  |  |  |  |  |  |  |  |  |  |  |  |  |  |  |  |  |  |  |  |  |  |  |  | Major Flaws |
| Fan et al. (2020) |  |  |  |  |  |  |  |  |  |  |  |  |  |  |  |  |  |  |  |  |  |  |  |  |  |  |  | Minimal Flaws |
| Huang et al. (2020) |  |  |  |  |  |  |  |  |  |  |  |  |  |  |  |  |  |  |  |  |  |  |  |  |  |  |  | Minimal Flaws |
| Samoli et al. (2020) |  |  |  |  |  |  |  |  |  |  |  |  |  |  |  |  |  |  |  |  |  |  |  |  |  |  |  | Minimal Flaws |
| Yan et al. (2020) |  |  |  |  |  |  |  |  |  |  |  |  |  |  |  |  |  |  |  |  |  |  |  |  |  |  |  | Minimal Flaws |
| Yu et al. (2020) |  |  |  |  |  |  |  |  |  |  |  |  |  |  |  |  |  |  |  |  |  |  |  |  |  |  |  | Minimal Flaws |
| Huang et al. (2021) |  |  |  |  |  |  |  |  |  |  |  |  |  |  |  |  |  |  |  |  |  |  |  |  |  |  |  | Minimal Flaws |
| Huang et al. (2021) |  |  |  |  |  |  |  |  |  |  |  |  |  |  |  |  |  |  |  |  |  |  |  |  |  |  |  | Minimal Flaws |
| Ju et al. (2021). |  |  |  |  |  |  |  |  |  |  |  |  |  |  |  |  |  |  |  |  |  |  |  |  |  |  |  | Minimal Flaws |
| Lin et al. (2021) |  |  |  |  |  |  |  |  |  |  |  |  |  |  |  |  |  |  |  |  |  |  |  |  |  |  |  | Minimal Flaws |
| Liu et al. (2021) |  |  |  |  |  |  |  |  |  |  |  |  |  |  |  |  |  |  |  |  |  |  |  |  |  |  |  | Minimal Flaws |
| McDermott-Levy et al. (2021) |  |  |  |  |  |  |  |  |  |  |  |  |  |  |  |  |  |  |  |  |  |  |  |  |  |  |  | Major Flaws |
| Ni et al. (2021) |  |  |  |  |  |  |  |  |  |  |  |  |  |  |  |  |  |  |  |  |  |  |  |  |  |  |  | Minimal Flaws |
| Zang et al. (2021) |  |  |  |  |  |  |  |  |  |  |  |  |  |  |  |  |  |  |  |  |  |  |  |  |  |  |  | Minimal Flaws |
| Borroni et al. (2022) |  |  |  |  |  |  |  |  |  |  |  |  |  |  |  |  |  |  |  |  |  |  |  |  |  |  |  | Minimal Flaws |
| Cheng et al. (2022) |  |  |  |  |  |  |  |  |  |  |  |  |  |  |  |  |  |  |  |  |  |  |  |  |  |  |  | Minimal Flaws |
| Juarez et al. (2022) |  |  |  |  |  |  |  |  |  |  |  |  |  |  |  |  |  |  |  |  |  |  |  |  |  |  |  | Major Flaws |
| Puthota et al. (2022) |  |  |  |  |  |  |  |  |  |  |  |  |  |  |  |  |  |  |  |  |  |  |  |  |  |  |  | Minor Flaws |
| Zhang et al. (2022) |  |  |  |  |  |  |  |  |  |  |  |  |  |  |  |  |  |  |  |  |  |  |  |  |  |  |  | Minimal Flaws |
| Zong et al. (2022) |  |  |  |  |  |  |  |  |  |  |  |  |  |  |  |  |  |  |  |  |  |  |  |  |  |  |  | Minimal Flaws |
| Blanc et al. (2023) |  |  |  |  |  |  |  |  |  |  |  |  |  |  |  |  |  |  |  |  |  |  |  |  |  |  |  | Minor Flaws |
| Bouchriti et al. (2023 |  |  |  |  |  |  |  |  |  |  |  |  |  |  |  |  |  |  |  |  |  |  |  |  |  |  |  | Major Flaws |
| Bougea et al. (2023) |  |  |  |  |  |  |  |  |  |  |  |  |  |  |  |  |  |  |  |  |  |  |  |  |  |  |  | Minor Flaws |
| Fadadu et al. (2023) |  |  |  |  |  |  |  |  |  |  |  |  |  |  |  |  |  |  |  |  |  |  |  |  |  |  |  | Minor Flaws |
| Fan et al. (2023) |  |  |  |  |  |  |  |  |  |  |  |  |  |  |  |  |  |  |  |  |  |  |  |  |  |  |  | Minimal Flaws |
| Guo et al. (2023) |  |  |  |  |  |  |  |  |  |  |  |  |  |  |  |  |  |  |  |  |  |  |  |  |  |  |  | Minimal Flaws |
| Huang et al. (2023) |  |  |  |  |  |  |  |  |  |  |  |  |  |  |  |  |  |  |  |  |  |  |  |  |  |  |  | Minimal Flaws |
| Jia et al. (2023) |  |  |  |  |  |  |  |  |  |  |  |  |  |  |  |  |  |  |  |  |  |  |  |  |  |  |  | Minimal Flaws |
| Karimi and Samadi (2023) |  |  |  |  |  |  |  |  |  |  |  |  |  |  |  |  |  |  |  |  |  |  |  |  |  |  |  | Minimal Flaws |
| Li et al. (2023) |  |  |  |  |  |  |  |  |  |  |  |  |  |  |  |  |  |  |  |  |  |  |  |  |  |  |  | Minimal Flaws |
| Liu et al. (2023) |  |  |  |  |  |  |  |  |  |  |  |  |  |  |  |  |  |  |  |  |  |  |  |  |  |  |  | Minimal Flaws |
| Liu et al. (2023) |  |  |  |  |  |  |  |  |  |  |  |  |  |  |  |  |  |  |  |  |  |  |  |  |  |  |  | Minimal Flaws |
| Liu et al. (2023) |  |  |  |  |  |  |  |  |  |  |  |  |  |  |  |  |  |  |  |  |  |  |  |  |  |  |  | Minimal Flaws |
| Liu et al. (2023) |  |  |  |  |  |  |  |  |  |  |  |  |  |  |  |  |  |  |  |  |  |  |  |  |  |  |  | Minimal Flaws |
| Luben et al. (2023) |  |  |  |  |  |  |  |  |  |  |  |  |  |  |  |  |  |  |  |  |  |  |  |  |  |  |  | Minimal Flaws |
| Pan et al. (2023) |  |  |  |  |  |  |  |  |  |  |  |  |  |  |  |  |  |  |  |  |  |  |  |  |  |  |  | Minimal Flaws |
| Parasin et al. (2023) |  |  |  |  |  |  |  |  |  |  |  |  |  |  |  |  |  |  |  |  |  |  |  |  |  |  |  | Minimal Flaws |
| Sheppard et al. (2023) |  |  |  |  |  |  |  |  |  |  |  |  |  |  |  |  |  |  |  |  |  |  |  |  |  |  |  | Minimal Flaws |
| Tandon et al. (2023) |  |  |  |  |  |  |  |  |  |  |  |  |  |  |  |  |  |  |  |  |  |  |  |  |  |  |  | Minimal Flaws |
| Thompson et al. (2023) |  |  |  |  |  |  |  |  |  |  |  |  |  |  |  |  |  |  |  |  |  |  |  |  |  |  |  | Minimal Flaws |
| Wan et al. (2023) |  |  |  |  |  |  |  |  |  |  |  |  |  |  |  |  |  |  |  |  |  |  |  |  |  |  |  | Minimal Flaws |
| Wang et al. (2023) |  |  |  |  |  |  |  |  |  |  |  |  |  |  |  |  |  |  |  |  |  |  |  |  |  |  |  | Minimal Flaws |
| Wen et al. (2023) |  |  |  |  |  |  |  |  |  |  |  |  |  |  |  |  |  |  |  |  |  |  |  |  |  |  |  | Minimal Flaws |
| Wu et al. (2023) |  |  |  |  |  |  |  |  |  |  |  |  |  |  |  |  |  |  |  |  |  |  |  |  |  |  |  | Minimal Flaws |
| Yu et al. (2023) |  |  |  |  |  |  |  |  |  |  |  |  |  |  |  |  |  |  |  |  |  |  |  |  |  |  |  | Minimal Flaws |
| Yu et al. (2023) |  |  |  |  |  |  |  |  |  |  |  |  |  |  |  |  |  |  |  |  |  |  |  |  |  |  |  | Minimal Flaws |
| Zhai et al. (2023) |  |  |  |  |  |  |  |  |  |  |  |  |  |  |  |  |  |  |  |  |  |  |  |  |  |  |  | Minimal Flaws |
| Zhang et al. (2023) |  |  |  |  |  |  |  |  |  |  |  |  |  |  |  |  |  |  |  |  |  |  |  |  |  |  |  | Minimal Flaws |
