# Supplementary material for: Methodological and reporting quality of systematic reviews on health effects of air pollutants were higher than extreme temperatures: a comparative study
Source: BMC Public Health. 2023 Nov 29;23:2371. doi: 10.1186/s12889-023-17256-5 (PMC10687779; doi:10.1186/s12889-023-17256-5)
Supplement: Supplementary file 2 — Supplementary Material 2 [file 12889_2023_17256_MOESM2_ESM.docx]

**The list of included studies**

1. Requia WJ, Adams MD, Arain A, Papatheodorou S, Koutrakis P, Mahmoud M. Global Association of Air Pollution and Cardiorespiratory Diseases: A Systematic Review, Meta-Analysis, and Investigation of Modifier Variables. *Am J Public Health.* 2018; 108(S2):S123-S130.

2. Stieb DM, Zheng C, Salama D, Berjawi R, Emode M, Hocking R*, et al.* Systematic review and meta-analysis of case-crossover and time-series studies of short term outdoor nitrogen dioxide exposure and ischemic heart disease morbidity. *Environ Health.* 2020; 19(1):47.

3. Ren M, Fang X, Li M, Sun S, Pei L, Xu Q*, et al.* Concentration-Response Relationship between PM2.5 and Daily Respiratory Deaths in China: A Systematic Review and Metaregression Analysis of Time-Series Studies. *Biomed Res Int.* 2017; 2017:5806185.

4. Cui P, Huang Y, Han J, Song F, Chen K. Ambient particulate matter and lung cancer incidence and mortality: a meta-analysis of prospective studies. *Eur J Public Health.* 2015; 25(2):324-329.

5. Glinianaia SV, Rankin J, Bell R, Pless-Mulloli T, Howel D. Does particulate air pollution contribute to infant death? A systematic review. *Environ Health Perspect.* 2004; 112(14):1365-1371.

6. Kotsyfakis M, Zarogiannis SG, Patelarou E. The health impact of Saharan dust exposure. *Int J Occup Med Environ Health.* 2019; 32(6):749-760.

7. Wong EY, Gohlke J, Griffith WC, Farrow S, Faustman EM. Assessing the health benefits of air pollution reduction for children. *Environ Health Perspect.* 2004; 112(2):226-232.

8. Nhung NTT, Amini H, Schindler C, Kutlar Joss M, Dien TM, Probst-Hensch N*, et al.* Short-term association between ambient air pollution and pneumonia in children: A systematic review and meta-analysis of time-series and case-crossover studies. *Environ Pollut.* 2017; 230:1000-1008.

9. Vrijheid M, Martinez D, Manzanares S, Dadvand P, Schembari A, Rankin J*, et al.* Ambient air pollution and risk of congenital anomalies: a systematic review and meta-analysis. *Environ Health Perspect.* 2011; 119(5):598-606.

10. Pedersen M, Stayner L, Slama R, Sorensen M, Figueras F, Nieuwenhuijsen MJ*, et al.* Ambient air pollution and pregnancy-induced hypertensive disorders: a systematic review and meta-analysis. *Hypertension.* 2014; 64(3):494-500.

11. Atkinson RW, Mills IC, Walton HA, Anderson HR. Fine particle components and health--a systematic review and meta-analysis of epidemiological time series studies of daily mortality and hospital admissions. *J Expo Sci Environ Epidemiol.* 2015; 25(2):208-214.

12. Zhang S, Li G, Tian L, Guo Q, Pan X. Short-term exposure to air pollution and morbidity of COPD and asthma in East Asian area: A systematic review and meta-analysis. *Environ Res.* 2016; 148:15-23.

13. Ravindra K, Chanana N, Mor S. Exposure to air pollutants and risk of congenital anomalies: A systematic review and metaanalysis. *Sci Total Environ.* 2021; 765:142772.

14. Newell K, Kartsonaki C, Lam KBH, Kurmi OP. Cardiorespiratory health effects of particulate ambient air pollution exposure in low-income and middle-income countries: a systematic review and meta-analysis. *The Lancet Planetary Health.* 2017; 1(9):e368-e380.

15. Braithwaite I, Zhang S, Kirkbride JB, Osborn DPJ, Hayes JF. Air Pollution (Particulate Matter) Exposure and Associations with Depression, Anxiety, Bipolar, Psychosis and Suicide Risk: A Systematic Review and Meta-Analysis. *Environ Health Perspect.* 2019; 127(12):126002.

16. Orellano P, Quaranta N, Reynoso J, Balbi B, Vasquez J. Effect of outdoor air pollution on asthma exacerbations in children and adults: Systematic review and multilevel meta-analysis. *PLoS One.* 2017; 12(3):e0174050.

17. Chen EK, Zmirou-Navier D, Padilla C, Deguen S. Effects of air pollution on the risk of congenital anomalies: a systematic review and meta-analysis. *Int J Environ Res Public Health.* 2014; 11(8):7642-7668.

18. Song Q, Christiani DC, XiaorongWang, Ren J. The global contribution of outdoor air pollution to the incidence, prevalence, mortality and hospital admission for chronic obstructive pulmonary disease: a systematic review and meta-analysis. *Int J Environ Res Public Health.* 2014; 11(11):11822-11832.

19. Yang BY, Fan S, Thiering E, Seissler J, Nowak D, Dong GH*, et al.* Ambient air pollution and diabetes: A systematic review and meta-analysis. *Environ Res.* 2020; 180:108817.

20. Song X, Liu Y, Hu Y, Zhao X, Tian J, Ding G*, et al.* Short-Term Exposure to Air Pollution and Cardiac Arrhythmia: A Meta-Analysis and Systematic Review. *Int J Environ Res Public Health.* 2016; 13(7).

21. Shah ASV, Langrish JP, Nair H, McAllister DA, Hunter AL, Donaldson K*, et al.* Global association of air pollution and heart failure: a systematic review and meta-analysis. *The Lancet.* 2013; 382(9897):1039-1048.

22. Newell K, Kartsonaki C, Lam KBH, Kurmi O. Cardiorespiratory health effects of gaseous ambient air pollution exposure in low and middle income countries: a systematic review and meta-analysis. *Environ Health.* 2018; 17(1):41.

23. Kim HB, Shim JY, Park B, Lee YJ. Long-Term Exposure to Air Pollutants and Cancer Mortality: A Meta-Analysis of Cohort Studies. *Int J Environ Res Public Health.* 2018; 15(11).

24. Kihal-Talantikite W, Marchetta GP, Deguen S. Infant Mortality Related to NO2 and PM Exposure: Systematic Review and Meta-Analysis. *Int J Environ Res Public Health.* 2020; 17(8).

25. Zhao L, Liang H, Chen F, Chen Z, Guan W, Li J. Association between air pollution and cardiovascular mortality in China: a systematic review and meta-analysis. *Oncotarget.* 2017; 8(39):66438-66448.

26. Scheers H, Jacobs L, Casas L, Nemery B, Nawrot TS. Long-Term Exposure to Particulate Matter Air Pollution Is a Risk Factor for Stroke: Meta-Analytical Evidence. *Stroke.* 2015; 46(11):3058-3066.

27. Siddika N, Balogun HA, Amegah AK, Jaakkola JJ. Prenatal ambient air pollution exposure and the risk of stillbirth: systematic review and meta-analysis of the empirical evidence. *Occup Environ Med.* 2016; 73(9):573-581.

28. Liu B, Fan D, Huang F. Relationship of chronic kidney disease with major air pollutants - A systematic review and meta-analysis of observational studies. *Environ Toxicol Pharmacol.* 2020; 76:103355.

29. Shah AS, Lee KK, McAllister DA, Hunter A, Nair H, Whiteley W*, et al.* Short term exposure to air pollution and stroke: systematic review and meta-analysis. *BMJ.* 2015; 350:h1295.

30. Chang X, Zhou L, Tang M, Wang B. Association of fine particles with respiratory disease mortality: a meta-analysis. *Arch Environ Occup Health.* 2015; 70(2):98-101.

31. Shang Y, Sun Z, Cao J, Wang X, Zhong L, Bi X*, et al.* Systematic review of Chinese studies of short-term exposure to air pollution and daily mortality. *Environ Int.* 2013; 54:100-111.

32. Li C, Fang D, Xu D, Wang B, Zhao S, Yan S*, et al.* Main air pollutants and diabetes-associated mortality: a systematic review and meta-analysis. *Eur J Endocrinol.* 2014; 171(5):R183-190.

33. Yang WS, Zhao H, Wang X, Deng Q, Fan WY, Wang L. An evidence-based assessment for the association between long-term exposure to outdoor air pollution and the risk of lung cancer. *Eur J Cancer Prev.* 2016; 25(3):163-172.

34. Bekkar B, Pacheco S, Basu R, DeNicola N. Association of Air Pollution and Heat Exposure With Preterm Birth, Low Birth Weight, and Stillbirth in the US: A Systematic Review. *JAMA Netw Open.* 2020; 3(6):e208243.

35. Yang WS, Wang X, Deng Q, Fan WY, Wang WY. An evidence-based appraisal of global association between air pollution and risk of stroke. *Int J Cardiol.* 2014; 175(2):307-313.

36. Lai H, Tsang H, Wong C. Meta-analysis of adverse health effects due to air pollution in Chinese populations. *BMC Public Health.* 2013; 13(360).

37. Huangfu P, Atkinson R. Long-term exposure to NO2 and O3 and all-cause and respiratory mortality: A systematic review and meta-analysis. *Environ Int.* 2020; 144:105998.

38. DeVries R, Kriebel D, Sama S. Outdoor Air Pollution and COPD-Related Emergency Department Visits, Hospital Admissions, and Mortality: A Meta-Analysis. *COPD.* 2017; 14(1):113-121.

39. Vodonos A, Awad YA, Schwartz J. The concentration-response between long-term PM2.5 exposure and mortality; A meta-regression approach. *Environ Res.* 2018; 166:677-689.

40. Yu XB, Su JW, Li XY, Chen G. Short-term effects of particulate matter on stroke attack: meta-regression and meta-analyses. *PLoS One.* 2014; 9(5):e95682.

41. Gao H, Wang K, W WA, Zhao W, Xia ZL. A Systematic Review and Meta-Analysis of Short-Term Ambient Ozone Exposure and COPD Hospitalizations. *Int J Environ Res Public Health.* 2020; 17(6).

42. Wang K, Hao Y, Au W, Christiani DC, Xia ZL. A Systematic Review and Meta-Analysis on Short-Term Particulate Matter Exposure and Chronic Obstructive Pulmonary Disease Hospitalizations in China. *J Occup Environ Med.* 2019; 61(4):e112-e124.

43. Wang Y, Eliot MN, Wellenius GA. Short-term changes in ambient particulate matter and risk of stroke: a systematic review and meta-analysis. *J Am Heart Assoc.* 2014; 3(4).

44. Chen J, Hoek G. Long-term exposure to PM and all-cause and cause-specific mortality: A systematic review and meta-analysis. *Environ Int.* 2020; 143:105974.

45. Mills IC, Atkinson RW, Anderson HR, Maynard RL, Strachan DP. Distinguishing the associations between daily mortality and hospital admissions and nitrogen dioxide from those of particulate matter: a systematic review and meta-analysis. *BMJ Open.* 2016; 6(7):e010751.

46. Zhu R, Chen Y, Wu S, Deng F, Liu Y, Yao W. The relationship between particulate matter (PM10) and hospitalizations and mortality of chronic obstructive pulmonary disease: a meta-analysis. *COPD.* 2013; 10(3):307-315.

47. Bell ML, Zanobetti A, Dominici F. Evidence on vulnerability and susceptibility to health risks associated with short-term exposure to particulate matter: a systematic review and meta-analysis. *Am J Epidemiol.* 2013; 178(6):865-876.

48. Zare Sakhvidi MJ, Lequy E, Goldberg M, Jacquemin B. Air pollution exposure and bladder, kidney and urinary tract cancer risk: A systematic review. *Environ Pollut.* 2020; 267:115328.

49. Achilleos S, Kioumourtzoglou MA, Wu CD, Schwartz JD, Koutrakis P, Papatheodorou SI. Acute effects of fine particulate matter constituents on mortality: A systematic review and meta-regression analysis. *Environ Int.* 2017; 109:89-100.

50. Hoek G, Krishnan RM, Beelen R, Peters C, Ostro B, Brunekreef B*, et al.* Long-term air pollution exposure and cardio- respiratory mortality: a review. *Environmental Health.* 2013; 12(43).

51. Lee KK, Spath N, Miller MR, Mills NL, Shah ASV. Short-term exposure to carbon monoxide and myocardial infarction: A systematic review and meta-analysis. *Environ Int.* 2020; 143:105901.

52. Zhu RX, Nie XH, Chen YH, Chen J, Wu SW, Zhao LH. Relationship Between Particulate Matter (PM2.5) and Hospitalizations and Mortality of Chronic Obstructive Pulmonary Disease Patients: A Meta-Analysis. *Am J Med Sci.* 2020; 359(6):354-364.

53. Pranata R, Vania R, Tondas AE, Setianto B, Santoso A. A time-to-event analysis on air pollutants with the risk of cardiovascular disease and mortality: A systematic review and meta-analysis of 84 cohort studies. *J Evid Based Med.* 2020; 13(2):102-115.

54. Hamra GB, Guha N, Cohen A, Laden F, Raaschou-Nielsen O, Samet JM*, et al.* Outdoor particulate matter exposure and lung cancer: a systematic review and meta-analysis. *Environ Health Perspect.* 2014; 122(9):906-911.

55. Bell ML, Zanobetti A, Dominici F. Who is more affected by ozone pollution? A systematic review and meta-analysis. *Am J Epidemiol.* 2014; 180(1):15-28.

56. Zhao T, Markevych I, Romanos M, Nowak D, Heinrich J. Ambient ozone exposure and mental health: A systematic review of epidemiological studies. *Environ Res.* 2018; 165:459-472.

57. Fu P, Guo X, Cheung FMH, Yung KKL. The association between PM2.5 exposure and neurological disorders: A systematic review and meta-analysis. *Sci Total Environ.* 2019; 655:1240-1248.

58. Yan M, Liu Z, Liu X, Duan H, Li T. Meta-analysis of the Chinese studies of the association between ambient ozone and mortality. *Chemosphere.* 2013; 93(6):899-905.

59. Zheng XY, Ding H, Jiang LN, Chen SW, Zheng JP, Qiu M*, et al.* Association between Air Pollutants and Asthma Emergency Room Visits and Hospital Admissions in Time Series Studies: A Systematic Review and Meta-Analysis. *PLoS One.* 2015; 10(9):e0138146.

60. Zhang Z, Wang J, Lu W. Exposure to nitrogen dioxide and chronic obstructive pulmonary disease (COPD) in adults: a systematic review and meta-analysis. *Environ Sci Pollut Res Int.* 2018; 25(15):15133-15145.

61. Ito K, De Leon SF, Lippmann M. Associations between ozone and daily mortality: analysis and meta-analysis. *Epidemiology.* 2005; 16(4):446-457.

62. Cai X, Li Z, Scott EM, Li X, Tang M. Short-term effects of atmospheric particulate matter on myocardial infarction: a cumulative meta-analysis. *Environ Sci Pollut Res Int.* 2016; 23(7):6139-6148.

63. Sun J, Barnes AJ, He D, Wang M, Wang J. Systematic Review and Meta-Analysis of the Association between Ambient Nitrogen Dioxide and Respiratory Disease in China. *Int J Environ Res Public Health.* 2017; 14(6).

64. Park HY, Bae S, Hong YC. PM(1)(0) exposure and non-accidental mortality in Asian populations: a meta-analysis of time-series and case-crossover studies. *J Prev Med Public Health.* 2013; 46(1):10-18.

65. Li MH, Fan LC, Mao B, Yang JW, Choi AMK, Cao WJ*, et al.* Short-term Exposure to Ambient Fine Particulate Matter Increases Hospitalizations and Mortality in COPD: A Systematic Review and Meta-analysis. *Chest.* 2016; 149(2):447-458.

66. Yuan S, Wang J, Jiang Q, He Z, Huang Y, Li Z*, et al.* Long-term exposure to PM2.5 and stroke: A systematic review and meta-analysis of cohort studies. *Environ Res.* 2019; 177:108587.

67. Mills IC, Atkinson RW, Kang S, Walton H, Anderson HR. Quantitative systematic review of the associations between short-term exposure to nitrogen dioxide and mortality and hospital admissions. *BMJ Open.* 2015; 5(5):e006946.

68. Huang F, Pan B, Wu J, Chen E, Chen L. Relationship between exposure to PM2.5 and lung cancer incidence and mortality: A meta-analysis. *Oncotarget.* 2017; 8(26):43322-43331.

69. Lu F, Xu D, Cheng Y, Dong S, Guo C, Jiang X*, et al.* Systematic review and meta-analysis of the adverse health effects of ambient PM2.5 and PM10 pollution in the Chinese population. *Environ Res.* 2015; 136:196-204.

70. Orellano P, Reynoso J, Quaranta N, Bardach A, Ciapponi A. Short-term exposure to particulate matter (PM10 and PM2.5), nitrogen dioxide (NO2), and ozone (O3) and all-cause and cause-specific mortality: Systematic review and meta-analysis. *Environ Int.* 2020; 142:105876.

71. Atkinson RW, Kang S, Anderson HR, Mills IC, Walton HA. Epidemiological time series studies of PM2.5 and daily mortality and hospital admissions: a systematic review and meta-analysis. *Thorax.* 2014; 69(7):660-665.

72. Yang H, Li S, Sun L, Zhang X, Cao Z, Xu C*, et al.* Smog and risk of overall and type-specific cardiovascular diseases: A pooled analysis of 53 cohort studies with 21.09 million participants. *Environ Res.* 2019; 172:375-383.

73. Liu Z, Wang F, Li W, Yin L, Wang Y, Yan R*, et al.* Does utilizing WHO's interim targets further reduce the risk - meta-analysis on ambient particulate matter pollution and mortality of cardiovascular diseases? *Environ Pollut.* 2018; 242(Pt B):1299-1307.

74. Atkinson RW, Butland BK, Anderson HR, Maynard RL. Long-term Concentrations of Nitrogen Dioxide and Mortality: A Meta-analysis of Cohort Studies. *Epidemiology.* 2018; 29(4):460-472.

75. Glinianaia SV, Rankin J, Bell R, Pless-Mulloli T, Howel D. Particulate air pollution and fetal health: a systematic review of the epidemiologic evidence. *Epidemiology.* 2004; 15(1):36-45.

76. Atkinson RW, Butland BK, Dimitroulopoulou C, Heal MR, Stedman JR, Carslaw N*, et al.* Long-term exposure to ambient ozone and mortality: a quantitative systematic review and meta-analysis of evidence from cohort studies. *BMJ Open.* 2016; 6(2):e009493.

77. Faustini A, Rapp R, Forastiere F. Nitrogen dioxide and mortality: review and meta-analysis of long-term studies. *Eur Respir J.* 2014; 44(3):744-753.

78. Stieb DM, Judek S, Burnett RT. Meta-analysis of time-series studies of air pollution and mortality: effects of gases and particles and the influence of cause of death, age, and season. *J Air Waste Manag Assoc.* 2002; 52(4):470-484.

79. Zhang Z, Yan W, Chen Q, Zhou N, Xu Y. The relationship between exposure to particulate matter and breast cancer incidence and mortality: A meta-analysis. *Medicine (Baltimore).* 2019; 98(50):e18349.

80. Ab Manan N, Noor Aizuddin A, Hod R. Effect of Air Pollution and Hospital Admission: A Systematic Review. *Ann Glob Health.* 2018; 84(4):670-678.

81. Guo Q, Wang X, Gao Y, Zhou J, Huang C, Zhang Z*, et al.* Relationship between particulate matter exposure and female breast cancer incidence and mortality: a systematic review and meta-analysis. *Int Arch Occup Environ Health.* 2021; 94(2):191-201.

82. Luben TJ, Nichols JL, Dutton SJ, Kirrane E, Owens EO, Datko-Williams L*, et al.* A systematic review of cardiovascular emergency department visits, hospital admissions and mortality associated with ambient black carbon. *Environ Int.* 2017; 107:154-162.

83. Koranteng S, Osornio Vargas A, Buka I. Ambient air pollution and children’s health: A systematic review of Canadian epidemiological studies. *Paediatr Child Health.* 2017; 12(3):225- 233.

84. Yang Y, Ruan Z, Wang X, Yang Y, Mason TG, Lin H*, et al.* Short-term and long-term exposures to fine particulate matter constituents and health: A systematic review and meta-analysis. *Environ Pollut.* 2019; 247:874-882.

85. Jacobs M, Zhang G, Chen S, Mullins B, Bell M, Jin L*, et al.* The association between ambient air pollution and selected adverse pregnancy outcomes in China: A systematic review. *Sci Total Environ.* 2017; 579:1179-1192.

86. Jaganathan S, Jaacks LM, Magsumbol M, Walia GK, Sieber NL, Shivasankar R*, et al.* Association of Long-Term Exposure to Fine Particulate Matter and Cardio-Metabolic Diseases in Low- and Middle-Income Countries: A Systematic Review. *Int J Environ Res Public Health.* 2019; 16(14).

87. Wu R, Song X, Bai Y, Chen J, Zhao Q, Liu S*, et al.* Are current Chinese national ambient air quality standards on 24-hour averages for particulate matter sufficient to protect public health? *J Environ Sci (China).* 2018; 71:67-75.

88. Moore E, Chatzidiakou L, Kuku MO, Jones RL, Smeeth L, Beevers S*, et al.* Global Associations between Air Pollutants and Chronic Obstructive Pulmonary Disease Hospitalizations. A Systematic Review. *Ann Am Thorac Soc.* 2016; 13(10):1814-1827.

89. Chen H, Goldberg MS, Villeneuve PJ. A Systematic Review of the Relation Between Long-Term Exposure to Ambient Air Pollution and Chronic Diseases. *REVIEWS ON ENVIRONMENTAL HEALTH.* 2008; 23(4):243-297.

90. King C, Kirkham J, Hawcutt D, Sinha I. The effect of outdoor air pollution on the risk of hospitalisation for bronchiolitis in infants: a systematic review. *PeerJ.* 2018; 6:e5352.

91. Bazyar J, Pourvakhshoori N, Khankeh H, Farrokhi M, Delshad V, Rajabi E. A comprehensive evaluation of the association between ambient air pollution and adverse health outcomes of major organ systems: a systematic review with a worldwide approach. *Environ Sci Pollut Res Int.* 2019; 26(13):12648-12661.

92. Yamamoto SS, Phalkey R, Malik AA. A systematic review of air pollution as a risk factor for cardiovascular disease in South Asia: limited evidence from India and Pakistan. *Int J Hyg Environ Health.* 2014; 217(2-3):133-144.

93. Levy JI, Chemerynski SM, Sarnat JA. Ozone exposure and mortality: an empiric bayes metaregression analysis. *Epidemiology.* 2005; 16(4):458-468.

94. Copat C, Cristaldi A, Fiore M, Grasso A, Zuccarello P, Signorelli SS*, et al.* The role of air pollution (PM and NO2) in COVID-19 spread and lethality: A systematic review. *Environ Res.* 2020; 191:110129.

95. Rajak R, Chattopadhyay A. Short and Long Term Exposure to Ambient Air Pollution and Impact on Health in India: A Systematic Review. *Int J Environ Health Res.* 2020; 30(6):593-617.

96. Teng TH, Williams TA, Bremner A, Tohira H, Franklin P, Tonkin A*, et al.* A systematic review of air pollution and incidence of out-of-hospital cardiac arrest. *J Epidemiol Community Health.* 2014; 68(1):37-43.

97. Jilani MH, Simon-Friedt B, Yahya T, Khan AY, Hassan SZ, Kash B*, et al.* Associations between particulate matter air pollution, presence and progression of subclinical coronary and carotid atherosclerosis: A systematic review. *Atherosclerosis.* 2020; 306:22-32.

98. Volk HE, Perera F, Braun JM, Kingsley SL, Gray K, Buckley J*, et al.* Prenatal air pollution exposure and neurodevelopment: A review and blueprint for a harmonized approach within ECHO. *Environ Res.* 2021; 196:110320.

99. Abdo N, Khader YS, Abdelrahman M, Graboski-Bauer A, Malkawi M, Al-Sharif M*, et al.* Respiratory health outcomes and air pollution in the Eastern Mediterranean Region: a systematic review. *Rev Environ Health.* 2016; 31(2):259-280.

100. Popovic I, Soares Magalhaes RJ, Ge E, Marks GB, Dong GH, Wei X*, et al.* A systematic literature review and critical appraisal of epidemiological studies on outdoor air pollution and tuberculosis outcomes. *Environ Res.* 2019; 170:33-45.

101. Buoli M, Grassi S, Caldiroli A, Carnevali GS, Mucci F, Iodice S*, et al.* Is there a link between air pollution and mental disorders? *Environ Int.* 2018; 118:154-168.

102. Li X, Chen Q, Zheng X, Li Y, Han M, Liu T*, et al.* Effects of ambient ozone concentrations with different averaging times on asthma exacerbations: A meta-analysis. *Sci Total Environ.* 2019; 691:549-561.

103. Froes Asmus CI, Camara VM, Landrigan PJ, Claudio L. A Systematic Review of Children's Environmental Health in Brazil. *Ann Glob Health.* 2016; 82(1):132-148.

104. Liu Y, Yan S, Poh K, Liu S, Iyioriobhe E, Sterling DA. Impact of air quality guidelines on COPD sufferers. *Int J Chron Obstruct Pulmon Dis.* 2016; 11:839-872.

105. Latza U, Gerdes S, Baur X. Effects of nitrogen dioxide on human health: systematic review of experimental and epidemiological studies conducted between 2002 and 2006. *Int J Hyg Environ Health.* 2009; 212(3):271-287.

106. Porpora MG, Piacenti I, Scaramuzzino S, Masciullo L, Rech F, Benedetti Panici P. Environmental Contaminants Exposure and Preterm Birth: A Systematic Review. *Toxics.* 2019; 7(1).

107. Wyzga RE, Rohr AC. Long-term particulate matter exposure: Attributing health effects to individual PM components. *J Air Waste Manag Assoc.* 2015; 65(5):523-543.

108. Wang N, Mengersen K, Kimlin M, Zhou M, Tong S, Fang L*, et al.* Lung cancer and particulate pollution: A critical review of spatial and temporal analysis evidence. *Environ Res.* 2018; 164:585-596.

109. Nasser Z, Salameh P, Nasser W, Abou Abbas L, Elias E, Leveque A. Outdoor particulate matter (PM) and associated cardiovascular diseases in the Middle East. *Int J Occup Med Environ Health.* 2015; 28(4):641-661.

110. Luben TJ, Buckley BJ, Patel MM, Stevens T, Coffman E, Rappazzo KM*, et al.* A cross-disciplinary evaluation of evidence for multipollutant effects on cardiovascular disease. *Environ Res.* 2018; 161:144-152.

111. Pelucchi C, Negri E, Gallus S, Boffetta P, Tramacere I, La Vecchia C. Long-term particulate matter exposure and mortality: a review of European epidemiological studies. *BMC Public Health.* 2009; 9:453.

112. Maitre A, Bonneterre V, Huillard L, Sabatier P, de Gaudemaris R. Impact of urban atmospheric pollution on coronary disease. *Eur Heart J.* 2006; 27(19):2275-2284.

113. Mustafic H, Jabre P, Caussin C, Murad MH, Escolano S, Tafflet M*, et al.* Main air pollutants and myocardial infarction: a systematic review and meta-analysis. *JAMA.* 2012; 307(7):713-721.

114. Mehta S, Shin H, Burnett R, North T, Cohen AJ. Ambient particulate air pollution and acute lower respiratory infections: a systematic review and implications for estimating the global burden of disease. *Air Qual Atmos Health.* 2013; 6(1):69-83.

115. Wang B, Xu D, Jing Z, Liu D, Yan S, Wang Y. Effect of long-term exposure to air pollution on type 2 diabetes mellitus risk: a systemic review and meta-analysis of cohort studies. *Eur J Endocrinol.* 2014; 171(5):R173-182.

116. Ji M, Cohan DS, Bell ML. Meta-analysis of the Association between Short-Term Exposure to Ambient Ozone and Respiratory Hospital Admissions. *Environ Res Lett.* 2011; 6(2).

117. Li J, Sun S, Tang R, Qiu H, Huang Q, Mason TG*, et al.* Major air pollutants and risk of COPD exacerbations: a systematic review and meta-analysis. *Int J Chron Obstruct Pulmon Dis.* 2016; 11:3079-3091.

118. Morris RD. Airborne particulates and hospital admissions for cardiovascular disease: a quantitative review of the evidence. *Environmental Health Perspectives.* 2001; 109(suppl 4):495-500.

119. Janghorbani M, Momeni F, Mansourian M. Systematic review and metaanalysis of air pollution exposure and risk of diabetes. *Eur J Epidemiol.* 2014; 29(4):231-242.

120. Atkinson RW, Cohen A, Mehta S, Anderson HR. Systematic review and meta-analysis of epidemiological time-series studies on outdoor air pollution and health in Asia. *Air Quality, Atmosphere & Health.* 2011; 5(4):383-391.

121. Zhang X, Zhao L, Tong D, Wu G, Dan M, Teng B. A Systematic Review of Global Desert Dust and Associated Human Health Effects. *Atmosphere.* 2016; 7(12).

122. Luo C, Zhu X, Yao C, Hou L, Zhang J, Cao J*, et al.* Short-term exposure to particulate air pollution and risk of myocardial infarction: a systematic review and meta-analysis. *Environ Sci Pollut Res Int.* 2015; 22(19):14651-14662.

123. Peters R, Ee N, Peters J, Booth A, Mudway I, Anstey KJ. Air Pollution and Dementia: A Systematic Review. *J Alzheimers Dis.* 2019; 70(s1):S145-S163.

124. Franchini M, Mengoli C, Cruciani M, Bonfanti C, Mannucci PM. Association between particulate air pollution and venous thromboembolism: A systematic literature review. *Eur J Intern Med.* 2016; 27:10-13.

125. Akintoye E, Shi L, Obaitan I, Olusunmade M, Wang Y, Newman JD*, et al.* Association between fine particulate matter exposure and subclinical atherosclerosis: A meta-analysis. *Eur J Prev Cardiol.* 2016; 23(6):602-612.

126. Li XY, Yu XB, Liang WW, Yu N, Wang L, Ye XJ*, et al.* Meta-analysis of association between particulate matter and stroke attack. *CNS Neurosci Ther.* 2012; 18(6):501-508.

127. Tsai TL, Lin YT, Hwang BF, Nakayama SF, Tsai CH, Sun XL*, et al.* Fine particulate matter is a potential determinant of Alzheimer's disease: A systemic review and meta-analysis. *Environ Res.* 2019; 177:108638.

128. Spiric VT, Jankovic S, Vranes AJ, Maksimovic J, Maksimovic N. The Impact of Air Pollution on Chronic Respiratory Diseases. *Polish Journal of Environmental Studies.* 2012; 21(2):481-490.

129. Luong LMT, Sly PD, Thai PK, Phung D. Impact of ambient air pollution and wheeze-associated disorders in children in Southeast Asia: a systematic review and meta-analysis. *Rev Environ Health.* 2019; 34(2):125-139.

130. Kim HB, Shim JY, Park B, Lee YJ. Long-term exposure to air pollution and the risk of non-lung cancer: a meta-analysis of observational studies. *Perspect Public Health.* 2020; 140(4):222-231.

131. Sun M, Yan W, Fang K, Chen D, Liu J, Chen Y*, et al.* The correlation between PM2.5 exposure and hypertensive disorders in pregnancy: A Meta-analysis. *Sci Total Environ.* 2020; 703:134985.

132. Chen R, Yang J, Zhang C, Li B, Bergmann S, Zeng F*, et al.* Global Associations of Air Pollution and Conjunctivitis Diseases: A Systematic Review and Meta-Analysis. *Int J Environ Res Public Health.* 2019; 16(19).

133. Sharma S, Chandra M, Kota SH. Health Effects Associated with PM2.5: a Systematic Review. *Current Pollution Reports.* 2020; 6(4):345-367.

134. Vieira SE. The health burden of pollution: the impact of prenatal exposure to air pollutants. *Int J Chron Obstruct Pulmon Dis.* 2015; 10:1111-1121.

135. Bell ML, Dominici F, Samet JM. A meta-analysis of time-series studies of ozone and mortality with comparison to the national morbidity, mortality, and air pollution study. *Epidemiology.* 2005; 16(4):436-445.

136. Kan H, Chen B, Chen C, Wang B, Fu Q. Establishment of Exposure-response Functions of Air Particulate Matter and Adverse Health Outcomes in China and Worldwide. *Biomedical and Environmental Sciences.* 2005; 18(3):159-163.

137. Conforti A, Mascia M, Cioffi G, De Angelis C, Coppola G, De Rosa P*, et al.* Air pollution and female fertility: a systematic review of literature. *Reprod Biol Endocrinol.* 2018; 16(1):117.

138. Keramatinia A, Hassanipour S, Nazarzadeh M, Wurtz M, Monfared AB, Khayyamzadeh M*, et al.* Correlation Between Nitrogen Dioxide as an Air Pollution Indicator and Breast Cancer: a Systematic Review and Meta- Analysis. *Asian Pac J Cancer Prev.* 2016; 17(1):419-424.

139. Cheng J, Xu Z, Bambrick H, Prescott V, Wang N, Zhang Y*, et al.* Cardiorespiratory effects of heatwaves: A systematic review and meta-analysis of global epidemiological evidence. *Environ Res.* 2019; 177:108610.

140. Green H, Bailey J, Schwarz L, Vanos J, Ebi K, Benmarhnia T. Impact of heat on mortality and morbidity in low and middle income countries: A review of the epidemiological evidence and considerations for future research. *Environ Res.* 2019; 171:80-91.

141. van Steen Y, Ntarladima AM, Grobbee R, Karssenberg D, Vaartjes I. Sex differences in mortality after heat waves: are elderly women at higher risk? *Int Arch Occup Environ Health.* 2019; 92(1):37-48.

142. Sun Z, Chen C, Xu D, Li T. Effects of ambient temperature on myocardial infarction: A systematic review and meta-analysis. *Environ Pollut.* 2018; 241:1106-1114.

143. Campbell S, Remenyi TA, White CJ, Johnston FH. Heatwave and health impact research: A global review. *Health Place.* 2018; 53:210-218.

144. Odame EA, Li Y, Zheng S, Vaidyanathan A, Silver K. Assessing Heat-Related Mortality Risks among Rural Populations: A Systematic Review and Meta-Analysis of Epidemiological Evidence. *Int J Environ Res Public Health.* 2018; 15(8).

145. Ghanizadeh G, Heidari M, Seifi B, Jafari H, Pakjouei S. The Effect of Climate Change on Cardiopulmonary Disease - A Systematic Review. *Journal of Clinical and Diagnostic Research.* 2017.

146. Kuehn L, McCormick S. Heat Exposure and Maternal Health in the Face of Climate Change. *Int J Environ Res Public Health.* 2017; 14(8).

147. Zhang Y, Yu C, Wang L. Temperature exposure during pregnancy and birth outcomes: An updated systematic review of epidemiological evidence. *Environ Pollut.* 2017; 225:700-712.

148. Philipsborn R, Ahmed SM, Brosi BJ, Levy K. Climatic Drivers of Diarrheagenic Escherichia coli Incidence: A Systematic Review and Meta-analysis. *J Infect Dis.* 2016; 214(1):6-15.

149. Asadgol Z, Badirzadeh A, Niazi S, Mokhayeri Y, Kermani M, Mohammadi H*, et al.* How climate change can affect cholera incidence and prevalence? A systematic review. *Environ Sci Pollut Res Int.* 2020; 27(28):34906-34926.

150. Bai XH, Peng C, Jiang T, Hu ZM, Huang DS, Guan P. Distribution of geographical scale, data aggregation unit and period in the correlation analysis between temperature and incidence of HFRS in mainland China: A systematic review of 27 ecological studies. *PLoS Negl Trop Dis.* 2019; 13(8):e0007688.

151. Lu C, Yu Y, Li L, Yu C, Xu P. Systematic review of the relationship of Helicobacter pylori infection with geographical latitude, average annual temperature and average daily sunshine. *BMC Gastroenterol.* 2018; 18(1):50.

152. Coates SJ, Davis MDP, Andersen LK. Temperature and humidity affect the incidence of hand, foot, and mouth disease: a systematic review of the literature - a report from the International Society of Dermatology Climate Change Committee. *Int J Dermatol.* 2019; 58(4):388-399.

153. Shi T, Min M, Ye P, Wang Y, Qu G, Zhang Y*, et al.* Meteorological variables and the risk of fractures: A systematic review and meta-analysis. *Sci Total Environ.* 2019; 685:1030-1041.

154. Son J-Y, Liu JC, Bell ML. Temperature-related mortality: a systematic review and investigation of effect modifiers. *Environmental Research Letters.* 2019; 14(7).

155. Gao J, Cheng Q, Duan J, Xu Z, Bai L, Zhang Y*, et al.* Ambient temperature, sunlight duration, and suicide: A systematic review and meta-analysis. *Sci Total Environ.* 2019; 646:1021-1029.

156. Salve HR, Parthasarathy R, Krishnan A, Pattanaik DR. Impact of ambient air temperature on human health in India. *Rev Environ Health.* 2018; 33(4):433-439.

157. Chersich MF, Wright CY, Venter F, Rees H, Scorgie F, Erasmus B. Impacts of Climate Change on Health and Wellbeing in South Africa. *Int J Environ Res Public Health.* 2018; 15(9).

158. Cheng Q, Bai L, Zhang Y, Zhang H, Wang S, Xie M*, et al.* Ambient temperature, humidity and hand, foot, and mouth disease: A systematic review and meta-analysis. *Sci Total Environ.* 2018; 625:828-836.

159. Ghazani M, FitzGerald G, Hu W, Toloo GS, Xu Z. Temperature Variability and Gastrointestinal Infections: A Review of Impacts and Future Perspectives. *Int J Environ Res Public Health.* 2018; 15(4).

160. Chan EYY, Ho JY, Hung HHY, Liu S, Lam HCY. Health impact of climate change in cities of middle-income countries: the case of China. *Br Med Bull.* 2019; 130(1):5-24.

161. Zanobetti A, O'Neill MS. Longer-Term Outdoor Temperatures and Health Effects: A Review. *Curr Epidemiol Rep.* 2018; 5(2):125-139.

162. Ma Y, Zhang Y, Cheng B, Feng F, Jiao H, Zhao X*, et al.* A review of the impact of outdoor and indoor environmental factors on human health in China. *Environ Sci Pollut Res Int.* 2020; 27(34):42335-42345.

163. Chersich MF, Pham MD, Areal A, Haghighi MM, Manyuchi A, Swift CP*, et al.* Associations between high temperatures in pregnancy and risk of preterm birth, low birth weight, and stillbirths: systematic review and meta-analysis. *BMJ.* 2020; 371:m3811.

164. Chang X, Zhou L, Tang M, Wang B. Association of Fine Particles With Respiratory Disease Mortality: A Meta-Analysis. *Archives of Environmental & Occupational Health.* 2015; 70(2):98-101.

165. Cheng J, Xu Z, Bambrick H, Su H, Tong S, Hu W. Impacts of exposure to ambient temperature on burden of disease: a systematic review of epidemiological evidence. *Int J Biometeorol.* 2019; 63(8):1099-1115.

166. Luo Q, Li S, Guo Y, Han X, Jaakkola JJK. A systematic review and meta-analysis of the association between daily mean temperature and mortality in China. *Environ Res.* 2019; 173:281-299.

167. Bodaghkhani E, Mahdavian M, MacLellan C, Farrell A, Asghari S. Effects of Meteorological Factors on Hospitalizations in Adult Patients with Asthma: A Systematic Review. *Can Respir J.* 2019; 2019:3435103.

168. Thompson R, Hornigold R, Page L, Waite T. Associations between high ambient temperatures and heat waves with mental health outcomes: a systematic review. *Public Health.* 2018; 161:171-191.

169. Schinasi LH, Benmarhnia T, De Roos AJ. Modification of the association between high ambient temperature and health by urban microclimate indicators: A systematic review and meta-analysis. *Environ Res.* 2018; 161:168-180.

170. Leyva EWA, Beaman A, Davidson PM. Health Impact of Climate Change in Older People: An Integrative Review and Implications for Nursing. *J Nurs Scholarsh.* 2017; 49(6):670-678.

171. Geraghty RM, Proietti S, Traxer O, Archer M, Somani BK. Worldwide Impact of Warmer Seasons on the Incidence of Renal Colic and Kidney Stone Disease: Evidence from a Systematic Review of Literature. *J Endourol.* 2017; 31(8):729-735.

172. Moghadamnia MT, Ardalan A, Mesdaghinia A, Keshtkar A, Naddafi K, Yekaninejad MS. Ambient temperature and cardiovascular mortality: a systematic review and meta-analysis. *PeerJ.* 2017; 5:e3574.

173. Mousavi A, Ardalan A, Takian A, Ostadtaghizadeh A, Naddafi K, Bavani AM. Climate change and health in Iran: a narrative review. *J Environ Health Sci Eng.* 2020; 18(1):367-378.

174. Arbuthnott K, Hajat S, Heaviside C, Vardoulakis S. Changes in population susceptibility to heat and cold over time: assessing adaptation to climate change. *Environ Health.* 2016; 15 Suppl 1:33.

175. Heidari H, Mohammadbeigi A, Khazaei S, Soltanzadeh A, Asgarian A, Saghafipour A. The effects of climatic and environmental factors on heat-related illnesses: A systematic review from 2000 to 2020. *Urban Climate.* 2020; 34.

176. Anita AR, Tan HS, Fatimah AF, E. N, Juni MH. PUBLIC HEALTH IMPACTS OF HEAT WAVES: A REVIEW. *International Journal of Public Health and Clinical Sciences.* 2018; 5(2):68-85.

177. Otte im Kampe E, Kovats S, Hajat S. Impact of high ambient temperature on unintentional injuries in high-income countries: a narrative systematic literature review. *BMJ Open.* 2016; 6(2):e010399.

178. Amegah AK, Rezza G, Jaakkola JJ. Temperature-related morbidity and mortality in Sub-Saharan Africa: A systematic review of the empirical evidence. *Environ Int.* 2016; 91:133-149.

179. Astrom DO, Forsberg B, Rocklov J. Heat wave impact on morbidity and mortality in the elderly population: a review of recent studies. *Maturitas.* 2011; 69(2):99-105.

180. Basu R. High ambient temperature and mortality: a review of epidemiologic studies from 2001 to 2008. *Environ Health.* 2009; 8:40.

181. Martiello MA, Giacchi MV. High temperatures and health outcomes: a review of the literature. *Scand J Public Health.* 2010; 38(8):826-837.

182. Witt C, Schubert AJ, Jehn M, Holzgreve A, Liebers U, Endlicher W*, et al.* The Effects of Climate Change on Patients With Chronic Lung Disease. A Systematic Literature Review. *Dtsch Arztebl Int.* 2015; 112(51-52):878-883.

183. Turner LR, Barnett AG, Connell D, Tong S. Ambient temperature and cardiorespiratory morbidity: a systematic review and meta-analysis. *Epidemiology.* 2012; 23(4):594-606.

184. Ye X, Wolff R, Yu W, Vaneckova P, Pan X, Tong S. Ambient temperature and morbidity: a review of epidemiological evidence. *Environ Health Perspect.* 2012; 120(1):19-28.

185. Ryti NR, Guo Y, Jaakkola JJ. Global Association of Cold Spells and Adverse Health Effects: A Systematic Review and Meta-Analysis. *Environ Health Perspect.* 2016; 124(1):12-22.

186. Basu R, Samet JM. Relation between elevated ambient temperature and mortality: a review of the epidemiologic evidence. *Epidemiol Rev.* 2002; 24(2):190-202.

187. Bhaskaran K, Hajat S, Haines A, Herrett E, Wilkinson P, Smeeth L. Effects of ambient temperature on the incidence of myocardial infarction. *Heart.* 2009; 95(21):1760-1769.

188. Carlton EJ, Woster AP, DeWitt P, Goldstein RS, Levy K. A systematic review and meta-analysis of ambient temperature and diarrhoeal diseases. *Int J Epidemiol.* 2016; 45(1):117-130.

189. Beltran AJ, Wu J, Laurent O. Associations of meteorology with adverse pregnancy outcomes: a systematic review of preeclampsia, preterm birth and birth weight. *Int J Environ Res Public Health.* 2013; 11(1):91-172.

190. Fan J, Wei W, Bai Z, Fan C, Li S, Liu Q*, et al.* A systematic review and meta-analysis of dengue risk with temperature change. *Int J Environ Res Public Health.* 2014; 12(1):1-15.

191. Li M, Gu S, Bi P, Yang J, Liu Q. Heat waves and morbidity: current knowledge and further direction-a comprehensive literature review. *Int J Environ Res Public Health.* 2015; 12(5):5256-5283.

192. Lian H, Ruan Y, Liang R, Liu X, Fan Z. Short-Term Effect of Ambient Temperature and the Risk of Stroke: A Systematic Review and Meta-Analysis. *Int J Environ Res Public Health.* 2015; 12(8):9068-9088.

193. Bunker A, Wildenhain J, Vandenbergh A, Henschke N, Rocklov J, Hajat S*, et al.* Effects of Air Temperature on Climate-Sensitive Mortality and Morbidity Outcomes in the Elderly; a Systematic Review and Meta-analysis of Epidemiological Evidence. *EBioMedicine.* 2016; 6:258-268.

194. Xu Z, FitzGerald G, Guo Y, Jalaludin B, Tong S. Impact of heatwave on mortality under different heatwave definitions: A systematic review and meta-analysis. *Environ Int.* 2016; 89-90:193-203.

195. Strand LB, Barnett AG, Tong S. The influence of season and ambient temperature on birth outcomes: a review of the epidemiological literature. *Environ Res.* 2011; 111(3):451-462.

196. Xu Z, Etzel RA, Su H, Huang C, Guo Y, Tong S. Impact of ambient temperature on children's health: a systematic review. *Environ Res.* 2012; 117:120-131.

197. Carolan-Olah M, Frankowska D. High environmental temperature and preterm birth: a review of the evidence. *Midwifery.* 2014; 30(1):50-59.

198. Phung D, Thai PK, Guo Y, Morawska L, Rutherford S, Chu C. Ambient temperature and risk of cardiovascular hospitalization: An updated systematic review and meta-analysis. *Sci Total Environ.* 2016; 550:1084-1102.

199. Ramesh A, Kovats S, Haslam D, Schmidt E, Gilbert CE. The impact of climatic risk factors on the prevalence, distribution, and severity of acute and chronic trachoma. *PLoS Negl Trop Dis.* 2013; 7(11):e2513.

200. Yu W, Mengersen K, Wang X, Ye X, Guo Y, Pan X*, et al.* Daily average temperature and mortality among the elderly: a meta-analysis and systematic review of epidemiological evidence. *Int J Biometeorol.* 2012; 56(4):569-581.

201. Xu Z, Sheffield PE, Su H, Wang X, Bi Y, Tong S. The impact of heat waves on children's health: a systematic review. *Int J Biometeorol.* 2014; 58(2):239-247.

202. Cheng J, Xu Z, Zhu R, Wang X, Jin L, Song J*, et al.* Impact of diurnal temperature range on human health: a systematic review. *Int J Biometeorol.* 2014; 58(9):2011-2024.

203. VianaI DV, Ignotti E. The ocurrence of dengue and weather changes in Brazil: A systematic review. *Rev Bras Epidemiol.* 2013; 16(2):240-256.

204. Burkart K, Khan MM, Schneider A, Breitner S, Langner M, Kramer A*, et al.* The effects of season and meteorology on human mortality in tropical climates: a systematic review. *Trans R Soc Trop Med Hyg.* 2014; 108(7):393-401.

205. Poursafa P, Keikha M, Kelishadi R. Systematic review on adverse birth outcomes of climate change. *Journal of Research in Medical Sciences.* 2015; 20(4):397-402.

206. Anenberg SC, Haines S, Wang E, Nassikas N, Kinney PL. Synergistic health effects of air pollution, temperature, and pollen exposure: a systematic review of epidemiological evidence. *Environ Health.* 2020; 19(1):130.

207. Chen F, Fan Z, Qiao Z, Cui Y, Zhang M, Zhao X*, et al.* Does temperature modify the effect of PM10 on mortality? A systematic review and meta-analysis. *Environ Pollut.* 2017; 224:326-335.

208. Li J, Woodward A, Hou XY, Zhu T, Zhang J, Brown H*, et al.* Modification of the effects of air pollutants on mortality by temperature: A systematic review and meta-analysis. *Sci Total Environ.* 2017; 575:1556-1570.

209. Zang ST, Luan J, Li L, Yu HX, Wu QJ, Chang Q*, et al.* Ambient air pollution and COVID-19 risk: Evidence from 35 observational studies. *Environ Res.* 2022; 204(Pt B):112065.

210. Yee J, Cho YA, Yoo HJ, Yun H, Gwak HS. Short-term exposure to air pollution and hospital admission for pneumonia: a systematic review and meta-analysis. *Environ Health.* 2021; 20(1):6.

211. Niu Z, Liu F, Yu H, Wu S, Xiang H. Association between exposure to ambient air pollution and hospital admission, incidence, and mortality of stroke: an updated systematic review and meta-analysis of more than 23 million participants. *Environ Health Prev Med.* 2021; 26(1):15.

212. Katoto P, Brand AS, Bakan B, Obadia PM, Kuhangana C, Kayembe-Kitenge T*, et al.* Acute and chronic exposure to air pollution in relation with incidence, prevalence, severity and mortality of COVID-19: a rapid systematic review. *Environ Health.* 2021; 20(1):41.

213. Hu Y, Wu M, Li Y, Liu X. Influence of PM1 exposure on total and cause-specific respiratory diseases: a systematic review and meta-analysis. *Environ Sci Pollut Res Int.* 2022; 29(10):15117-15126.

214. Zheng J, Yang X, Hu S, Wang Y, Liu J. Association between short-term exposure to air pollution and respiratory diseases among children in China: a systematic review and meta-analysis. *Int J Environ Health Res.* 2021:1-21.

215. Liu JJ, Fu SB, Jiang J, Tang XL. Association between outdoor particulate air pollution and the risk of osteoporosis: a systematic review and meta-analysis. *Osteoporos Int.* 2021; 32(10):1911-1919.

216. Walter CM, Schneider-Futschik EK, Lansbury NL, Sly PD, Head BW, Knibbs LD. The health impacts of ambient air pollution in Australia: a systematic literature review. *Intern Med J.* 2021; 51(10):1567-1579.

217. Yu G, Chen Y, Tang J, Lin Z, Zheng F, Zheng C*, et al.* Meta-analyses of maternal exposure to atmospheric particulate matter and risk of congenital anomalies in offspring. *Environ Sci Pollut Res Int.* 2021; 28(40):55869-55887.

218. Huang S, Li H, Wang M, Qian Y, Steenland K, Caudle WM*, et al.* Long-term exposure to nitrogen dioxide and mortality: A systematic review and meta-analysis. *Sci Total Environ.* 2021; 776:145968.

219. Davoudi M, Barjasteh-Askari F, Amini H, Lester D, Mahvi AH, Ghavami V*, et al.* Association of suicide with short-term exposure to air pollution at different lag times: A systematic review and meta-analysis. *Sci Total Environ.* 2021; 771:144882.

220. Zhang H, Zhang X, Wang Q, Xu Y, Feng Y, Yu Z*, et al.* Ambient air pollution and stillbirth: An updated systematic review and meta-analysis of epidemiological studies. *Environ Pollut.* 2021; 278:116752.

221. Ciabattini M, Rizzello E, Lucaroni F, Palombi L, Boffetta P. Systematic review and meta-analysis of recent high-quality studies on exposure to particulate matter and risk of lung cancer. *Environ Res.* 2021; 196:110440.

222. Orellano P, Reynoso J, Quaranta N. Short-term exposure to sulphur dioxide (SO2) and all-cause and respiratory mortality: A systematic review and meta-analysis. *Environ Int.* 2021; 150:106434.

223. Zheng XY, Orellano P, Lin HL, Jiang M, Guan WJ. Short-term exposure to ozone, nitrogen dioxide, and sulphur dioxide and emergency department visits and hospital admissions due to asthma: A systematic review and meta-analysis. *Environ Int.* 2021; 150:106435.

224. Maleki M, Anvari E, Hopke PK, Noorimotlagh Z, Mirzaee SA. An updated systematic review on the association between atmospheric particulate matter pollution and prevalence of SARS-CoV-2. *Environ Res.* 2021; 195:110898.

225. Zhu W, Cai J, Hu Y, Zhang H, Han X, Zheng H*, et al.* Long-term exposure to fine particulate matter relates with incident myocardial infarction (MI) risks and post-MI mortality: A meta-analysis. *Chemosphere.* 2021; 267:128903.

226. Park J, Kim HJ, Lee CH, Lee CH, Lee HW. Impact of long-term exposure to ambient air pollution on the incidence of chronic obstructive pulmonary disease: A systematic review and meta-analysis. *Environ Res.* 2021; 194:110703.

227. Stieb DM, Berjawi R, Emode M, Zheng C, Salama D, Hocking R*, et al.* Systematic review and meta-analysis of cohort studies of long term outdoor nitrogen dioxide exposure and mortality. *PLoS One.* 2021; 16(2):e0246451.

228. Alexeeff SE, Liao NS, Liu X, Van Den Eeden SK, Sidney S. Long-Term PM2.5 Exposure and Risks of Ischemic Heart Disease and Stroke Events: Review and Meta-Analysis. *J Am Heart Assoc.* 2021; 10(1):e016890.

229. Prueitt RL, Li W, Edwards L, Zhou J, Goodman JE. Systematic review of the association between long-term exposure to fine particulate matter and mortality. *Int J Environ Health Res.* 2022; 32(8):1647-1685.

230. S.A. MEO1 TA-K, C.H. ULLAH2. Effect of ambient air pollutants PM2.5 and PM10 on COVID-19 incidence and mortality: observational study. *European Review for Medical and Pharmacological Sciences.* 2021; 25(23):7553-7564.

231. Zhang Y, Ma Y, Feng F, Cheng B, Shen J, Wang H*, et al.* Respiratory mortality associated with ozone in China: A systematic review and meta-analysis. *Environ Pollut.* 2021; 280:116957.

232. Ning J, Zhang Y, Hu H, Hu W, Li L, Pang Y*, et al.* Association between ambient particulate matter exposure and metabolic syndrome risk: A systematic review and meta-analysis. *Sci Total Environ.* 2021; 782:146855.

233. Xiang K, Xu Z, Hu YQ, He YS, Dan YL, Wu Q*, et al.* Association between ambient air pollution and tuberculosis risk: A systematic review and meta-analysis. *Chemosphere.* 2021; 277:130342.

234. Zou L, Zong Q, Fu W, Zhang Z, Xu H, Yan S*, et al.* Long-Term Exposure to Ambient Air Pollution and Myocardial Infarction: A Systematic Review and Meta-Analysis. *Front Med (Lausanne).* 2021; 8:616355.

235. Trushna T, Dhiman V, Raj D, Tiwari RR. Effects of ambient air pollution on psychological stress and anxiety disorder: a systematic review and meta-analysis of epidemiological evidence. *Rev Environ Health.* 2021; 36(4):501-521.

236. Chen M, Zhao J, Zhuo C, Zheng L. The Association Between Ambient Air Pollution and Atrial Fibrillation. *International Heart Journal.* 2021; 62(2):290-297.

237. Zhao K, Li J, Du C, Zhang Q, Guo Y, Yang M. Ambient fine particulate matter of diameter </= 2.5 mum and risk of hemorrhagic stroke: a systemic review and meta-analysis of cohort studies. *Environ Sci Pollut Res Int.* 2021; 28(17):20970-20980.

238. Ma Y, Sun M, Liang Q, Wang F, Lin L, Li T*, et al.* The relationship between long-term exposure to PM2.5 and hypertension in womenA meta-analysis. *Ecotoxicol Environ Saf.* 2021; 208:111492.

239. Yue C, Yang F, Li F, Chen Y. Association between air pollutants and atrial fibrillation in general population: A systematic review and meta-analysis. *Ecotoxicol Environ Saf.* 2021; 208:111508.

240. Noorimotlagh Z, Azizi M, Pan HF, Mami S, Mirzaee SA. Association between air pollution and Multiple Sclerosis: A systematic review. *Environ Res.* 2021; 196:110386.

241. Ibrahim MF, Hod R, Nawi AM, Sahani M. Association between ambient air pollution and childhood respiratory diseases in low- and middle-income Asian countries: A systematic review. *Atmospheric Environment.* 2021; 256:118422.

242. Lederer AM, Fredriksen PM, Nkeh-Chungag BN, Everson F, Strijdom H, De Boever P*, et al.* Cardiovascular effects of air pollution: current evidence from animal and human studies. *Am J Physiol Heart Circ Physiol.* 2021; 320(4):H1417-H1439.

243. Ghosh R, Causey K, Burkart K, Wozniak S, Cohen A, Brauer M. Ambient and household PM2.5 pollution and adverse perinatal outcomes: A meta-regression and analysis of attributable global burden for 204 countries and territories. *PLoS Med.* 2021; 18(9):e1003718.

244. Shahrbaf MA, Akbarzadeh MA, Tabary M, Khaheshi I. Air Pollution and Cardiac Arrhythmias: A Comprehensive Review. *Curr Probl Cardiol.* 2021; 46(3):100649.

245. Bernardini F, Trezzi R, Quartesan R, Attademo L. Air Pollutants and Daily Hospital Admissions for Psychiatric Care: A Review. *Psychiatr Serv.* 2020; 71(12):1270-1276.

246. Xie G, Sun L, Yang W, Wang R, Shang L, Yang L*, et al.* Maternal exposure to PM2.5 was linked to elevated risk of stillbirth. *Chemosphere.* 2021; 283:131169.

247. Lin L, Li T, Sun M, Liang Q, Ma Y, Wang F*, et al.* Effect of particulate matter exposure on the prevalence of allergic rhinitis in children: A systematic review and meta-analysis. *Chemosphere.* 2021; 268:128841.

248. Ma Z, Cao X, Chang Y, Li W, Chen X, Tang NJ. Association between gestational exposure and risk of congenital heart disease: A systematic review and meta-analysis. *Environ Res.* 2021; 197:111014.

249. Wang F, Ahat X, Liang Q, Ma Y, Sun M, Lin L*, et al.* The relationship between exposure to PM2.5 and atrial fibrillation in older adults: A systematic review and meta-analysis. *Sci Total Environ.* 2021; 784:147106.

250. Wang M, Li H, Huang S, Qian Y, Steenland K, Xie Y*, et al.* Short-term exposure to nitrogen dioxide and mortality: A systematic review and meta-analysis. *Environ Res.* 2021; 202:111766.

251. Yu P, Guo S, Xu R, Ye T, Li S, Sim MR*, et al.* Cohort studies of long-term exposure to outdoor particulate matter and risks of cancer: A systematic review and meta-analysis. *Innovation (Camb).* 2021; 2(3):100143.

252. Wu ZH, Zhao M, Yu H, Li HD. The impact of particulate matter 2.5 on the risk of hepatocellular carcinoma: a meta-analysis. *Int Arch Occup Environ Health.* 2022; 95(3):677-683.

253. Uwak I, Olson N, Fuentes A, Moriarty M, Pulczinski J, Lam J*, et al.* Application of the navigation guide systematic review methodology to evaluate prenatal exposure to particulate matter air pollution and infant birth weight. *Environ Int.* 2021; 148:106378.

254. Farhadi Z, Abulghasem Gorgi H, Shabaninejad H, Aghajani Delavar M, Torani S. Association between PM2.5 and risk of hospitalization for myocardial infarction: a systematic review and a meta-analysis. *BMC Public Health.* 2020; 20(1):314.

255. Sun J, Zhang N, Yan X, Wang M, Wang J. The effect of ambient fine particulate matter (PM2.5) on respiratory diseases in China: a systematic review and meta-analysis. *Stochastic Environmental Research and Risk Assessment.* 2020; 34(3-4):593-610.

256. Bai W, Li Y, Niu Y, Ding Y, Yu X, Zhu B*, et al.* Association between ambient air pollution and pregnancy complications: A systematic review and meta-analysis of cohort studies. *Environ Res.* 2020; 185:109471.

257. Kim H-J, Lee HW, Park J, Lee CH, Lee C-H. Short-term exposure to fine particulate matter and pneumonia-related hospitalizations: a systematic review and meta-analysis. *Environmental Research Letters.* 2020; 15(12):123012.

258. Harari S, Raghu G, Caminati A, Cruciani M, Franchini M, Mannucci P. Fibrotic interstitial lung diseases and air pollution: a systematic literature review. *Eur Respir Rev.* 2020; 29(157).

259. Villeneuve PJ, Goldberg MS. Methodological Considerations for Epidemiological Studies of Air Pollution and the SARS and COVID-19 Coronavirus Outbreaks. *Environ Health Perspect.* 2020; 128(9):95001.

260. Wang Y, Liu Y, Yan H. Effect of long-term particulate matter exposure on Parkinson's risk. *Environ Geochem Health.* 2020; 42(7):2265-2275.

261. Yang M, Cheng H, Shen C, Liu J, Zhang H, Cao J*, et al.* Effects of long-term exposure to air pollution on the incidence of type 2 diabetes mellitus: a meta-analysis of cohort studies. *Environ Sci Pollut Res Int.* 2020; 27(1):798-811.

262. Amiri M, Peinkhofer C, Othman MH, De Vecchi T, Nersesjan V, Kondziella D. Global warming and neurological practice: systematic review. *PeerJ.* 2021; 9:e11941.

263. Dimitrova A, Ingole V, Basagana X, Ranzani O, Mila C, Ballester J*, et al.* Association between ambient temperature and heat waves with mortality in South Asia: Systematic review and meta-analysis. *Environ Int.* 2021; 146:106170.

264. Kakaei S, Zakerimoghadam M, Rahmanian M, Abbasi Dolatabadi Z. The Impact of Climate Change on Heart Failure: A Narrative Review Study. *Shiraz E-Medical Journal.* 2021; 22(9):e107895.

265. Frangione B, Rodriguez Villamizar LA, Lang JJ, Colman I, Lavigne E, Peters C*, et al.* Short-term changes in meteorological conditions and suicide: A systematic review and meta-analysis. *Environ Res.* 2022; 207:112230.

266. Abed Al Ahad M, Sullivan F, Demsar U, Melhem M, Kulu H. The effect of air-pollution and weather exposure on mortality and hospital admission and implications for further research: A systematic scoping review. *PLoS One.* 2020; 15(10):e0241415.

267. Heo S, Lee W, Bell ML. Suicide and Associations with Air Pollution and Ambient Temperature: A Systematic Review and Meta-Analysis. *Int J Environ Res Public Health.* 2021; 18(14).

268. Liu J, Varghese BM, Hansen A, Borg MA, Zhang Y, Driscoll T*, et al.* Hot weather as a risk factor for kidney disease outcomes: A systematic review and meta-analysis of epidemiological evidence. *Sci Total Environ.* 2021; 801:149806.

269. Majumder P, Ray PP. A systematic review and meta‐analysis on correlation of weather with COVID‐19. 2021; 11(1).

270. Moon J. The effect of the heatwave on the morbidity and mortality of diabetes patients; a meta-analysis for the era of the climate crisis. *Environ Res.* 2021; 195:110762.

271. Romero Starke K, Mauer R, Karskens E, Pretzsch A, Reissig D, Nienhaus A*, et al.* The Effect of Ambient Environmental Conditions on COVID-19 Mortality: A Systematic Review. *Int J Environ Res Public Health.* 2021; 18(12).

272. Sexton J, Andrews C, Carruthers S, Kumar S, Flenady V, Lieske S. Systematic review of ambient temperature exposure during pregnancy and stillbirth: Methods and evidence. *Environ Res.* 2021; 197:111037.

273. Song X, Jiang L, Zhang D, Wang X, Ma Y, Hu Y*, et al.* Impact of short-term exposure to extreme temperatures on diabetes mellitus morbidity and mortality? A systematic review and meta-analysis. *Environ Sci Pollut Res Int.* 2021; 28(41):58035-58049.

274. Weilnhammer V, Schmid J, Mittermeier I, Schreiber F, Jiang L, Pastuhovic V*, et al.* Extreme weather events in europe and their health consequences - A systematic review. *Int J Hyg Environ Health.* 2021; 233:113688.

275. Zafeiratou S, Samoli E, Dimakopoulou K, Rodopoulou S, Analitis A, Gasparrini A*, et al.* A systematic review on the association between total and cardiopulmonary mortality/morbidity or cardiovascular risk factors with long-term exposure to increased or decreased ambient temperature. *Sci Total Environ.* 2021; 772:145383.

276. Liang M, Ding X, Wu Y, Sun Y. Temperature and risk of infectious diarrhea: a systematic review and meta-analysis. *Environ Sci Pollut Res Int.* 2021; 28(48):68144-68154.

277. Li Y, Dou Q, Lu Y, Xiang H, Yu X, Liu S. Effects of ambient temperature and precipitation on the risk of dengue fever: A systematic review and updated meta-analysis. *Environ Res.* 2020; 191:110043.

278. Grigorieva E, Lukyanets A. Combined Effect of Hot Weather and Outdoor Air Pollution on Respiratory Health: Literature Review. *Atmosphere.* 2021; 12(6).

279. Areal AT, Zhao Q, Wigmann C, Schneider A, Schikowski T. The effect of air pollution when modified by temperature on respiratory health outcomes: A systematic review and meta-analysis. *Sci Total Environ.* 2022; 811:152336.

280. Song X, Hu Y, Ma Y, Jiang L, Wang X, Shi A*, et al.* Is short-term and long-term exposure to black carbon associated with cardiovascular and respiratory diseases? A systematic review and meta-analysis based on evidence reliability. *BMJ Open.* 2022; 12(5):e049516.

281. Chandra M, Rai CB, Kumari N, Sandhu VK, Chandra K, Krishna M*, et al.* Air Pollution and Cognitive Impairment across the Life Course in Humans: A Systematic Review with Specific Focus on Income Level of Study Area. *Int J Environ Res Public Health.* 2022; 19(3).

282. Huang C, Li C, Zhao F, Zhu J, Wang S, Sun G. The Association between Childhood Exposure to Ambient Air Pollution and Obesity: A Systematic Review and Meta-Analysis. *Int J Environ Res Public Health.* 2022; 19(8).

283. Smaller L, Batra M, Erbas B. The Effect of Outdoor Environmental Exposure on Readmission Rates for Children and Adolescents with Asthma-A Systematic Review. *Int J Environ Res Public Health.* 2022; 19(12).

284. Yu X, Rahman MM, Wang Z, Carter SA, Schwartz J, Chen Z*, et al.* Evidence of susceptibility to autism risks associated with early life ambient air pollution: A systematic review. *Environ Res.* 2022; 208:112590.

285. Hu X, Han W, Wang Y, Aunan K, Pan X, Huang J*, et al.* Does air pollution modify temperature-related mortality? A systematic review and meta-analysis. *Environ Res.* 2022; 210:112898.

286. Ziou M, Tham R, Wheeler AJ, Zosky GR, Stephens N, Johnston FH. Outdoor particulate matter exposure and upper respiratory tract infections in children and adolescents: A systematic review and meta-analysis. *Environ Res.* 2022; 210:112969.

287. Sun HZ, Yu P, Lan C, Wan MWL, Hickman S, Murulitharan J*, et al.* Cohort-based long-term ozone exposure-associated mortality risks with adjusted metrics: A systematic review and meta-analysis. *Innovation (Camb).* 2022; 3(3):100246.

288. Markozannes G, Pantavou K, Rizos EC, Sindosi O, Tagkas C, Seyfried M*, et al.* Outdoor air quality and human health: An overview of reviews of observational studies. *Environ Pollut.* 2022; 306:119309.

289. Liu J, Varghese BM, Hansen A, Zhang Y, Driscoll T, Morgan G*, et al.* Heat exposure and cardiovascular health outcomes: a systematic review and meta-analysis. *The Lancet Planetary Health.* 2022; 6(6):e484-e495.

290. Perry T, Obolski U, Peretz C. The association between high ambient temperature and mortality in the Mediterranean basin: a systematic review and meta-analysis. *medRxiv.* 2022:01.20.22269580.

291. Jia X, Shen Z, Liu R, Han Y, Yang Y, Chen Q*, et al.* Association of fine particulate matter to allergic rhinitis: A systematic review and meta-analysis. *European Journal of Inflammation.* 2022.

292. Hua W, Li X, Chu X, Cao N, Wu H, Huang R*, et al.* Ambient air pollutants increase the risk of Immunoglobulin E-mediated allergic diseases: a systematic review and meta-analysis. 2021.

293. de Bont J, Jaganathan S, Dahlquist M, Persson A, Stafoggia M, Ljungman P. Ambient air pollution and cardiovascular diseases: An umbrella review of systematic reviews and meta-analyses. *J Intern Med.* 2022; 291(6):779-800.

294. Chen S, Huang L, Cai D, Li B, Yang J. Association between meteorological factors and COVID-19: a systematic review. *Int J Environ Health Res.* 2022:1-15.

295. Li N, Ma J, Ji K, Wang L. Association of PM2.5 and PM10 with Acute Exacerbation of Chronic Obstructive Pulmonary Disease at lag0 to lag7: A Systematic Review and Meta-Analysis. *COPD.* 2022; 19(1):243-254.

296. Heo S, Son JY, Lim CC, Fong KC, Choi HM, Hernandez-Ramirez RU*, et al.* Effect modification by sex for associations of fine particulate matter (PM2.5) with cardiovascular mortality, hospitalization, and emergency room visits: systematic review and meta-analysis. *Environ Res Lett.* 2022; 17(5).

297. Pritchett N, Spangler EC, Gray GM, Livinski AA, Sampson JN, Dawsey SM*, et al.* Exposure to Outdoor Particulate Matter Air Pollution and Risk of Gastrointestinal Cancers in Adults: A Systematic Review and Meta-Analysis of Epidemiologic Evidence. *Environ Health Perspect.* 2022; 130(3):36001.

298. Zhang J, Wang X, Yan M, Shan A, Wang C, Yang X*, et al.* Sex Differences in Cardiovascular Risk Associated With Long-Term PM2.5 Exposure: A Systematic Review and Meta-Analysis of Cohort Studies. *Front Public Health.* 2022; 10:802167.

299. Lin L, Li T, Sun M, Liang Q, Ma Y, Wang F*, et al.* Global association between atmospheric particulate matter and obesity: A systematic review and meta-analysis. *Environ Res.* 2022; 209:112785.

300. Guo X, Song Q, Wang H, Li N, Su W, Liang M*, et al.* Systematic review and meta-analysis of studies between short-term exposure to ambient carbon monoxide and non-accidental, cardiovascular, and respiratory mortality in China. *Environ Sci Pollut Res Int.* 2022; 29(24):35707-35722.

301. Yang Z, Mahendran R, Yu P, Xu R, Yu W, Godellawattage S*, et al.* Health Effects of Long-Term Exposure to Ambient PM2.5 in Asia-Pacific: a Systematic Review of Cohort Studies. *Curr Environ Health Rep.* 2022; 9(2):130-151.

302. Khosravipour M, Safari-Faramani R, Rajati F, Omidi F. The long-term effect of exposure to respirable particulate matter on the incidence of myocardial infarction: a systematic review and meta-analysis study. *Environ Sci Pollut Res Int.* 2022; 29(28):42347-42371.

303. Zang ST, Wu QJ, Li XY, Gao C, Liu YS, Jiang YT*, et al.* Long-term PM2.5 exposure and various health outcomes: An umbrella review of systematic reviews and meta-analyses of observational studies. *Sci Total Environ.* 2022; 812:152381.

304. Rasking L, Vanbrabant K, Bove H, Plusquin M, De Vusser K, Roels HA*, et al.* Adverse Effects of fine particulate matter on human kidney functioning: a systematic review. *Environ Health.* 2022; 21(1):24.

305. Dimala CA, Kadia BM. A systematic review and meta-analysis on the association between ambient air pollution and pulmonary tuberculosis. *Sci Rep.* 2022; 12(1):11282.

306. Guo X, Su W, Wang H, Li N, Song Q, Liang Q*, et al.* Short-term exposure to ambient ozone and cardiovascular mortality in China: a systematic review and meta-analysis. *Int J Environ Health Res.* 2022:1-18.

307. Holm SM, Balmes JR. Systematic Review of Ozone Effects on Human Lung Function, 2013 Through 2020. *Chest.* 2022; 161(1):190-201.

308. Lin LZ, Zhan XL, Jin CY, Liang JH, Jing J, Dong GH. The epidemiological evidence linking exposure to ambient particulate matter with neurodevelopmental disorders: A systematic review and meta-analysis. *Environ Res.* 2022; 209:112876.

309. Xu E, Li Y, Li T, Li Q. Association between ambient temperature and ambulance dispatch: a systematic review and meta-analysis. *Environ Sci Pollut Res Int.* 2022.

310. Yue D, Shen T, Mao J, Su Q, Mao Y, Ye X*, et al.* Prenatal exposure to air pollution and the risk of eczema in childhood: a systematic review and meta-analysis. *Environ Sci Pollut Res Int.* 2022; 29(32):48233-48249.

311. Manullang A, Lee Y-L, Laiman V, Chang J-H, Chuang H-C. Associations between Ozone and Emphysema: A Systematic Review and Meta-analysis. *Aerosol and Air Quality Research.* 2022; 22:220027.

312. Gasana J, Dillikar D, Mendy A, Forno E, Ramos Vieira E. Motor vehicle air pollution and asthma in children: a meta-analysis. *Environ Res.* 2012; 117:36-45.

313. Liu FX, Chang-Richards A, Wang KIK, Dirks KN. Effects of climate change on health and wellbeing: A systematic review. *Sustainable Development.*

314. Mason H, King JC, Peden AE, Franklin RC. Systematic review of the impact of heatwaves on health service demand in Australia. *Bmc Health Services Research.* 2022; 22(1).

315. Arsad FS, Hod R, Ahmad N, Ismail R, Mohamed N, Baharom M*, et al.* The Impact of Heatwaves on Mortality and Morbidity and the Associated Vulnerability Factors: A Systematic Review. *International Journal of Environmental Research and Public Health.* 2022; 19(23).

316. Faurie C, Varghese BM, Liu JW, Bi P. Association between high temperature and heatwaves with heat-related illnesses: A systematic review and meta-analysis. *Science of the Total Environment.* 2022; 852.

317. Han AZ, Deng SZ, Yu JR, Zhang YL, Jalaludin B, Huang CR. Asthma triggered by extreme temperatures: From epidemiological evidence to biological plausibility. *Environmental Research.* 2023; 216.

318. Benmarhnia T, Deguen S, Kaufman JS, Smargiassi A. Vulnerability to Heat-related Mortality A Systematic Review, Meta-analysis, and Meta-regression Analysis. *Epidemiology.* 2015; 26(6):781-793.

319. Cong XW, Xu XJ, Zhang YL, Wang QH, Xu L, Huo X. Temperature drop and the risk of asthma: a systematic review and meta-analysis. *Environmental Science and Pollution Research.* 2017; 24(28):22535-22546.

320. Lakhoo DP, Blake HA, Chersich MF, Nakstad B, Kovats S. The Effect of High and Low Ambient Temperature on Infant Health: A Systematic Review. *International Journal of Environmental Research and Public Health.* 2022; 19(15).

321. Liu JW, Varghese BM, Hansen A, Xiang JJ, Zhang Y, Dear K*, et al.* Is there an association between hot weather and poor mental health outcomes? A systematic review and meta-analysis. *Environment International.* 2021; 153.

322. Zheng HL, Guo ZL, Wang ML, Yang C, An SY, Wu W. Effects of climate variables on the transmission of COVID-19: a systematic review of 62 ecological studies. *Environmental Science and Pollution Research.* 2021; 28(39):54299-54316.

323. Wang P, Zhang XY, Hashizume M, Goggins WB, Luo C. A systematic review on lagged associations in climate-health studies. *International Journal of Epidemiology.* 2021; 50(4):1199-1212.

324. Wu K, Ho HC, Su H, Huang CR, Zheng H, Zhang WY*, et al.* A systematic review and meta-analysis of intraday effects of ambient air pollution and temperature on cardiorespiratory morbidities: First few hours of exposure matters to life. *Ebiomedicine.* 2022; 86.

325. Islam MM, Noor FM. Correlation between COVID-19 and weather variables: A meta-analysis. *Heliyon.* 2022; 8(8).

326. Wang T, Wang J, Rao J, Han Y, Luo Z, Jia L*, et al.* Meta-analysis of the effects of ambient temperature and relative humidity on the risk of mumps. *Sci Rep.* 2022; 12(1):6440.

327. Manyuchi AE, Chersich M, Vogel C, Wright CY, Matsika R, Erasmus B. Extreme heat events, high ambient temperatures and human morbidity and mortality in Africa: A systematic review. *South African Journal of Science.* 2022; 118(11/12):24-31.

328. Lee W-S, Kim W-S, Lim Y-H, Hong Y-C. High Temperatures and Kidney Disease Morbidity: A Systematic Review and Meta-analysis. *Journal of preventive medicine and public health = Yebang Uihakhoe chi.* 2019; 52(1):1-13.

329. Gao D, Friedman S, Hosler A, Sheridan S, Zhang W, Lin S. Association between extreme ambient heat exposure and diabetes-related hospital admissions and emergency department visits: A systematic review. *Hyg Environ Healh Adv.* 2022; 4.

330. Krittanawong C, Qadeer YK, Hayes RB, Wang Z, Virani S, Thurston GD*, et al.* PM2.5 and Cardiovascular Health Risks. *Curr Probl Cardiol.* 2023:101670.

331. Pyo JS, Kim NY, Kang DW. Impacts of Outdoor Particulate Matter Exposure on the Incidence of Lung Cancer and Mortality. *Medicina (Kaunas).* 2022; 58(9).

332. Hernandez Carballo I, Bakola M, Stuckler D. The impact of air pollution on COVID-19 incidence, severity, and mortality: A systematic review of studies in Europe and North America. *Environ Res.* 2022; 215(Pt 1):114155.

333. Rezayat AA, Niloufar J, Mir Nourbakhsh SH, Hasheminezhad Hoseini FS, Hooshmand N, Ghasemi Nour M*, et al.* The effect of air pollution on systemic lupus erythematosus: A systematic review and meta-analysis. *Lupus.* 2022; 31(13):1606-1618.

334. Gan T, Bambrick H, Tong S, Hu W. Air pollution and liver cancer: A systematic review. *J Environ Sci (China).* 2023; 126:817-826.

335. Juneja Gandhi T, Garg PR, Kurian K, Bjurgert J, Sahariah SA, Mehra S*, et al.* Outdoor Physical Activity in an Air Polluted Environment and Its Effect on the Cardiovascular System-A Systematic Review. *Int J Environ Res Public Health.* 2022; 19(17).

336. Badida P, Krishnamurthy A, Jayaprakash J. Meta analysis of health effects of ambient air pollution exposure in low- and middle-income countries. *Environ Res.* 2023; 216(Pt 4):114604.

337. Zhang D, Chen W, Cheng C, Huang H, Li X, Qin P*, et al.* Air pollution exposure and heart failure: A systematic review and meta-analysis. *Sci Total Environ.* 2023; 872:162191.

338. Podury S, Kwon S, Javed U, Farooqi MS, Li Y, Liu M*, et al.* Severe Acute Respiratory Syndrome and Particulate Matter Exposure: A Systematic Review. *Life (Basel).* 2023; 13(2).

339. Wang Q, Cao J. Atmospheric PM(2.5) exposure and risk of ischemic heart disease: A systematic review and meta-analysis of observational studies. *Perfusion.* 2022:2676591221131485.

340. Ju L, Hua L, Xu H, Li C, Sun S, Zhang Q*, et al.* Maternal atmospheric particulate matter exposure and risk of adverse pregnancy outcomes: A meta-analysis of cohort studies. *Environ Pollut.* 2023; 317:120704.

341. Luo H, Zhang Q, Niu Y, Kan H, Chen R. Fine particulate matter and cardiorespiratory health in China: A systematic review and meta-analysis of epidemiological studies. *J Environ Sci (China).* 2023; 123:306-316.

342. Zhu X, Liu B, Guo C, Li Z, Cheng M, Zhu X*, et al.* Short and long-term association of exposure to ambient black carbon with all-cause and cause-specific mortality: A systematic review and meta-analysis. *Environ Pollut.* 2023; 324:121086.

343. Yang YS, Pei YH, Gu YY, Zhu JF, Yu P, Chen XH. Association between short-term exposure to ambient air pollution and heart failure: An updated systematic review and meta-analysis of more than 7 million participants. *Front Public Health.* 2022; 10:948765.

344. Yang M, Wu KY, Wu QY, Huang CR, Xu ZW, Ho HC*, et al.* A systematic review and meta-analysis of air pollution and angina pectoris attacks: identification of hazardous pollutant, short-term effect, and vulnerable population. *Environmental Science and Pollution Research.*

345. Chung CY, Yang J, Yang XG, He J. Long-term effects of ambient air pollution on lung cancer and COPD mortalities in China: A systematic review and meta-analysis of cohort studies. *Environmental Impact Assessment Review.* 2022; 97.

346. Husaini DC, Reneau K, Balam D. Air pollution and public health in Latin America and the Caribbean (LAC): a systematic review with meta-analysis. *Beni-Suef University Journal of Basic and Applied Sciences.* 2022; 11(1).

347. Ruan FF, Zeng XA. Health Effects of PM2.5 Exposure in China from 2004 to 2018: A Systematic Review and Meta-Analysis. *Sustainability.* 2023; 15(1).

348. Tabaei S, Hadei M, Pasalari N, Panahande M, Tabaee SS. Association between short-term exposure to PM and cardiovascular mortality in Iran: a systematic review and meta-analysis. *Air Quality Atmosphere and Health.*

349. Zhang TF, Mao WH, Gao JH, Song XQ, Li LF, Sun XB*, et al.* The effects of PM2.5 on lung cancer-related mortality in different regions and races: A systematic review and meta-analysis of cohort studies. *Air Quality Atmosphere and Health.* 2022; 15(9):1523-1532.

350. Zhang WJ, Ma RM, Wang YW, Jiang N, Zhang Y, Li TT. The relationship between particulate matter and lung function of children: A systematic review and meta-analysis. *Environmental Pollution.* 2022; 309.

351. Sui J, Xia H, Zhao Q, Sun GJ, Cai YY. Long-Term Exposure to Fine Particulate Matter and the Risk of Chronic Liver Diseases: A Meta-Analysis of Observational Studies. *International Journal of Environmental Research and Public Health.* 2022; 19(16).

352. Zhai G, Tian Y, Zhang Y, Zhou W. The effect of ambient temperature and risk of cardiovascular disease hospitalization in China: a meta-analysis. *Int J Biometeorol.* 2023; 67(9):1423-1433.

353. Wen J, Zou L, Jiang Z, Li Y, Tao J, Liu Y*, et al.* Association between ambient temperature and risk of stroke morbidity and mortality: A systematic review and meta-analysis. *Brain Behav.* 2023; 13(7):e3078.

354. Li D, Zhang Y, Li X, Zhang K, Lu Y, Brown RD. Climatic and meteorological exposure and mental and behavioral health: A systematic review and meta-analysis. *Sci Total Environ.* 2023; 892:164435.

355. Fan JF, Xiao YC, Feng YF, Niu LY, Tan X, Sun JC*, et al.* A systematic review and meta-analysis of cold exposure and cardiovascular disease outcomes. *Front Cardiovasc Med.* 2023; 10:1084611.

356. Pan R, Xie M, Chen M, Zhang Y, Ma J, Zhou J. The impact of heat waves on the mortality of Chinese population: A systematic review and meta-analysis. *Medicine (Baltimore).* 2023; 102(13):e33345.

357. Jia Y, Lin Z, He Z, Li C, Zhang Y, Wang J*, et al.* Effect of Air Pollution on Heart Failure: Systematic Review and Meta-Analysis. *Environ Health Perspect.* 2023; 131(7):76001.

358. Luben TJ, Wilkie AA, Krajewski AK, Njie F, Park K, Zelasky S*, et al.* Short-term exposure to air pollution and infant mortality: A systematic review and meta-analysis. *Sci Total Environ.* 2023; 898:165522.

359. Sheppard N, Carroll M, Gao C, Lane T. Particulate matter air pollution and COVID-19 infection, severity, and mortality: A systematic review and meta-analysis. *Sci Total Environ.* 2023; 880:163272.

360. Wan X, Wei S, Wang Y, Jiang J, Lian X, Zou Z*, et al.* The association between maternal air pollution exposure and the incidence of congenital heart diseases in children: A systematic review and meta-analysis. *Sci Total Environ.* 2023; 892:164431.

361. Zhang S, Fu Q, Wang S, Jin X, Tan J, Ding K*, et al.* Association between air pollution and the prevalence of allergic rhinitis in Chinese children: A systematic review and meta-analysis. *Allergy Asthma Proc.* 2022; 43(5):e47-e57.

362. Yu K, Zhang Q, Wei Y, Chen R, Kan H. Global association between air pollution and COVID-19 mortality: A systematic review and meta-analysis. *Sci Total Environ.* 2023:167542.

363. Guo J, Chai G, Song X, Hui X, Li Z, Feng X*, et al.* Long-term exposure to particulate matter on cardiovascular and respiratory diseases in low- and middle-income countries: A systematic review and meta-analysis. *Front Public Health.* 2023; 11:1134341.

364. Wang W, Mu S, Yan W, Ke N, Cheng H, Ding R. Prenatal PM2.5 exposure increases the risk of adverse pregnancy outcomes: evidence from meta-analysis of cohort studies. *Environ Sci Pollut Res Int.* 2023.

365. Samoli E, Rodopoulou S, Schneider A, Morawska L, Stafoggia M, Renzi M*, et al.* Meta-analysis on short-term exposure to ambient ultrafine particles and respiratory morbidity. *Eur Respir Rev.* 2020; 29(158).

366. McDermott-Levy R, Scolio M, Shakya KM, Moore CH. Factors That Influence Climate Change-Related Mortality in the United States: An Integrative Review. *Int J Environ Res Public Health.* 2021; 18(15).

367. Ohlwein S, Kappeler R, Kutlar Joss M, Künzli N, Hoffmann B. Health effects of ultrafine particles: a systematic literature review update of epidemiological evidence. *International journal of public health.* 2019; 64(4):547-559.

368. Liu C, Jia F, Ji M, Qu G, Ye C, Cheng J*, et al.* The Effect of Short-Term Air Pollutants Exposure on Daily Mortality Among Elderly Individuals in China: A Systematic Review and Meta-Analysis. *Water, Air, and Soil Pollution.* 2023; 234(8).

369. Huang M, Chen JY, Yang YP, Yuan H, Huang ZJ, Lu Y. Effects of Ambient Air Pollution on Blood Pressure Among Children and Adolescents: A Systematic Review and Meta-Analysis. *Journal of the American Heart Association.* 2021; 10(10).

370. Huang SH, Zhang XY, Huang JF, Lu XF, Liu FC, Gu DF. Ambient air pollution and body weight status in adults: A systematic review and meta-analysis. *Environmental Pollution.* 2020; 265.

371. Huang JJ, Yang XY, Fan FF, Hu Y, Wang X, Zhu SN*, et al.* Outdoor air pollution and the risk of asthma exacerbations in single lag0 and lag1 exposure patterns: a systematic review and meta-analysis. *Journal of Asthma.* 2021.

372. Fu L, Chen Y, Yang XY, Yang ZY, Liu S, Pei L*, et al.* The associations of air pollution exposure during pregnancy with fetal growth and anthropometric measurements at birth: a systematic review and meta-analysis. *Environmental Science and Pollution Research.* 2019; 26(20):20137-20147.

373. Borroni E, Pesatori AC, Bollati V, Buoli M, Carugno M. Air pollution exposure and depression: A comprehensive updated systematic review and meta-analysis. *Environmental Pollution.* 2022; 292.

374. Lin CK, Chang YT, Lee FS, Chen ST, Christiani D. Association between exposure to ambient particulate matters and risks of autism spectrum disorder in children: a systematic review and exposure-response meta-analysis. *Environmental Research Letters.* 2021; 16(6).

375. Hu CY, Fang Y, Li FL, Dong B, Hua XG, Jiang W*, et al.* Association between ambient air pollution and Parkinson's disease: Systematic review and meta-analysis. *Environmental Research.* 2019; 168:448-459.

376. Ngoc LTN, Park D, Lee Y, Lee YC. Systematic Review and Meta-Analysis of Human Skin Diseases Due to Particulate Matter. *International Journal of Environmental Research and Public Health.* 2017; 14(12).

377. Ni Y, Song W, Bai Y, Liu T, Li GX, Bian Y*, et al.* Years of Life Lost (YLL) Due to Short-Term Exposure to Ambient Air Pollution in China: A Systematic Review and Meta-Analysis. *International Journal of Environmental Research and Public Health.* 2021; 18(21).

378. Juarez PD, Ramesh A, Hood DB, Alcendor DJ, Valdez RB, Aramandla MP*, et al.* The effects of air pollution, meteorological parameters, and climate change on COVID-19 comorbidity and health disparities: A systematic review. *Environmental Chemistry and Ecotoxicology.* 2022; 4:194-210.

379. Oliveira J, Base LH, de Abreu LC, Ferreira C, Ferreira C, Morawska L. Ultrafine particles and children's health: Literature review. *Paediatric Respiratory Reviews.* 2019; 32:73-81.

380. Yu HB, Yin YX, Zhang JS, Zhou R. The impact of particulate matter 2.5 on the risk of preeclampsia: an updated systematic review and meta-analysis. *Environmental Science and Pollution Research.* 2020; 27(30):37527-37539.

381. Fan SJ, Heinrich J, Bloom MS, Zhao TY, Shi TX, Feng WR*, et al.* Ambient air pollution and depression: A systematic review with meta-analysis up to 2019. *Science of the Total Environment.* 2020; 701.

382. Liu QSJ, Wang WZ, Gu XL, Deng FR, Wang XQ, Lin HL*, et al.* Association between particulate matter air pollution and risk of depression and suicide: a systematic review and meta-analysis. *Environmental Science and Pollution Research.* 2021; 28(8):9029-9049.

383. Tsoli S, Ploubidis GB, Kalantzi OI. Particulate air pollution and birth weight: A systematic literature review. *Atmospheric Pollution Research.* 2019; 10(4):1084-1122.

384. Han F, Yang XY, Xu DG, Wang Q, Xu DQ. Association between outdoor PM<sub>2.5</sub> and prevalence of COPD: a systematic review and meta-analysis. *Postgraduate Medical Journal.* 2019; 95(1129):612-618.

385. Zang ST, Luan J, Li L, Wu QJ, Chang Q, Dai HX*, et al.* Air pollution and metabolic syndrome risk: Evidence from nine observational studies. *Environmental Research.* 2021; 202.

386. Yan W, Wang X, Dong TY, Sun MQ, Zhang MZ, Fang KC*, et al.* The impact of prenatal exposure to PM<sub>2.5</sub> on childhood asthma and wheezing: a meta-analysis of observational studies. *Environmental Science and Pollution Research.* 2020; 27(23):29280-29290.

387. Ju LL, Li CL, Yang M, Sun S, Zhang Q, Cao JY*, et al.* Maternal air pollution exposure increases the risk of preterm birth: Evidence from the meta-analysis of cohort studies. *Environmental Research.* 2021; 202.

388. Fadadu RP, Chee E, Jung A, Chen JY, Abuabara K, Wei ML. Air pollution and global healthcare use for atopic dermatitis: A systematic review. *Journal of the European Academy of Dermatology and Venereology.* 2023.

389. Huang J, Zheng WH, Huang HC, Ran YG, Liu Y, Huang P. Particulate matter, nitrogen dioxide, and sulfur dioxide and their associations with allergic skin diseases: A systematic review and meta-analysis. *Atmospheric Pollution Research.* 2023; 14(7).

390. Liu YF, Li Y, Xu HL, Zhao XY, Zhu YW, Zhao BS*, et al.* Pre- and postnatal particulate matter exposure and blood pressure in children and adolescents: A systematic review and meta-analysis. *Environmental Research.* 2023; 223.

391. Parasin N, Amnuaylojaroen T, Saokaew S. Exposure to PM<sub>10</sub>, PM<sub>2.5</sub>, and NO<sub>2</sub> and gross motor function in children: a systematic review and meta-analysis. *European Journal of Pediatrics.* 2023.

392. Liu HX, Ding L, Qu GB, Guo XW, Liang MM, Ma SD*, et al.* Particulate matter exposure during pregnancy and infancy and risks of autism spectrum disorder in children: A systematic review and meta-analysis. *Science of the Total Environment.* 2023; 855.

393. Tandon S, Grande AJ, Karamanos A, Cruickshank JK, Roever L, Mudway IS*, et al.* Association of Ambient Air Pollution with Blood Pressure in Adolescence: A Systematic-review and Meta-analysis. *Current Problems in Cardiology.* 2023; 48(2).

394. Karimi B, Samadi S. Mortality associated with fine particulate and its components: A systematic review and meta-analysis. *Atmospheric Pollution Research.* 2023; 14(2).

395. Zong ZQ, Zhao MJ, Zhang MY, Xu KX, Zhang YQ, Zhang XJ*, et al.* Association between PM<sub>1</sub> Exposure and Lung Function in Children and Adolescents: A Systematic Review and Meta-Analysis. *International Journal of Environmental Research and Public Health.* 2022; 19(23).

396. Blanc N, Liao JW, Gilliland F, Zhang JF, Berhane K, Huang GY*, et al.* A systematic review of evidence for maternal preconception exposure to outdoor air pollution on Children's health. *Environmental Pollution.* 2023; 318.

397. Liu J, Chen YM, Liu D, Ye F, Sun Q, Huang Q*, et al.* Prenatal exposure to particulate matter and term low birth weight: systematic review and meta-analysis. *Environmental Science and Pollution Research.* 2023; 30(23):63335-63346.

398. Bouchriti Y, Haddou MA, Kabbachi B, Achbani A, Cherrat Z, Rida J*, et al.* Ambient Air Quality and Health Impact of Exposure to Outdoor Air Pollution in the Moroccan Population: A Systematic Review. *Pollution.* 2023; 9(2):660-677.

399. Cheng S, Jin Y, Dou Y, Zhao Y, Duan Y, Pei H*, et al.* Long-term particulate matter 2.5 exposure and dementia: a systematic review and meta-analysis. *Public Health.* 2022; 212:33-41.

400. Zhang YM, Guo ZY, Zhang W, Li QY, Zhao Y, Wang ZL*, et al.* Effect of Acute PM2.5 Exposure on Lung Function in Children: A Systematic Review and Meta-Analysis. *Journal of Asthma and Allergy.* 2023; 16:529-540.

401. Yu SR, Zhang MZ, Zhu JM, Yang X, Bigambo FM, Snijders AM*, et al.* The effect of ambient ozone exposure on three types of diabetes: a meta-analysis. *Environmental Health.* 2023; 22(1).

402. Bougea A, Papagiannakis N, Simitsi AM, Panagiotounakou E, Chrysovitsanou C, Angelopoulou E*, et al.* Ambiental Factors in Parkinson's Disease Progression: A Systematic Review. *Medicina-Lithuania.* 2023; 59(2).

403. Wu QY, Yang M, Wu KY, Su H, Huang CR, Xu ZW*, et al.* Abnormal ambient temperature change increases the risk of out-of-hospital cardiac arrest: A systematic review and meta-analysis of exposure types, risk, and vulnerable populations. *Science of the Total Environment.* 2023; 861.

404. Puthota J, Alatorre A, Walsh S, Clemente JC, Malaspina D, Spicer J. Prenatal ambient temperature and risk for schizophrenia. *Schizophrenia Research.* 2022; 247:67-83.

405. Thompson R, Lawrance EL, Roberts LF, Grailey K, Ashrafian H, Maheswaran H*, et al.* Ambient temperature and mental health: a systematic review and meta-analysis. *Lancet Planetary Health.* 2023; 7(7).
